# Synthesis of Antiproliferative *N*-Chalconyl Imidazolidones and Mechanistic Evaluation of Prototype *N*-CIMZ 1 as a Macrophage-Modulating Antimitotic Agent

**DOI:** 10.3390/ph19101503

**Published:** 2026-09-22

**Authors:** Atziri Corin Chavez Alvarez, Lisa Treboux, Céline Audrey Béchon-Diot, Rayan Chkair, Sophie Besse, Emmanuel Moreau

**Affiliations:** UMR-1240 IMoST INSERM, Université Clermont Auvergne, 58 rue Montalembert, 63005 Clermont-Ferrand, France; lisa.treboux@etu.uca.fr (L.T.); celine_audrey.bechon_diot@uca.fr (C.A.B.-D.); rayan.chkair@uca.fr (R.C.); sophie.besse@inserm.fr (S.B.)

**Keywords:** breast cancer, antimitotic agents, chalcones, imidazolidones, *N*-chalconylimidazolidones, macrophages, immunomodulation

## Abstract

**Background/Objectives:** To address current aggressive breast cancer challenges, a novel series of eight *N*-chalconyl imidazolidone derivatives (*N*-CIMZs **1**–**8**) was synthesized and evaluated for direct cytotoxicity and immunomodulation via macrophage polarization. **Methods:** Following antiproliferative screening against four representative breast cancer lines, the prototype candidate *N*-CIMZ **1** was selected for cell cycle and scratch-wound healing assays in MCF-7 cells. Multi-parametric flow cytometry on human THP-1 macrophages tracked cellular stress (γH2AX), metabolic markers (iNOS), and surface dynamics (LAP (TGF-β1), CD163, CD206, CD209). **Results:** *N*-CIMZ **1** acted as an antimitotic agent, inducing G2/M phase arrest (51.0%) and restricting MCF-7 wound confluence to 33% at 24 h. In THP-1 macrophages, it maintained low induction of γH2AX-mediated DNA damage signaling. Under alternative M2 pressure, *N*-CIMZ **1** intercepted polarization by forcing high membrane retention of the immunosuppressive peptide LAP (81%) and suppressing the scavenger marker CD163 (12%) while buffering hyper-reactive CD206+/CD209+ hybrid spikes down to 2.33% under M1 stress. **Conclusions:** *N*-CIMZ **1** represents a promising multi-modal scaffold combining antimitotic and macrophage-modulating properties. This targeted immunomodulation effectively subverts tumor-supportive macrophage phenotypes, showcasing the potential of microenvironment reprogramming to counter breast cancer progression and metastasis.

## 1. Introduction

Breast cancer (BC) remains one of the most prevalent malignancies and a leading cause of cancer-related mortality among women worldwide [1]. Despite significant diagnostic and therapeutic advancements, managing advanced stages and aggressive molecular subtypes such as triple-negative breast cancer (TNBC) presents a severe clinical challenge [2,3]. This difficulty is largely driven by the frequent emergence of multidrug resistance (MDR) against conventional chemotherapeutic agents, including cisplatin, carboplatin, and doxorubicin [4,5]. While these standard regimens are initially effective, their clinical utility is heavily restricted by severe systemic toxicities. For instance, platinum-based drugs exert their cytotoxicity primarily through non-selective DNA adduct formation and crosslinking, whereas anthracyclines like doxorubicin induce multi-targeted cellular stress via DNA intercalation, topoisomerase II poisoning, and reactive oxygen species (ROS) generation [6,7,8]. Consequently, the long-term prognosis for high-risk, chemoresistant breast malignancies remains poor, underscoring an urgent biopharmaceutical need for innovative, alternative therapeutic strategies that combine high oncological efficacy with a minimized collateral toxicity profile. In this clinical landscape, antimitotic small molecules represent a highly successful class of chemotherapeutics utilized for decades in oncology [9,10,11]. Clinically established agents such as paclitaxel, vinblastine, and the structural reference standard combretastatin A-4 (CA-4) are cell-cycle specific, selectively arresting rapidly dividing populations at the G2/M phase [12]. However, conventional antimitotics frequently suffer from limitations such as low water solubility, susceptibility to efflux pump-mediated resistance (e.g., P-glycoprotein), and peripheral neurotoxicity [13,14]. Therefore, exploring novel antimitotic scaffolds remains a powerful avenue for discovering next-generation derivatives characterized by improved therapeutic indexes, the capacity to cross cellular resistance barriers, and reduced structural damage to healthy tissues. Addressing this structural objective, a novel family of antimitotic compounds designated as *N*-chalconyl imidazolidones (*N*-CIMZs) has been developed based on the well-studied chalcone scaffold that has historically been used in pharmaceutical development [15].

Structurally, the molecular design of our proposed compounds is based on the hybridization of carefully selected pharmacophores to achieve a synergistic biological profile. First, the chalcone framework was selected as the structural backbone due to its documented versatility and broad spectrum of anticancer (e.g., metochalcone, SD400, Figure 1) and immunomodulatory (e.g., licochalcone A, Figure 1) properties [16,17,18,19]. To optimize target affinity and cellular permeability, substituents on the aromatic B-ring (Figure 1) were strategically chosen to cover a range of electronic and steric profiles, aligning with well-established structure–activity relationship (SAR) principles for antineoplastic agents. Notably, this includes the 3,4,5-trimethoxy moiety derived from the CA-4 structure (Figure 1) [10], alongside other validated substituents drawn from known antimitotic molecules featuring similar aromatic systems (e.g., PAIB-SAs, Figure 1) [20]. To complement this scaffold, we incorporated an imidazolidone core, a motif validated as a privileged structure in oncology such as *N*-arylsulfonylimidazolidinone, to ensure high target selectivity and optimal stability. By integrating this core, our design exploits inherent conformational rigidity and hydrogen-bonding capacity to facilitate stable interactions within the target sites [21,22,23]. Ultimately, our rationale transcends simple antimitotic inhibition; we aim to develop compounds with a dual mechanism of action. We hypothesized that combining the chalcone pharmacophore, known for its broad anticancer and immunomodulatory properties, with a conformationally constrained imidazolidinone scaffold could generate a new class of hybrid compounds with complementary biological properties. This structural hybridization was therefore explored as a strategy to develop antimitotic agents with the potential to simultaneously modulate tumor cell proliferation and the tumor immune microenvironment. By combining the optimized chalcone motif with the imidazolidone core, we hypothesize that these molecules may simultaneously induce cell cycle arrest and exert immunomodulatory effects, potentially interfering with pro-tumoral signaling pathways in the tumor microenvironment. This multi-target structural approach provides a robust foundation for evaluating these hybrid agents as potential antineoplastic candidates, specifically investigating their proliferative and immunomodulatory activity. Crucially, emerging paradigms in oncology dictate that highly effective, long-term anticancer strategies must extend beyond direct tumor cytotoxicity. Importantly, effective anticancer strategies increasingly consider not only the direct targeting of tumor cells but also the complex cellular interactions within the tumor microenvironment (TME) [24,25]. Antimitotic agents can effectively interfere with tumor cell proliferation by disrupting mitotic progression; however, the biological response to anticancer therapy is also influenced by the surrounding TME. Tumor-associated macrophages (TAMs) represent a major immune-cell population in many tumors and display considerable functional plasticity. Depending on the signals present within the TME, macrophages can acquire tumor-supportive functions, including immunosuppression, extracellular matrix remodeling, and promotion of angiogenesis and tumor progression. Consequently, modulating macrophage functional states may represent a complementary strategy to the direct antimitotic activity of anticancer agents, potentially providing a broader impact on both tumor cells and their microenvironment. TAMs are frequently hijacked by cancer-derived cytokines (such as IL-4 and IL-13), forcing them to adopt an alternative M2-polarized state. These M2-like cells establish an immunosuppressive shield, drive extracellular matrix remodeling, promote angiogenesis, and actively protect the tumor mass from therapeutic intervention [26,27]. Consequently, developing multi-modal compounds capable of exerting direct antimitotic control over cancer cells while concurrently targeting macrophage plasticity to prevent or subvert M2 polarization represents a major therapeutic approach to dismantle the protective shield of the TME. Herein, we report the synthesis and biological evaluation of a novel family of *N*-chalconyl imidazolidone derivatives derived from the structural hybridization of the well-studied chalcone and imidazolidone pharmacophores.

We initially demonstrated their antiproliferative profiling across a diverse human breast cancer cell panel representing the major clinical and molecular subtypes (MCF-7 for Luminal A, BT-20 and MDA-MB-231 for triple-negative phenotypes, and MDA-MB-453 for HER2-positive tumors) and characterized the functional impact of the prototype candidate, *N*-CIMZ **1**, on cell cycle distribution and inhibition of in vitro scratch-wound closure. Furthermore, we expanded the biological characterization of this scaffold by implementing a multi-parametric flow cytometry assessment in human THP-1-derived macrophages, tracking key phenotypic and regulatory markers including LAP (TGF-β)[28], CD163 [29], CD206 [30], CD209 [31], iNOS [32], and γH2AX [33], providing a comprehensive overview of its dual-action potential as both a direct antimitotic agent and an immunomodulatory regulator.

## 2. Results

### 2.1. Synthesis

The synthesis of *N*-CIMZs (**1**–**8**) was performed through a three-step process (Figure 2). Briefly, 1-(3-aminophenyl)ethanone (**9**) or 1-(4-aminophenyl)ethanone (**10**) (1.0 eq) was added to a solution of methylene chloride and 2-chloroethyl isocyanate (1.0 eq) at room temperature and was stirred overnight to afford the corresponding 1-(3-acetylphenyl)-3-(2-chloroethyl)urea (**11**) and 1-(4-acetylphenyl)-3-(2-chloroethyl)urea (**12**), respectively. Subsequently, **11** or **12** (1.0 eq) were treated overnight under reflux at 60 °C in a solution of tetrahydrofuran by adding sodium methanolate (1.2 eq), promoting the cyclization of the chloroethyl moiety to yield either 1-(4-acetylphenyl)imidazolidin-2-one (**13**) or 1-(3-acetylphenyl)imidazolidin-2-one (**14**). Finally, *N*-CIMZs **1**–**8** were obtained by treating either **13** or **14** (1.0 eq) with sodium hydroxide (10% (*w*/*v*), 4.0 eq) in methanol, in the presence of the appropriate substituted aldehyde (1.0 eq) overnight under reflux at 50 °C overnight.

### 2.2. Antiproliferative Activities

The synthesized derivatives (*N*-CIMZs **1**–**8**) were evaluated for their antiproliferative potential against a panel of four human breast cancer cell lines displaying distinct molecular profiles to comprehensively evaluate their cytotoxic spectrum. This panel included MCF-7 (a hormone-dependent, Luminal A subtype), BT-20 and MDA-MB-231 (representing basal-like and highly metastatic triple-negative breast cancer [TNBC] phenotypes, respectively), and MDA-MB-453 (characterizing a HER2-positive, androgen receptor-expressing subtype), using combretastatin A-4 (CA-4) as the reference standard (Table 1). The screening revealed that the derivatives exhibit cytotoxic activity within the micromolar to sub-micromolar range, with potency patterns heavily dictated by both the core structural substitution architecture and the specific cellular context.

The antiproliferative screening highlighted a clear dependence on the geometric configuration of ring A (1,3- versus 1,4-disubstitution) and the electronic nature of the functional groups appended to ring B. Derivatives bearing a 3,4,5-trimethoxy (3,4,5-OMe) arrangement on ring B (*N*-CIMZs **1** and **5**) consistently yielded high potency across the panel. *N*-CIMZ **1** emerged as the prototype molecule, displaying sub-micromolar toxicity in MCF-7 (0.5 ± 0.1 µM) and MDA-MB-453 (0.4 ± 0.1 µM) profiles. Direct comparison between structural analogues showed that the 1,3-substitution pattern on ring A is generally more favorable for enhancing cytotoxicity. This is evidenced by comparing *N*-CIMZ **1** against its 1,4-disubstituted counterpart, *N*-CIMZ **5**, where the 1,3-regiochemistry enhanced potency in MDA-MB-453 cells (0.4 ± 0.1 µM vs. 1.4 ± 0.7 µM). However, this trend was not absolute, as *N*-CIMZ **7** (1,4-disubstituted) retained strong selective potency in MCF-7 cells (0.6 ± 0.1 µM). The introduction of electron-withdrawing groups on ring B altered the antiproliferative profile. Dichloro-substituted derivatives (*N*-CIMZs **2** and **6**) managed moderate to strong potency, particularly in MDA-MB-453 cells (0.6 ± 0.3 µM and 0.5 ± 0.2 µM, respectively).

Conversely, the difluoro-substituted analogues (*N*-CIMZs **4** and **8**) ranked as the least active across the series, showing substantial loss of activity in MDA-MB-231 cells (>5.0 µM and >2.0 µM). Alkylic modifications via 2,5-dimethyl (2,5-Me) substitutions (*N*-CIMZs **3** and **7**) resulted in highly cell line-dependent profiles. *N*-CIMZ **3** exhibited its maximum cytotoxicity in BT-20 (0.4 ± 0.3 µM) and MDA-MB-453 (0.3 ± 0.2 µM) environments but fell sharply to a restricted phenotype in MDA-MB-231 layers (>2.0 µM). The biological response profile varied extensively across the cell panel. The triple-negative MDA-MB-231 cell line demonstrated a generalized recalcitrance across the entire chemical series, with four out of eight compounds failing to cross the 2.0 µM threshold (>2.0 µM, > 5.0 µM). Conversely, the MCF-7 and MDA-MB-453 cell lines were significantly more sensitive to the chemical challenge. While the reference standard CA-4 maintained its established baseline dominance within the low nanomolar threshold, *N*-CIMZ **1** was successfully prioritized as the prototype candidate due to its balanced 1,3-/3,4,5-OMe scaffold and its antiproliferative activity in the analyzed cellular models.

Based on the initial antiproliferative screening, the hormone-sensitive MCF-7 cell line demonstrated high sensitivity to *N*-CIMZ **1** treatment. Consequently, subsequent functional evaluations including kinetic wound-healing assays and complex macrophage-immunomodulation studies were selectively pursued using the MCF-7 framework. This strategic approach allowed for the precise characterization of the compound’s dual-front mechanism of action within a responsive cellular model representing the most clinically prevalent breast cancer subtype while establishing a controlled therapeutic window to investigate macrophage reprogramming without the confounding variables of baseline drug resistance.

### 2.3. Scratch-Wound Healing Assay

To determine whether the biological activity of the prioritized prototype candidate extends beyond baseline cytotoxicity into the modulation of cell gap closure, a scratch-wound healing assay was performed using the human breast adenocarcinoma cell line MCF-7. Based on its consistent broad-spectrum efficacy and optimal balanced pharmacological profile across the evaluated breast cancer cell lines, *N*-CIMZ **1** was selected as the representative prototype compound for all subsequent advanced functional and mechanistic investigations, including wound healing, cell-cycle analysis, microtubule imaging, and macrophage modulation assays. Collective scratch-wound closure dynamics were tracked and quantified by measuring wound confluence percentages across a 36-h kinetic profile, comparing *N*-CIMZ **1** (2.5 µM) against untreated cells, vehicle controls (DMSO 0.2%), and CA-4 at 125 nM as the reference standard (Figure 3A) this acute supramaximal concentration of 125 nM was selected for wound closure assays to ensure rapid and maximal pharmacological response, which describes the observed dose–response behavior without implying saturation of a specific molecular target, and allow the assessment of early effects. The experimental concentration of 2.5 µM for *N*-CIMZ **1** was selected because it represents a 5-fold concentration of its 48 h IC_50_ value in MCF-7 cells. This specific 5x threshold was chosen to guarantee robust, reproducible engagement with downstream therapeutic targets during kinetic assays, ensuring a distinct biological response while maintaining the compound within a non-DNA-damaging profile that avoids non-specific off-target cytotoxicity.

During the initial hours post-scratch (0 to 6 h), all experimental groups initiated a sharp upward proliferation into the denuded area. At the 12-h milestone (Figure 3B), untreated control cells reached a mean wound confluence of 33.7 ± 4.5%, a footprint tightly mirrored by the vehicle control group (DMSO 0.2%), which exhibited a confluence of 34 ± 6%. Although the reference standard CA-4 presented the lowest confluence at this time point (27.9 ± 0.4%), the wound-closure inhibitory effect of both CA-4 and *N*-CIMZ **1** (31.3 ± 1.6%) did not clear the threshold for statistical significance against the resting control layers, marking an early phase of unrestricted initial wound closure. A crucial kinetic shift and stabilization occurred beyond the 12-h mark, where the wound-closure curve of *N*-CIMZ **1**-treated cells began to flatten distinctly. At the 24-h milestone (Figure 3B), untreated cells reached a confluence of 37.2 ± 1.9%, while vehicle-treated cells (DMSO 0.2%) continued their progressive monolayer repair, advancing to a wound confluence of 48 ± 11%. In sharp contrast, *N*-CIMZ **1** actively arrested this progression, restricting wound confluence to 33 ± 1%. This stabilization was statistically significant against the vehicle control (** *p* < 0.01) and successfully matched the inhibitory performance of the reference standard CA-4 (34 ± 1%).

This persistent inhibitory trend was fully consolidated at the late-phase endpoint of 36 h (Figure 3B). While the untreated and vehicle control monolayers expanded steadily toward total confluence, reaching maximum values of 45 ± 3% and 53 ± 11%, respectively, the cells challenged with *N*-CIMZ **1** remained strictly arrested. At this final milestone, *N*-CIMZ **1** consistently maintained the lowest wound confluence rate across all conditions (37 ± 2%), yielding a robust and statistically significant restriction of wound closure compared to the vehicle-treated control (** *p* < 0.01). Taken together, these kinetic data demonstrate that *N*-CIMZ **1** exerts a time-dependent, persistent, and sustained inhibitory effect on MCF-7 wound closure, confirming its capacity to block functional tumor cell motility over extended periods.

### 2.4. Cell Cycle Progression

To investigate whether the antiproliferative and wound-closure inhibitory effects of our prioritized candidate are linked to the disruption of mitotic progression, cell cycle distribution was quantified in MCF-7 cells via flow cytometry. The profiling compared the effects of *N*-CIMZ **1** (2.5 µM) against the reference standard CA-4 at 125 nM, the vehicle control (DMSO 0.2%), and untreated baseline populations (Figure 4).

Untreated control cells and those exposed to the vehicle control (DMSO 0.2%) displayed a canonical, asynchronous cell cycle distribution profile characterized by a dominant population in the resting/growth phases. The homeostatic baseline parameters for the vehicle control group settled at 48.5 ± 1.5% of cells residing in the G0/G1 phase, 19 ± 1% undergoing DNA replication in the S phase, and a steady 32 ± 2% in the G2/M phase. No statistically significant variations were observed between the untreated cells and the vehicle-treated cells, confirming that the vehicle baseline does not exert independent genotoxic or antimitotic pressure.

In sharp contrast, challenging the breast cancer cells with the reference agent CA-4 induced a distinct and highly significant phenotypic shift. Under CA-4 treatment, the cell fraction in the G0/G1 phase fell significantly to 28 ± 3% (* *p* < 0.05), while the S phase remained statistically unaltered (20 ± 1%). This contraction was balanced by a prominent accumulation of cells within the mitotic checkpoint, driving the G2/M population up to 53 ± 2%, establishing a highly significant mitotic arrest profile against the vehicle control (** *p* < 0.01).

Crucially, *N*-CIMZ **1** successfully replicated this characteristic antimitotic profile, reflecting the cellular effects observed in our experiments without implying identification of a specific molecular target. Treatment with the selected derivative drove a significant decrease in the G0/G1 reservoir down to 32 ± 7% (* *p* < 0.05) while leaving the DNA synthesis fraction intact in the S phase (17.2 ± 0.5%). Mirroring the performance of the reference standard, *N*-CIMZ **1** concentrated the cellular population within the mitotic boundary, driving the G2/M phase up to 51 ± 6%. This targeted accumulation represents a robust and statistically highly significant checkpoint delay compared directly to the vehicle-treated control group (** *p* < 0.01). These quantitative shifts demonstrate that *N*-CIMZ **1** operates as a potent inhibitor of cellular division by actively locking regular cell cycle progression at the G2/M phase.

### 2.5. Disruption of the Microtubule Network

To determine whether the G2/M cell cycle arrest induced by *N*-CIMZ **1** is associated with a direct microtubule perturbation, we evaluated its effects on microtubule organization using immunofluorescence confocal microscopy. As shown in Figure 5, untreated and vehicle-treated (DMSO 0.2%) MCF-7 cells displayed a normal, dense, and well-organized cytoplasmic microtubule network with intact nuclear morphology. In contrast, treatment with the reference antimitotic compound Combretastatin A-4 (CA-4, 125 nM) induced marked microtubule depolymerization and cytoskeletal collapse. Notably, treatment with *N*-CIMZ **1** (2.5 µM) triggered a pronounced disruption of the microtubule architecture that closely mirrored the phenotypic alterations observed with CA-4.

These findings, together with the G2/M accumulation observed by flow cytometry, support the involvement of microtubule-dependent mitotic processes in the antiproliferative activity of *N*-CIMZ **1** and are consistent with an antimitotic mechanism of action.

### 2.6. Analysis of the Effect of N-CIMZ ***1*** on Macrophage Polarization Antigens

To evaluate how *N*-CIMZ **1** reshapes the macrophage functional landscape in the TME, a comprehensive flow cytometry panel of surface and intracellular markers was designed. The human THP-1 cell line was selected as a reproducible and well-established in vitro model of human macrophage differentiation, providing a standardized experimental system that minimizes the donor-to-donor variability associated with primary monocyte-derived macrophages, and is suitable for the pharmacological evaluation of macrophage responses. This panel systematically evaluates lineage commitment (CD14), classical co-stimulation (CD86), alternative activation and endocytosis (CD206, CD209, and CD163), active immunosuppressive cytokine tethering (LAP(TGF-β1)), metabolic inflammation (iNOS), and cellular genotoxic stress (γH2AX). By challenging these THP-1-derived macrophages with specific polarizing conditions (LPS + IFN-γ for M1; IL-4 + IL-13 for M2) in the presence or absence of *N*-CIMZ **1**, we aimed to dissect whether this novel molecule could successfully obstruct tumor-supportive alternative differentiation and reprogram the TME toward an active immune state.

#### 2.6.1. Baseline Characterization: Lineage Verification and Initial Activation Profile of M0 Macrophages

Prior to evaluating the modulation of alternative polarization, we established the baseline phenotypic identity and activation status of the differentiated THP-1 cells by quantifying the lineage marker CD14 and the co-stimulatory molecule CD86 in the resting M0 phase (Figure 6A,B). The basal expression of CD14 is high, showing a mean of 95.17 ± 1.36% in the untreated group and 95.71 ± 0.68% in the DMSO 0.2% control. Exposure to either the M1 cocktail (LPS+IFN-γ: 94.02 ± 0.72%) or the M2 cocktail (IL-4 + IL-13: 94.79 ± 0.53%) does not drastically alter the percentage of CD14^+^ cells. Treatment with *N*-CIMZ **1**, both alone (93.59 ± 1.14%) and across co-treatments, maintains a stable and high percentage of positive cells, matching all other experimental groups. Basal levels of CD86 are moderate in the untreated group (33.51 ± 1.56%). The vehicle control (DMSO 0.2%) induces a slight background increase, raising expression to 41.45 ± 1.14%. The classical M1 stimulus (LPS+IFN) robustly upregulates CD86 to its maximum mean of 75.38 ± 0.94%, while the M2 stimulus (IL4+IL13) keeps it at a lower baseline of 45.47 ± 1.23%. *N*-CIMZ **1** alone significantly upregulates CD86 expression to 62.47 ± 1.63% compared to its direct vehicle control (DMSO 0.2%), positioning it at an intermediate-to-high activation state.

#### 2.6.2. Phenotypic Analysis of Alternative, Immunoregulatory, and Metabolic Markers in Differentiated THP-1 Cells

To further dissect the pharmacological scope of *N*-CIMZ **1** beyond its antiproliferative and cell cycle-arresting effects observed in MCF-7 cells, we evaluated its capacity to modulate immune plasticity within the TME. Within this niche, tumor-associated macrophages (TAMs) exhibit high plasticity, broadly polarizing into two functionally distinct phenotypes: the classically activated M1 macrophages, which exert pro-inflammatory and antitumoral activities, and the alternatively activated M2 macrophages, which promote an anti-inflammatory, protumoral microenvironment [34]. While M1 macrophages actively restrict cancer progression by executing direct tumoricidal functions and priming adaptive immunity, M2 macrophages facilitate tumor cell propagation, extracellular matrix remodeling, and metastatic dissemination [27,35]. Given that aggressive tumor cells actively recruit and polarize macrophages toward this permissive M2 state to subvert immune surveillance, we hypothesized that the anti-proliferative pressure exerted by *N*-CIMZ **1** might be complemented by the direct modulation of macrophage functional states, specifically by altering surface marker expression to restrict the protumoral M2 profile. To assess this, human THP-1 monocytes were differentiated with PMA into resting M0 macrophages and subsequently challenged with specific polarizing conditions: LPS + IFN-γ for the pro-inflammatory M1 phenotype and IL-4 + IL-13 for the immunosuppressive M2 phenotype. Using single- and double-marker flow cytometry, we quantified the expression of a dedicated panel of surface and intracellular proteins following treatment with *N*-CIMZ **1** at 2.5 µM. This targeted panel included the alternative scavenger receptor CD163, the alternative lectin co-receptor CD209 (DC-SIGN), the mannose receptor CD206, the membrane-anchored latency-associated peptide LAP (TGF-β1), and the cytotoxic metabolic enzyme iNOS. This specific multi-parametric approach allowed us to determine whether *N*-CIMZ **1** can actively obstruct tumor-supportive alternative polarization and suppress immunosuppressive cytokine tethering, thereby acting as a dual-action antimitotic and immunomodulatory scaffold. To determine the effects of *N*-CIMZ **1** on alternative polarization, the major M2 surface markers CD163 and CD209 (DC-SIGN) were analyzed under protective and interleukin-stimulated conditions. The baseline surface expression of the alternative scavenger receptor CD163 (Figure 7A,B) was low in the control groups, yielding percentages of 7.02 ± 0.34% for untreated cells and 9 ± 1% for the vehicle control (DMSO 0.2%). As expected, alternative M2 polarization driven by the IL-4 + IL-13 cytokine cocktail successfully upregulated the marker, nearly doubling its expression to 15 ± 2%. Monotherapy with *N*-CIMZ **1** at 2.5 µM shifted the resting baseline to 18 ± 1%. Crucially, when *N*-CIMZ **1** was integrated into the alternative activation conditions (IL-4 + IL-13 + *N*-CIMZ **1**), it effectively subverted alternative differentiation, significantly abating the cytokine-induced upregulation and pulling the percentage of positive cells back down to 12 ± 3%.

The membrane presence of the alternative polarization marker CD209 (Figure 8A,B) stood at 55 ± 1% in untreated cells, while the vehicle control shifted the baseline to 66 ± 2%. Under alternative M2 polarizing conditions (IL-4 + IL-13), CD209 expression was driven to its maximum assay threshold of 71 ± 1%. Monotherapy with *N*-CIMZ **1** actively suppressed this marker’s baseline down to 44 ± 3%. Notably, when *N*-CIMZ **1** was added alongside the alternative activation cocktail (IL-4 + IL-13 + *N*-CIMZ **1**), it exerted a strong antagonistic effect against alternative differentiation, significantly decreasing CD209-positive cells to 56 ± 2%. Conversely, challenging the macrophages with the classical M1 cocktail (LPS + IFN-γ) resulted in moderate CD209 expression levels of 56 ± 2%, and the addition of the investigational compound (LPS + IFN-γ + *N*-CIMZ **1**) did not induce significant variations, maintaining a stable percentage of positive cells at 52 ± 1%. This demonstrates that *N*-CIMZ **1**-mediated regulation of CD209 is selective for the alternative differentiation pathway.

The surface peptidic remodeling and homeostatic buffering capacity of *N*-CIMZ **1** were evaluated by analyzing the expression of LAP (TGF-β1) and CD206, respectively. Regarding LAP (TGF-β1) dynamics (Figure 9A,B), resting M0 macrophages exhibited prominent constitutive surface retention, yielding values of 88.3 ± 0.3% in untreated cells and a maximum baseline of 93.50 ± 0.40% in the vehicle control (DMSO 0.2%). Monotherapy with *N*-CIMZ **1** at 2.5 µM induced a reduction in surface presence, establishing a baseline of 72 ± 2%. When inducing polarization pathways, both classical M1 (LPS + IFN-γ) and alternative M2 (IL-4 + IL-13) cytokine cocktails caused a substantial and comparable decrease in surface-bound LAP (TGF-β1), dropping to 67.01 ± 0.42% and 70 ± 2%, respectively, relative to the vehicle control. Notably, when *N*-CIMZ **1** was integrated into the alternative activation conditions (IL-4 + IL-13 + *N*-CIMZ **1**), it effectively prevented the cytokine-driven reduction in surface fluorescence, forcing the cells to retain LAP (TGF-β1) at a high membrane density of 81 ± 2%, thereby halting the drop observed with IL-4 + IL-13 stimulation alone. Conversely, in the pro-inflammatory co-treatment group (LPS + IFN-γ + *N*-CIMZ **1**), LAP(TGF-β1) membrane presence remained stable at 73 ± 2%, mirroring the levels observed during *N*-CIMZ **1** monotherapy.

Simultaneously, the baseline surface expression of CD206 was found to be low in the control conditions, yielding percentages of 4.3 ± 0.8% for untreated cells and 3.5 ± 0.6% for the DMSO vehicle (Figure 10A,B). Treatment with *N*-CIMZ **1** alone slightly shifted this baseline to 12 ± 1%, matching the levels observed when the compound was combined with the alternative cocktail in the IL-4 + IL-13 + *N*-CIMZ **1** group (12 ± 1%). Of note, the alternative M2 conditioning cocktail (IL-4 + IL-13) on its own induced only a subtle variation to 8.3 ± 0.3%. Within this THP-1 model, challenging the macrophages with the classical M1 cocktail (LPS + IFN-γ) triggered a sharp, well-documented phenotypic upregulation, maximizing CD206 expression at 29 ± 1%. Crucially, when *N*-CIMZ **1** was combined with this classical inflammatory stimulus (LPS + IFN-γ + *N*-CIMZ **1**), it exerted a strong homeostatic buffering effect, significantly attenuating the M1-induced protein spike and lowering the percentage of positive cells to 18 ± 1%.

The intracellular expression of the inducible nitric oxide synthase (iNOS) enzyme was quantified across all experimental conditions to evaluate baseline activation and potential metabolic shifts induced by the treatments (Figure 11A,B). The proportion of iNOS-positive cells remained heavily saturated across the board, moving within a very narrow and high threshold across all groups. In the baseline control groups, differentiated untreated cells exhibited an iNOS expression percentage of 97.1 ± 0.4%, which was statistically comparable to the baseline levels observed in the vehicle control group exposed to DMSO 0.2% (98.4 ± 0.4%). Monotherapy with *N*-CIMZ **1** at 2.5 µM maintained this constitutive profile, yielding a mean of 98.1 ±0.2% positive cells. When establishing polarization conditions, challenging the macrophages with the classical M1 conditioning cocktail (LPS + IFN-γ) resulted in a minor upward shift, maximizing the population positivity at 99.08 ± 0.28%. Conversely, the alternative M2 stimulus (IL-4 + IL-13) on its own led to a subtle, non-significant variation, establishing a mean expression of 97.4 ± 0.9%. The integration of the investigational compound into these active environments did not trigger any significant architectural or phenotypic deviations from the constitutive maximum expression profile. Specifically, the pro-inflammatory co-treatment group (LPS + IFN-γ + *N*-CIMZ **1**) displayed a percentage positivity of 98.7 ± 0.2%, while the alternative co-treatment group (IL-4 + IL-13 + *N*-CIMZ **1**) stabilized at 97.6 ± 1.1%. This homogeneous saturation across all eight conditions indicates that neither the cytokine stimuli nor *N*-CIMZ **1** treatments alter the high baseline percentage of iNOS-positive cells established during the initial monocyte differentiation phase. The very high baseline iNOS signal observed in untreated M0 macrophages resulted in a severely restricted dynamic range, limiting the usefulness of iNOS as a quantitative marker of M1 polarization under our experimental conditions. The precise origin of this high intracellular signal could not be conclusively established and may reflect non-specific/background staining associated with intracellular detection and/or the differentiation procedure. Moreover, the THP-1 model has recognized limitations for the induction and assessment of the human NOS2/iNOS pathway. Therefore, iNOS was not considered a reliable standalone marker of classical M1 activation in this model. M1 polarization was instead assessed using surface CD86 expression, which displayed a clear and significant increase following classical activation (Figure 6B).

#### 2.6.3. Evaluation of Cellular Stress and Double-Strand DNA Breaks via γH2AX Profiling

To monitor structural cell duress, double-strand DNA breaks, and potential genotoxic stress, γH2AX expression percentages were quantified across all experimental conditions, utilizing a 400 µM H_2_O_2_ treatment as a validated positive control [36,37] to maximize and anchor the genotoxic response at 48 ± 1% (Figure 12A,B). In the baseline control groups, differentiated untreated M0 cells exhibited a baseline γH2AX positivity of 31.5 ± 4.5%, which was statistically comparable to the moderate background stress levels observed in the vehicle control group exposed to DMSO 0.2% (26 ± 2%). Monotherapy with *N*-CIMZ **1** at 2.5 µM maintained a lack of γH2AX induction, yielding a mean of 18.4 ± 4.6% positive cells, showing no significant elevation or γH2AX-mediated DNA-damage signaling compared to the vehicle control. Under active polarization conditions, distinct cytokine-driven dynamics were observed. Classical M1 conditioning with the LPS + IFN-γ cocktail suppressed γH2AX down to 16.4 ± 0.5%. Conversely, alternative M2 stimulation with the IL-4 + IL-13 cocktail significantly upregulated the stress marker, pushing the percentage of positive cells to 40.3 ± 4.5%. The integration of the investigational compound into these polarized environments showed a pathway-specific behavior. In the pro-inflammatory co-treatment group (LPS + IFN-γ + *N*-CIMZ **1**), γH2AX expression remained low and stable at 17 ± 2%, mirroring the levels of the M1 stimulus alone. Similarly, the addition of the compound to the alternative conditions (IL-4 + IL-13 + *N*-CIMZ **1**) left the elevated cytokine-induced stress signature statistically unchanged, stabilizing at a mean population positivity of 39.5 ± 10.1%.

#### 2.6.4. Co-Expression Profiling of CD206 and CD209 via Dual-Marker Flow Cytometry

To map the shifts in macrophage subpopulation dynamics and track the potential emergence of hybrid activation phenotypes, a dual-parametric quadrant analysis of CD206 versus CD209 was performed across all experimental groups (Figure 13A,B). In the resting state, untreated M0 macrophages clustered into two distinct, nearly equal populations: a double-negative CD206^−^/CD209^−^ resting pool (49.3 ± 0.7%) and a single-positive CD206^−^/CD209^+^ alternative pool (50.3% ± 0.7%), with negligible baseline presence in the CD206^+^/CD209^+^ double-positive (0.35 ± 0.05%) or CD206^+^/CD209^−^ single-positive (0.06 ± 0.02%) quadrants. Exposure to the vehicle control (DMSO 0.2%) shifted this baseline distribution, increasing the single-positive CD206^−^/CD209^+^ population to 63.7 ± 1.3% while contracting the double-negative reservoir to 36.1 ± 1.3%. Crucially, monotherapy with *N*-CIMZ **1** at 2.5 µM actively inverted this vehicle-induced trend. The investigational compound robustly expanded the double-negative resting population to a maximum of 62.7 ± 3.1%, and successfully suppressed the single-positive CD206^−^/CD209^+^ baseline population down to 35.8 ± 3.1% while keeping the double-positive population strictly limited (1.3 ± 0.1%). Challenging the THP-1-derived macrophages with the classical pro-inflammatory M1 cocktail (LPS + IFN-γ) drove a marked phenotypic shift. While the double-negative CD206^−^/CD209^−^ pool stood at 53.4 ± 1.8% and the single-positive CD206^−^/CD209^+^ pool dropped to 40.5 ± 2.2%, the M1 stimulus forced the emergence of a prominent hybrid double-positive CD206^+^/CD209^+^ subpopulation, peaking at 6.1 ± 0.4%. Notably, when *N*-CIMZ **1** was combined with the classical inflammatory environment (LPS + IFN-γ + *N*-CIMZ **1**), it successfully exercised a potent buffering effect against this activation spike. The compound significantly blunted the expansion of the hybrid double-positive cluster, cutting its presence down to 2.3 ± 0.4%, effectively mitigating the hyper-reactive inflammatory shift.

When exposed to the alternative M2 conditioning cocktail (IL-4 + IL-13), the cellular architecture tightly mirrored the vehicle-treated state, pushing the single-positive tumor-supportive CD206^−^/CD209^+^ population to a maximum threshold of 63.5 ± 1.7% while restricting the double-negative population to 35.3 ± 1.7% and maintaining a minimal double-positive background (1.1 ± 0.1%). Crucially, the addition of the investigational compound to these alternative conditions (IL-4 + IL-13 + *N*-CIMZ **1**) successfully subverted the M2-driven polarization. The co-treatment prevented the accumulation of the tumor-supportive alternative phenotype, pulling the CD206^−^/CD209^+^ cluster down to 48.6 ± 2.2%. This phenotypic suppression was direct and clean, as the cells were systematically steered back into the double-negative resting pool (49.7 ± 2.1%) without driving any significant or compensatory expansion of the double-positive hybrid quadrant (1.5 ± 0.1%).

#### 2.6.5. Coordinated Disruption of Macrophage Protumorigenic Scavenging and Suppressive Signaling Dynamics Dissected by LAP(TGF-β1) vs. CD163 Profiling

To evaluate the impact of *N*-CIMZ **1** on structural membrane features and alternative scavenger receptor dynamics, a dual-marker quadrant tracking of LAP(TGF-β1) vs. CD163 was executed across all experimental groups (Figure 14A,B). Differentiated resting M0 macrophages exhibited a highly consistent and asymmetric baseline distribution, with the vast majority of the population residing within the single-positive LAP(TGF-β1)^+^/CD163^−^ reservoir (84.4 ± 0.5% for untreated cells and 88 ± 1% for the vehicle control). Double-negative LAP(TGF-β1)^−^/CD163^−^ cells constituted a small, stable basal subpopulation (11 ± 1% in untreated; 6.01 ± 0.16% in DMSO), while populations expressing surface CD163 remained strictly constrained, yielding low values in both the double-positive LAP(TGF-β1)^+^/CD163^+^ (4.5 ± 0.2% vs. 6.1 ± 0.8%), and single-positive LAP(TGF-β1)^−^/CD163^+^ (0.17 ± 0.05% for both) quadrants. Monotherapy with *N*-CIMZ **1** at 2.5 µM actively altered this structural landscape. The compound promoted a controlled reduction in the dominant LAP(TGF-β1)^+^/CD163^−^ pool down to 69.7 ± 2.4%, which was balanced by a proportional expansion of both the double-negative LAP(TGF-β1)^−^/CD163^−^ reservoir (18.03 ± 1.33%) and the single-positive LAP(TGF-β1)^−^/CD163^+^ population (9.3 ± 0.3%).

Challenging the cellular matrix with the pro-inflammatory M1 cocktail (LPS + IFN-γ) triggered a uniform shift away from the resting state. The pro-inflammatory stimulus targeted the main LAP(TGF-β1)^+^/CD163^−^ reservoir, compressing its presence down to 65.9 ± 0.6% while driving a substantial expansion of the double-negative LAP(TGF-β1)^−^/CD163^−^ pool to 28.9 ± 0.2%. Under these classical conditions, the single-positive LAP(TGF-β1)^−^/CD163^+^ fraction experienced a subtle increase to 3.7 ± 0.2%, whereas the double-positive LAP(TGF-β1)^+^/CD163^+^ subpopulation dropped to its lowest assay threshold of 1.9 ± 0.3%. Notably, in the inflammatory co-treatment group (LPS + IFN-γ + *N*-CIMZ **1**), the compound successfully modulated this activation transition. *N*-CIMZ **1** partially restored the LAP(TGF-β1)^+^/CD163^−^ pool up to 71.3 ± 1.1%, and effectively mitigated the expansion of the double-negative cluster, lowering its proportion back to 20.5 ± 2.0%. Simultaneously, the addition of the compound resulted in a stable percentage of single-positive LAP(TGF-β1)^−^/CD163^+^ cells (6.1 ± 0.4%) and a slight, non-significant variation in the double-positive LAP(TGF-β1)^+^/CD163^+^ quadrant, which stood at 2.1 ± 0.9%. Exposing the macrophages to the alternative M2-polarizing conditions (IL-4 + IL-13) led to a distinct phenotypic reconfiguration. The cytokine cocktail drove a marked reduction in the resting LAP(TGF-β1)^+^/CD163^−^ pool to 68 ± 2%, caused a major expansion of both the activated double-negative LAP(TGF-β1)^−^/CD163^−^ population (22.4 ± 1.5%), and the single-positive alternative LAP(TGF-β1)^−^/CD163^+^ quadrant, which peaked at 7.02 ± 1.26%. Under this alternative stimulation, the baseline double-positive LAP(TGF-β1)^+^/CD163^+^ cluster contracted down to 2.8 ± 0.3%. Crucially, the addition of the investigational compound to these alternative conditions (IL-4 + IL-13 + *N*-CIMZ **1**) successfully attenuated alternative M2 polarization through a coordinated phenotypic interception. The co-treatment actively blocked the cytokine-driven transition, forcing a massive, statistically significant retention of the population within the baseline LAP(TGF-β1)^+^/CD163^−^ quadrant, which rose to 78.4 ± 3.2%. This structural retention directly prevented the expansion of the tumor-supportive subpopulations, forcing a significant reduction in both the double-negative LAP(TGF-β1)^−^/CD163^−^ reservoir down to 14.2 ± 0.5%, and the single-positive alternative LAP(TGF-β1)^−^/CD163^+^ cluster down to 4.4 ± 1.5%. Interestingly, the double-positive LAP(TGF-β1)^+^/CD163^+^ subpopulation stabilized without significant elevation, yielding a mean population positivity of 3.04 ± 1.38%, confirming that *N*-CIMZ **1** blocks the alternative phenotype without forcing an accumulation of double-positive hybrid cells.

## 3. Discussion

### 3.1. Structure–Activity Relationships (SAR) and Differential Cytotoxicity Across Breast Cancer Subtypes

The in vitro antiproliferative profiling of this novel *N*-chalconyl imidazolidone series (*N*-CIMZs **1**–**8**) establishes a distinct, regiochemically driven structure–activity relationship (SAR) that highlights the critical interplay between electronic parameters and spatial conformation. In the framework of combretastatin-like scaffolds, high-affinity binding to cellular targets requires a strict spatial orientation of functional groups to mimic the structural layout of established antimitotic agents like CA-4 [38,39]. The biological data demonstrate that the presence of 3,4,5-OMe motif on ring B is an indispensable requirement for maximizing cytotoxicity in this scaffold. *N*-CIMZs **1** and **5** consistently exhibited the highest potency across the entire breast cancer panel. Structurally, this specific trimethoxy substitution pattern is well-documented to lock the phenyl ring into an optimal configuration that fits deep inside hydrophobic pockets of target proteins, such as the colchicine-binding site on tubulin, preventing the structural rotation of the aromatic system. However, the electronic contribution of ring B must be coordinated with a specific spatial geometry on ring A [40,41]. The direct comparison between the structural analogues *N*-CIMZ **1** (1,3-disubstituted) and **5** (1,4-disubstituted) reveals that the 1,3-regiochemistry on ring A imparts a superior conformational arrangement. This geometric orientation likely optimizes the dihedral angle between the structural rings, projecting the functional groups into a more favorable binding alignment [42]. This is clearly highlighted by the behavior of *N*-CIMZ **1** in MDA-MB-453 cells. In contrast to the trimethoxy motif, modifying ring B with electron-withdrawing halogens (*N*-CIMZs **2**, **4**, **6**, **8**) or alkylic groups (*N*-CIMZs **3**, **7**) resulted in a marked reduction or fragmentation of antiproliferative activity. While di-chlorination (*N*-CIMZs **2** and **6**) retained moderate sub-micromolar potency against the estrogen receptor-positive (ER^+^) MCF-7 line and the HER2^+^ MDA-MB-453 line, di-fluorination (*N*-CIMZs **4** and **8**) caused a widespread drop in activity. This implies that while increased lipophilicity via halogenation can assist membrane permeability, the substantial alteration of electrostatic surface potential and the reduced steric volume of fluorine atoms fail to fulfill the precise electronic requirements of the binding pocket [43,44]. The cellular context proved to be a dominant variable. The triple-negative breast cancer (TNBC) line MDA-MB-231 stood out as the most recalcitrant model in the study, showing high resistance to halogens and methyl substitutions. This resistant fingerprint is characteristic of TNBC, which frequently upregulates drug-efflux pumps (such as P-glycoprotein/ABCB1) and alters protein expression of the cytoskeleton [45]. Notably, *N*-CIMZ **1** managed to clear this defensive barrier, maintaining a potent micromolar footprint where other derivatives were inactive. This capacity to cross different breast cancer molecular subtypes without suffering from functional drop-off underscores the structural resilience of *N*-CIMZ **1** as a reference scaffold.

### 3.2. Kinetic Modulation and Restriction of Scratch-Wound Closure

The acquisition of scratch-wound closure capabilities is a fundamental requirement for the progression of malignant phenotypes, enabling consolidated cell layers to break architectural constraints and initiate invasion dynamics. Tracking these processes through time-dependent wound healing kinetics allows for the discrimination between immediate, transient alterations in cell movement and sustained, long-term blocks on collective cell motility.

During the early phase of the assay (0 to 12 h), all cell groups, including those treated with *N*-CIMZ **1**, initiated a comparable structural motility into the cleared scratch zone. This initial window of unrestricted wound closure suggests that the compound does not exert an immediate, non-specific physical barrier or acute membrane toxicity that mechanically blocks the cells upon exposure. However, a critical kinetic shift occurred beyond the 12-h mark, where the wound-healing inhibition curve of the *N*-CIMZ **1** cohort entered a prominent plateau. The vehicle control (DMSO 0.2%) expanded steadily toward wound repair, advancing to a higher confluence over time, while the layers treated with *N*-CIMZ **1** remained structurally restricted. This distinct behavior of the curve demonstrates that the primary scratch-wound closure inhibitory effect of *N*-CIMZ **1** requires an induction window to manifest fully. Once established, this block is persistent and sustained over time, effectively locking the cell front in an open-wound conformation. The delay in cell cycle progression often runs parallel to a general down-regulation of the coordinated cellular remodeling required for forward movement, effectively decoupling the signaling networks that drive directional collective motility [46,47]. By showing that the compound effectively reduces wound closure over an extended 36-h window without relying on immediate cytotoxicity, these findings confirm that the anti-cancer traits of *N*-CIMZ **1** extend beyond simple cell death. The compound successfully acts as a sustained suppressor of functional tumor cell dynamics, presenting a desirable profile for scaffolds aimed at limiting the spread and progression of breast cancer cultures. The reduced wound closure observed following treatment with *N*-CIMZ **1** should be interpreted in the context of its effects on both scratch-wound closure and proliferation. Although the scratch-wound assay provides evidence of impaired wound closure, the experimental design does not allow the specific contribution of cell migration to be separated from the antiproliferative effect of the compound. The pronounced microtubule disruption and associated G2/M arrest induced by *N*-CIMZ **1** are therefore likely to contribute substantially to the reduced wound closure observed over the 36-h period. Further experiments specifically designed to distinguish migratory activity from proliferation, such as assays performed under proliferation-inhibiting conditions or complementary migration assays, would be required to establish a specific anti-migratory effect.

### 3.3. Structural Impact on Cell Cycle Progression, G2/M Phase Alterations and the Microtubule Network

The monitoring of cell cycle distribution provides critical insights into the general cytostatic or cytotoxic mechanisms exerted by novel antiproliferative agents. In asynchronous cell populations, a synchronized accumulation at a specific checkpoint usually reflects cell stress or the functional impairment of key macromolecular structures required for transition into downstream phases, ultimately tracking back to cellular division bottlenecks.

The quantitative cell cycle data demonstrate that *N*-CIMZ **1** induces a distinct functional disruption within the MCF-7 framework, characterized by a prominent accumulation of cells at the G2/M phase boundary. While untreated and vehicle control cells exhibited a standard, steady-state distribution dominated by the G0/G1 growth phase, exposure to *N*-CIMZ **1** forced a noticeable reorganization of the cell population, concentrating the population in the G2/M compartment. This observed phenotypic profile closely resembles the biological footprint of the reference standard CA-4. The capacity of *N*-CIMZ **1** to achieve a comparable level of G2/M phase accumulation highlights its efficiency in delaying regular mitotic progression, aligning its overall functional outcome with the traits commonly described for chalcone-derived structures and related chemical scaffolds. The systematic depletion of the G0/G1 reservoir under *N*-CIMZ **1** treatment suggests a directional pathway: cells freely exit the initial growth phase and complete genomic replication, but encounter a robust operational barrier prior to completing cell division. This selective delay at the late stage of the cell cycle is highly relevant in oncology [48], as a prolonged accumulation at the G2/M gate limits overall population expansion and supports the cytostatic potential of *N*-CIMZ **1**, making it a valuable candidate for further investigation into multi-modal anticancer strategies.

To substantiate whether this prominent G2/M entrapment stems from a direct antimitotic action, subsequent confocal immunofluorescence analyses confirmed that *N*-CIMZ **1** profoundly disrupts the intracellular microtubule network, effectively mirroring the phenotypic collapse induced by the reference standard CA-4 and corroborating its antimitotic mechanism.

### 3.4. Dual-Action Paradigm: Integrating Antimitotic Efficacy and Immunomodulatory Reprogramming of Macrophages

The modern therapeutic paradigm in immuno-oncology recognizes that successfully eradicating solid tumors requires a coordinated, dual-front strategy which includes directly halting malignant cellular division while simultaneously resetting the immunosuppressive architecture of the TME [49]. TAMs are crucial players in this matrix, frequently hijacked by neoplastic signaling to adopt an alternative M2-polarized phenotype [50]. These M2-like cells express specific surface scaffolds that suppress T-cell activation and facilitate tissue remodeling [50]. The biological evaluation of *N*-CIMZ **1** demonstrates that this novel *N*-chalconyl imidazolidone does not merely operate as a traditional, non-specific cytostatic agent. Instead, it functions as a multi-modal homeostatic modulator capable of exerting direct antimitotic control over breast cancer cells while concurrently intercepting pro-tumorigenic macrophage polarization.

A critical requirement for any investigational scaffold transitioning between tumor-destruction and immune-activation models is the maintenance of a safe cellular safety profile within healthy or immune matrices [51]. In human THP-1 macrophages, exposure to *N*-CIMZ **1** yielded a low count of cells positive for the double-strand break marker γH2AX. This observation suggests that the compound’s activity is not driven by widespread, non-specific DNA fragmentation in these cells. Furthermore, the compound did not perturb the baseline levels of iNOS-positive cells under the evaluated polarization conditions, indicating that it does not induce a generalized suppression of this specific marker, rather than causing an unselective translational shutdown.

The most compelling evidence of the immunomodulatory capacity of *N*-CIMZ **1** lies in the dual-parametric co-expression mapping of LAP vs. CD163 under alternative M2 pressure (IL-4 + IL-13). In a pro-tumorigenic state, alternative macrophages typically downregulate Membrane-Bound LAP to release active TGF-β1 (a potent suppressor of adaptive immunity) and upregulate the scavenger receptor CD163 to clear extracellular debris and support tumor growth. *N*-CIMZ **1** directly countered this alternative transition through a coordinated structural interception, forcing a massive, statistically significant retention of the macrophage population within the baseline LAP^+^/CD163^−^ quadrant. By keeping LAP securely locked on the cell surface, as a structural proxy the compound correlates with a potentially limited bioavailability of free, immunosuppressive TGF-β1 in the surrounding matrix [52], though further signaling studies would be needed to definitively quantify its release. Crucially, this structural retention ran parallel to a clean contraction of the single-positive alternative LAP^−^/CD163^+^ cluster. This simultaneous suppression of the alternative scavenger identity demonstrates that *N*-CIMZ **1** effectively subverts the acquisition of stable, tumor-supportive traits.

Beyond blocking the M2 extreme, the therapeutic versatility of *N*-CIMZ **1** is further highlighted by its buffering capacity under hyper-reactive pro-inflammatory M1 stress (LPS + IFN-γ). In differentiated THP-1 cells, classical activation frequently induces an unexpected upregulation of CD206 alongside CD209, leading to the emergence of ambiguous, double-positive (CD206^+^/CD209^+^) hybrid subpopulations. These plastic, intermediate states are highly prevalent in the fluid landscape of the TME and are often associated with unpredictable clinical outcomes and therapeutic resistance [53,54,55]. When introduced into the M1 polarizing conditions, *N*-CIMZ **1** significantly blunted the expansion of this hybrid double-positive cluster, cutting its presence down to a restricted threshold. Rather than pushing these cells toward an alternative state, the compound systematically steered the population back into the clean, double-negative resting reservoir (CD206^−^/CD209^−^), stabilizing the cell layer.

This dual-front performance establishes a unique pharmacological profile for *N*-CIMZ **1**. While it reduces wound closure of breast adenocarcinoma cells and locks their division cycle at the G2/M transition boundary, it does not act as a blind poison toward surrounding immune elements. Instead, it operates as a modulator of macrophage surface phenotypes as it downregulates the tumor-associated scavenger receptor CD163 in alternative macrophages, retains their suppressive cytokines (LAP (TGF-β1)) at the membrane level, and dampens volatile inflammatory shifts. By simultaneously neutralizing tumor cell expansion and preventing the establishment of an immune-evasive cellular shield, *N*-CIMZ **1** emerges as a highly promising, multi-modal chemical scaffold tailored for modern combinatory anti-cancer regimens aimed at restoring immune competence within the tumor mass. Nevertheless, several limitations of the present study must be acknowledged. From a medicinal chemistry perspective, the Structure–Activity Relationship (SAR) trends identified herein are restricted to the specific substitution patterns of the synthesized *N*-CIMZ series. The observed association between this structural arrangement and antiproliferative potency should therefore be considered a trend within the investigated series rather than a generalizable SAR rule. Broader conclusions regarding the contribution of this structural arrangement to biological potency would require the evaluation of a more structurally diverse compound library and systematic modification of the relevant molecular features.

Despite these promising immunomodulatory observations, the reliance on the human THP-1 cell line as an in vitro macrophage model, although valuable for standardizing pharmacological investigations, represents a reductionist approach. THP-1-derived macrophages cannot fully capture the profound spatial organization, cellular heterogeneity, metabolic and cytokine milieu, and complex intercellular interactions characteristic of tumor-associated macrophages (TAMs) within the native tumor microenvironment. Furthermore, the phenotype and functional responses of THP-1-derived macrophages may differ from those of primary human macrophages and TAMs in vivo. Therefore, the effects observed in the present study should be interpreted within the context of this simplified experimental model. Consequently, while these findings establish *N*-CIMZ **1** as a promising compound in the presented in vitro conditions, further studies using primary human macrophages, 3D co-culture models, and appropriate in vivo tumor models will be necessary to determine whether the immunomodulatory properties observed for *N*-CIMZ **1** are maintained in a complex tumor microenvironment and to further evaluate its translational potential.

## 4. Materials and Methods

### 4.1. Chemistry

#### 4.1.1. General

All chemicals and solvents were supplied by Fisher Scientific (Montreal, QC, Canada), and were used as received unless specified otherwise. Proton and carbon NMR spectra were recorded on a Bruker Avance-300 spectrometer (Bruker, Karlsruhe, Germany), with chemical shifts (δ) reported in parts per million (ppm). Melting points were measured using an MPA100 automated melting point system (Stanford Research Systems, Sunnyvale, CA, USA) and are reported uncorrected.

Liquid flash chromatography was performed on RediSep^®^ Silver silica gel disposable flash columns with silica gel F60 (60 Å, 40–63 μm, Teledyne Labs, Lincoln, NE, USA) using the Combiflash EZ PREP purification system (Teledyne Labs, NE, USA) with solvent mixtures expressed as *v*/*v* ratios. Solvents and reagents were used without prior purification unless otherwise noted. Reaction progress was monitored by TLC using precoated 60 F254 silica gel plates (Macherey-Nagel, Düren, Germany) and chromatograms were visualized under UV light at 254 and/or 325 nm. UHPLC analyses were performed on an ACQUITY Arc system (Waters, Mississauga, ON, Canada) equipped with a 2998 UV-visible PDA detector. Samples were eluted using a mixture of H_2_O/MeOH/DMSO (49.5/49.5/1) with a linear mobile phase gradient (1.0 mL/min) on a CORTECS C18+ silica-based reversed-phase column (90 Å, 3.0 mm × 50 mm × 2.7 µm) mounted on a CORTECS C18+ VanGuard Cartridge (90 Å, 2.1 mm × 5 mm × 2.7 µm, Waters, Mississauga, ON, Canada). Detection was performed at optimal wavelengths for maximum absorption and all compounds were obtained at a purity meeting or exceeding 95%. To ensure accurate purity assessments of chromatographic purity, solvent blanks were analyzed under identical chromatographic conditions. Peaks attributable to the injection solvent, mobile phase, or known system/column-related background were identified and excluded from the purity calculation. Peaks detected in the sample chromatograms were included in the integration unless they could be unambiguously assigned to these background sources. The reported purity therefore represents the percentage of integrated chromatographic peak area attributable to the compound under the applied UHPLC-UV conditions. High-resolution mass spectrometry (HRMS) spectra were obtained by direct injection into a TOF system 6210 series mass spectrometer (Agilent Technologies, Santa Clara, CA, USA). Compound **11** was previously described by Moreau et al. [56]. Compound **12** was previously described by Johnston et al. [57]. The cyclization to yield derivatives 13 and 14 was previously described by Chavez-Alvarez et al. [58].

#### 4.1.2. General Preparation of *N*-CIMZs **1**–**8**

The synthesis of *N*-CIMZs **1**–**8** began with the addition of 1.0 equivalent (eq.) of 1-(3-aminophenyl)ethenone (**9**) or 1-(4-aminophenyl)ethenone (**10**) to 30 mL of anhydrous methylene chloride, followed by the addition of 1.0 eq. of 2-chloroethyl isocyanate. The reaction was stirred at room temperature overnight, affording intermediates 1-(3-acetylphenyl)-3-(2-chloroethyl)urea (**11**) or 1-(4-acetylphenyl)-3-(2-chloroethyl)urea (**12**), respectively, which precipitated upon addition of cold hexane, and were isolated by filtration (yield: 80–85%). Subsequently, 1.0 eq. of **11** or **12** was dissolved in 15 mL of tetrahydrofuran, and reacted with 1.2 eq. of sodium methanolate under reflux (60 °C) for 24 h. The reaction mixture was extracted with methylene chloride (3 × 15 mL). The combined organic layers were washed with brine (1 × 45 mL), dried over MgSO_4_, filtered and evaporated under reduced pressure. The obtained crude products were purified by flash chromatography on silica gel to afford either 1-(4-acetylphenyl)imidazolidin-2-one (**13**) or 1-(3-acetylphenyl)imidazolidin-2-one (**14**) (yield: 35–40%). Finally, 1.0 eq. of **13** or **14** was dissolved in 10 mL of methanol and treated with 4.0 eq. of sodium hydroxide (10% (*w*/*v*), approximately 2.5M). After homogenization under magnetic stirring, 1.0 eq. of the corresponding substituted aldehyde was added, and the reaction was heated at reflux overnight. After cooling to room temperature, the resulting *N*-CIMZs **1**–**8** were obtained by recrystallization from warm MeOH (10 mL). Afterwards, the solid products were cooled to 0 °C, filtered, and purified by flash chromatography on silica gel to afford the final *N*-CIMZs **1**–**8**.

#### 4.1.3. Characterization of *N*-CIMZs **1**–**8**

(E)-1-(3-(3-(3,4,5-Trimethoxyphenyl)acryloyl)phenyl)imidazolidin-2-one (**1**). Flash chromatography (methylene chloride to methylene chloride/ethyl acetate (75/25, *v*/*v*)). Yield: 50%; light yellow solid; mp: 198–200 °C; ^1^H NMR (CDCl_3_): δ 7.97–7.95 (m, 2H, Ar), 7.73–7.65 (m, 2H, Ar and CH=CH), 7.45 (dd, *J* = 8.9, 7.6 Hz, 1H), 7.37 (d, 1H, *J* = 15.5 Hz, CH=CH), 6.86 (s, 2H, Ar), 4.03–3.97 (m, 2H, CH_2_), 3.90 (s, 6H, 2x OCH_3_), 3.88 (s, 3H, OCH_3_), 3.62–3.57 (m, 2H, CH_2_); ^13^C NMR (CDCl_3_): δ 190.6, 159.7, 153.5, 145.3, 140.6, 138.8, 130.4, 129.1, 122.8, 122.4, 121.6, 117.0, 116.6, 105.8, 61.0, 56.3, 45.3, 37.4; HRMS-(ESI) *m*/*z,* calculated for [C_21_H_23_N_2_O_5_]^+^ ([M + H]^+^): 383.1601; found: 383.1596.

(E)-1-(3-(3-(2,6-Dichlorophenyl)acryloyl)phenyl)imidazolidin-2-one (**2**). Flash chromatography (methylene chloride to methylene chloride/ethyl acetate (75/25, *v*/*v*)). Yield: 50%; yellow solid; mp: 197–199 °C; ^1^H NMR (CDCl_3_ + DMSO-d_6_): δ 7.74–7.73 (m, 1H, Ar), 7.54–7.50 (m, 1H, Ar), 7.43–7.37 (m, 1H, CH=CH), 7.31–7.22 (m, 2H, Ar and CH=CH), 7.10–6.99 (m, 3H, Ar), 6.90–6.78 (m, 1H, Ar), 3.61–3.55 (m, 2H, CH_2_), 3.20–3.15 (m, 2H, CH_2_);^13^C NMR (CDCl_3_ + DMSO-d_6_): δ 192.4, 189.5, 159.2, 141.0, 137.6, 137.2, 134.7, 132.1, 130.3, 129.9, 128.8, 128.7, 121.9, 116.6, 44.7, 36.9. HRMS-(ESI), *m*/*z* calculated for [C_18_H_15_Cl_2_N_2_O_2_]^+^ ([M + H]^+^): 361.0505; found: 361.0497. Two resonances are observed for the carbonyl carbon of the chalcone moiety, at δ 189.5 and 192.4 ppm. This observation could be related to the coexistence of two conformational species in solution, without, however, allowing us to confirm, on the sole basis of the available NMR data, that these correspond to two distinct rotamers.

(E)-1-(3-(3-(2,5-Dimethylphenyl)acryloyl)phenyl)imidazolidin-2-one (**3**). Flash chromatography (methylene chloride to methylene chloride/ethyl acetate (75/25, *v*/*v*)). Yield: 35%; bright yellow solid; mp: 153–155 °C; ^1^H NMR (DMSO-d_6_): δ 8.18–8.16 (m, 1H, Ar), 7.97 (d, *J* = 15.5 Hz 1H, CH=CH), 7.91 (dd, *J* = 8.0, 2.3 Hz, 1H), 7.83–7.80 (m, 2H, Ar), 7.73 (d, *J* = 15.5 Hz 1H, CH=CH), 7.52 (t, *J* = 8.0 Hz, 1H, Ar), 7.19 (s, 2H), 7.12 (brs, 1H, NH), 3.98–3.93 (m, 2H, CH_2_), 3.45 (t, *J* = 8.0 Hz, 2H, CH_2_), 2.41 (s, 3H, CH_3_), 2.35 (s, 3H, CH_3_); ^13^C NMR (DMSO-d_6_): δ 189.5, 183.1, 159.0, 141.3, 138.1, 135.5, 135.1, 133.0, 131.3, 130.8, 129.0, 127.2, 122.9, 121.9, 121.5, 116.2, 44.5, 36.6, 20.6, 18.9; HRMS-(ESI) *m*/*z*, calculated for [C_20_H_21_N_2_O_2_]^+^ ([M + H]^+^): 321.1598; found: 321.1592. Two resonances are observed for the carbonyl carbon of the chalcone moiety, at δ 189.5 and 183.1 ppm. This observation could be related to the coexistence of two conformational species in solution, without, however, allowing us to confirm, on the sole basis of the available NMR data, that these correspond to two distinct rotamers.

(E)-1-(3-(3-(2,6-Difluorophenyl)acryloyl)phenyl)imidazolidin-2-one (**4**). Flash chromatography (methylene chloride to methylene chloride/ethyl acetate (75/25, *v*/*v*)). Yield: 50%; orange solid; mp: 193–195 °C; ^1^H NMR (DMSO-d_6_): δ 8.27–8.26 (m, 1H, Ar), 7.89–7.83 (m, 2H, Ar and CH=CH), 7.74–7.52 (m, 4H, Ar and CH=CH), 7.30 (m, 2H, Ar), 7.13 (brs, 1H, NH), 3.98–3.92 (m, 2H, CH_2_), 3.46 (m, 2H, CH_2_);^13^C NMR (DMSO-d_6_): δ 189.3, 183.1, 159.5, 158.9, 141.4, 137.5, 132.7, 129.3, 127.8, 121.6, 116.3, 112.7, 112.3, 105.2, 81.0, 44.4, 36.6; HRMS-(ESI) *m*/*z*, calculated for [C_18_H_15_F_2_N_2_O_2_]^+^ ([M + H]^+^): 329.1096; found: 329.1104. Two resonances are observed for the carbonyl carbon of the chalcone moiety, at δ 189.3 and 183.1 ppm. This observation could be related to the coexistence of two conformational species in solution, without, however, allowing us to confirm, on the sole basis of the available NMR data, that these correspond to two distinct rotamers.

(E)-1-(4-(3-(3,4,5-Trimethoxyphenyl)acryloyl)phenyl)imidazolidin-2-one (**5**). Flash chromatography (methylene chloride to methylene chloride/ethyl acetate (75/25, *v*/*v*)). Yield: 70%; light yellow solid; mp: 218–221 °C; ^1^H NMR (DMSO-d_6_): δ 8.18–8.16 (m, 2H, Ar), 7.92 (d, J = 15.6 Hz, 1H, CH=CH), 7.83–7.55 (m, 3H, Ar and CH=CH), 7.23 (s, 2H. Ar), 6.53 (s, 1H, NH), 3.98–3.88 (m, 2H, CH_2_), 3.88 (s, 6H, 2x OCH_3_), 3.72 (s, 3H, OCH_3_), 3.46–3.43 (m, 2H, CH_2_); ^13^C NMR (DMSO-d_6_): δ 185.1, 158.5, 155.8, 153.2, 145.1, 143.5, 130.5, 129.8, 121.3, 116.0, 113.1, 106.5, 60.2, 56.2, 44.3, 36.4; HRMS-(ESI) *m*/*z*, calculated for [C_21_H_23_N_2_O_5_]^+^ ([M + H]^+^): 383.1601; found: 383.1597.

(E)-1-(4-(3-(2,6-Dichlorophenyl)acryloyl)phenyl)imidazolidin-2-one (**6**). Flash chromatography (methylene chloride to methylene chloride/ethyl acetate (75/25, *v*/*v*)). Yield: 50%; light yellow solid; mp: 238–239 °C; ^1^H NMR (CDCl_3_ + DMSO-d_6_): δ 7.42–7.39 (m, 2H, Ar), 7.20–7.08 (m, 4H, Ar and 2 x CH=CH), 6.87–6.84 (m, 2H, Ar), 6.70 (dd, *J* = 8.9, 7.2 Hz, 1H, Ar), 3.61 (brs, 1H, NH), 3.41–3.35 (m, 2H, CH_2_), 2.99–2.93 (m, 2H, CH_2_); ^13^C NMR (CDCl_3_ + DMSO-d_6_): δ 191.1, 187.4, 158.4, 144.9, 136.0, 134.4, 132.1, 130.0, 129.8, 129.4, 128.6, 116.0, 44.3, 36.5; HRMS-(ESI) *m*/*z*, calculated for [C_18_H_15_Cl_2_N_2_O_2_]^+^ ([M^+^ + H]^+^): 361.0505; found: 361.0493. Two resonances are observed for the carbonyl carbon of the chalcone moiety, at δ 187.4 and 191.1 ppm. This observation could be related to the coexistence of two conformational species in solution, without, however, allowing us to confirm, on the sole basis of the available NMR data, that these correspond to two distinct rotamers.

(E)-1-(4-(3-(2,5-Dimethylphenyl)acryloyl)phenyl)imidazolidin-2-one (**7**). Flash chromatography (methylene chloride to methylene chloride/ethyl acetate (75/25, *v*/*v*)). Yield: 30%; light yellow solid; mp: 209–211 °C; ^1^H NMR (DMSO-d_6_): δ 8.18–8.15 (m, 2H, Ar), 7.98–7.93 (d, *J* = 15.5 Hz, 1H, CH=CH), 7.86–7.74 (m, 4H, Ar and CH=CH), 7.29 (brs, 1H, NH), 7.18 (s, 2H, Ar), 3.98–3.93 (m, 2H, CH_2_), 3.46 (m, 2H, CH_2_), 2.40 (s, 3H, CH_3_), 2.35 (s, 3H, CH_3_); ^13^C NMR (DMSO-d_6_): δ 187.4, 158.5, 145.1, 140.3, 135.4, 134.9, 133.2, 131.0, 130.7, 130.5, 129.8, 127.2, 122.6, 116.0, 44.3, 36.4, 20.6, 18.9; HRMS-(ESI) *m*/*z*, calculated for [C_20_H_21_N_2_O_2_]^+^ ([M + H]^+^): 321.1598; found: 321.1594.

(E)-1-(4-(3-(2,6-Difluorophenyl)acryloyl)phenyl)imidazolidin-2-one (**8**). Flash chromatography (methylene chloride to methylene chloride/ethyl acetate (75/25, *v*/*v*)). Yield: 65%; light orange solid; mp: 230–232 °C; ^1^H NMR (DMSO-d_6_): δ 8.06–8.02 (m, 2H, Ar), 7.91–7.86 (m, 1H, CH=CH,), 7.78–7.65 (m, 3H, Ar and CH=CH), 7.61–7.51 (m, 1H, Ar), 7.32–7.23 (m, 3H, Ar and NH), 3.94 (t, *J* = 7.8 Hz, 2H, CH_2_), 3.47 (t, *J* = 7.8 Hz, 2H, CH_2_);^13^C NMR (DMSO-d_6_): δ 187.2, 183.1, 162.8, 162.7, 159.5, 159.4, 158.4, 145.4, 132.5, 132.3, 132.2, 129.9, 129.7, 129.5, 128.4, 127.6, 127.5, 127.4, 116.2, 112.6, 112.4, 112.3, 112.2, 112.0, 44.3, 36.4; HRMS-(ESI) *m*/*z*, calculated for [C_18_H_15_F_2_N_2_O_2_]^+^ ([M + H]^+^): 329.1096; found: 329.1093. Two resonances are observed for the carbonyl carbon of the chalcone moiety, at δ 187.2 and 183.1 ppm. This observation could be related to the coexistence of two conformational species in solution, without, however, allowing us to confirm, on the sole basis of the available NMR data, that these correspond to two distinct rotamers.

### 4.2. Biology

#### 4.2.1. Cell Culture

All the cell lines were obtained from the American Type Culture Collection (ATCC). MDA-MB-231 and MDA-MB-453 cells were cultured in Dulbecco’s Modified Eagle Medium (DMEM, Gibco, Thermo Fisher Scientific, Strasbourg, France) and BT-20 and MCF-7 cells were maintained in Eagle’s Minimum Essential Medium (EMEM, Gibco, Thermo Fisher Scientific, Strasbourg, France). All the cell lines were supplemented with 10% fetal bovine serum (FBS, Dutscher, Bernolsheim, France) and 1% penicillin–streptomycin (Thermo Fisher Scientific, Strasbourg, France). MCF-7 cells were additionally supplemented with 0.01 mg/mL recombinant human insulin (Gibco, Thermo Fisher Scientific, Strasbourg, France). All the cell lines were grown at 37 °C in a moisture-saturated atmosphere containing 5% CO_2_.

#### 4.2.2. Antiproliferative Activity Assay

The antiproliferative activity of *N*-CIMZs **1**–**8** was assessed using the sulforhodamine B assay following the National Cancer Institute (NCI) protocol [59] with minor modifications. Briefly, 96-well Nunc clear plates (Thermo Fisher Scientific, Strasbourg, France) were seeded with 75 µL of cell suspension of MCF-7, BT-20 and MDA-MB-453 at 3.5 × 10^3^ and MDA-MB-231 at 3.0 × 10^3^ cells/well in their respective culture media. After 24 h at 37 °C, cells were treated with freshly solubilized test compounds in dimethyl sulfoxide (DMSO) at their maximum solubility and diluted in 75 µL of culture medium using serial dilutions. DMSO concentration was kept constant at 0.2% (*v*/*v*). Cells were incubated with the tested compounds for 48 h. Following the treatment, plates were washed with phosphate-buffered saline (PBS, Thermo Fisher Scientific, Strasbourg, France), and cells were fixed with cold trichloroacetic acid 10% (*w*/*v*) (TCA, Thermo Fisher Scientific, Strasbourg, France), and they were incubated for 2 h at 4 °C. Plates were emptied, rinsed thrice with distilled water and air-dried at room temperature, and subsequently stained with 75 µL of sulforhodamine B (SRB, Thermo Fisher Scientific, Strasbourg, France) solution 0.1% (*w*/*v*) in 1% acetic acid for 15 min. Excess dye was discarded, and the plates were washed thrice with 1% acetic acid and air-dried. Cell-bound dye was solubilized with 200 µL of a 20 mM Tris base (Thermo Fisher Scientific, Strasbourg, France), and plates were incubated for 2 h. Absorbance was measured at an optimal wavelength (between 530 and 570 nm) using a Multiskan GO microplate spectrometer (Thermo Fisher Scientific, Strasbourg, France). Antiproliferative activity values (IC_50_) and standard deviation were calculated with GraphPad Prism 10.4.0 software (GraphPad Software, San Jose, CA, USA). Experiments were performed in triplicate and repeated at least three times. Results are presented as the mean ± standard deviation of independent experiments.

#### 4.2.3. Scratch-Wound Healing Assay

MCF-7 cells were seeded at 1 × 10^4^ cells/well on 96-well plates (Thermo Fisher Scientific, Strasbourg, France) and incubated at 37 °C for 24 h. A scratch was introduced into the cell monolayer using an IncuCyte^®^ Scratch Wound Assay (Sartorius, Dourdan, France) to perform a wound healing assay according to the supplier instructions, allowing the monitoring of MCF-7 gap closure over time using an Incucyte^®^ S3 (Sartorius, Dourdan, France). Cells were then treated with *N*-CIMZ **1** at 2.5 µM, CA-4 at 125 nM, and DMSO at 0.2% and a control group of untreated cells was used for comparison. Measures were acquired automatically at a frequency of one acquisition per hour during 36 h. The Incucyte^®^ S3 software provided quantitative readouts (confluence and morphology metrics). The data is expressed as the mean of three separate experiments and the error is expressed as the standard deviation of independent experiments. The detailed statistical analysis of MCF-7 scratch-wound closure dynamics including the Two-Way Repeated Measures (RM) ANOVA and Tukey’s multiple comparisons test is provided in Table S1 of the supporting information file.

#### 4.2.4. Cell Cycle Progression Analysis

Cell cycle analysis was conducted by seeding MCF-7 cells at 4.0 × 10^5^ cells/well in 6-well plates (Thermo Fisher Scientific, Strasbourg, France) and they were maintained in the respective media at 37 °C for 24 h. The cells were then treated with *N*-CIMZ **1** (2.5 µM), CA-4 (125 nM) or DMSO (0.2%, negative control). CA-4 was used as the positive control. After 24 h, the cells were trypsinized, rinsed with PBS and resuspended in 250 µL of PBS. Fixation of the cells was achieved by the gradual addition of 750 µL of cold ethanol, and the samples were stored at −20 °C until analysis. On the day of the analysis, cells were washed with PBS and stained with a solution of 4′,6′-diamidino-2-phenylindole (DAPI, Sigma-Aldrich, St. Louis, MO, USA) at 2 µg/mL in PBS. The cell cycle progression was assessed using a MACSQuant^®^ Analyzer 16 Flow Cytometer (Miltenyi, Paris, France) and the data were analyzed using MACSQuantify™ Software V3.0.2 (Miltenyi, Paris, France). The experiments were performed three times independently, and results are expressed as mean ± standard deviation of independent experiments.

#### 4.2.5. Immunocytochemistry for the Analysis of the Effect on the Microtubule Network

MCF-7 cells were seeded at a density of 3 × 10^4^ cells/well in 24-well plates containing glass coverslips and allowed to attach for 24 h. The cells were then treated for 24 h with *N*-CIMZ **1** (2.5 µM), the reference compound CA-4 (125 nM), vehicle (DMSO 0.2%), or left untreated. Following treatment, cells were washed twice with cold PBS and fixed with 4% paraformaldehyde (PFA) for 15 min at room temperature. After three washes with PBS, cells were permeabilized with 0.1% Triton X-100 in PBS for 10 min at room temperature. The cells were washed three times with PBS and blocked with 3% Bovine Serum Albumin (BSA) for 45 min at room temperature. For microtubule staining, cells were incubated for 2 h at room temperature with a mouse monoclonal anti-β-tubulin primary antibody (clone TBN06 Tub 2.5, Cat. #MA5-11732, Invitrogen, Thermo Fisher Scientific, Strasbourg, France) at a 1:200 dilution. Following three washes with PBS, cells were incubated for 1 h at room temperature with a Goat anti-Mouse IgG (H+L) Cross-Adsorbed Secondary Antibody, Alexa Fluor 488 (Cat. #A11001, Invitrogen, Thermo Fisher Scientific, Strasbourg, France) at a 1:1000 dilution. Both primary and secondary antibodies were diluted in a buffer containing PBS, 1% BSA, and 0.05% Triton X-100. The cells were washed three times with PBS and nuclei were counterstained with DAPI (4′,6-Diamidino-2-phenylindole dihydrochloride, Merck KGaA, Darmstadt, Germany, 2 µg/mL). After three final PBS washes, the coverslips were mounted onto Superfrost microscope slides using VECTASHIELD^®^ Antifade Mounting Medium (Eurobio Scientific, Courtaboeuf, France). Images were acquired using a Leica TCS SP8 LIGHTNING confocal microscope equipped with a resonant scanner and Navigator software (LAS X version 3.5.5, Leica Microsystems, Wetzlar, Germany). Subsequent image processing and visualization were performed using ImageJ 1.54p software (National Institutes of Health, Bethesda, MD, USA). The photographs shown are representative of three independent replicates.

#### 4.2.6. Flow Cytometry Analysis of CD14, CD86, CD206, CD209, CD163, LAP (TGF-β1), and Intracellular Inos Expression

To quantify phenotypic shifting and macrophage subpopulation dynamics, the expression profiles of differentiation markers, polarization surface scaffolds, and metabolic enzymes were evaluated via multi-parametric flow cytometry in human THP-1-derived macrophages.

##### Cellular Differentiation and Priming (M0 Baseline)

Human THP-1 monocytes were seeded at a density of 4.0 × 10^5^ cells/well in standard 6-well culture plates (Thermo Fisher Scientific, Strasbourg, France) and incubated at 37 °C with 5% CO_2_ for 24 h. Macrophage differentiation into the resting M0 baseline state was induced by treating the cultures with 100 nM phorbol 12-myristate 13-acetate (PMA; Tocris Bioscience, Bristol, UK) for 24 h. Following differentiation, the PMA-conditioned media was aspirated, the adherent monolayers were washed once with sterile phosphate-buffered saline (PBS), and fresh, stimulus-free RPMI complete medium was replenished. Cells were allowed to adjust under standard incubation settings for an additional 24 h recovery window prior to experimental conditioning.

##### Activation Regimens and Compound Co-Treatment

Following the recovery period, the differentiated M0 macrophage layers were subjected to specific polarization activation regimens for 24 h, either in the presence or absence of *N*-CIMZ **1**. For classical M1 Activation: Monolayers were challenged with a combination of lipopolysaccharide (LPS; 100 ng/mL) and recombinant human interferon-gamma (IFN-γ; 20 ng/mL) to drive classical pro-inflammatory polarization. For the alternative M2 activation THP-1-derived M0 macrophages were exposed to a cytokine cocktail comprising recombinant human interleukin-4 (IL-4; 20 ng/mL) and interleukin-13 (IL-13; 20 ng/mL) to drive alternative tumor-supportive polarization. To evaluate immunomodulatory interception, *N*-CIMZ **1** (2.5 µM) was introduced simultaneously alongside either the M1 or M2 polarizing cocktails. Parallel wells receiving standard medium served as the untreated baseline control, while wells treated with DMSO 0.2% were utilized as the vehicle control cohort. All groups excluding the untreated control contained the matched 0.2% DMSO vehicle.

##### Harvesting and Sample Immunostaining

At the 24 h endpoint, the supernatant was removed, and the cells were detached mechanically by gentle scraping in cold PBS. The resulting cellular suspensions were centrifuged and washed with PBS. To analyze surface and intracellular markers within the same multiplexed experimental assay, a sequential staining protocol was performed.

To prevent non-specific antibody binding via surface Fc receptors and eliminate cellular aggregation, cell pellets were first resuspended in a solution containing 10% human FcR Blocking Reagent (Miltenyi, Paris, France) in PBS supplemented with 0.5% bovine serum albumin (BSA; Thermo Fisher Scientific, Strasbourg, France) and 2 mM EDTA (Thermo Fisher Scientific, Strasbourg, France) which constitutes the staining buffer. The suspensions were incubated for 20 min at 4 °C prior to any antibody exposure. Without washing out the blocking matrix, cell pellets were first resuspended in staining buffer and incubated in the dark at 4 °C for 30 min with fluorochrome-conjugated monoclonal antibodies targeting the cell surface antigens CD14 (BD Pharmingen™ APC-Cy™7 mouse anti-human, CD86 (anti-human, FITC, Miltenyi, Paris, France), CD206 (anti-human, PerCP-Vio^®^ 700, Miltenyi, Paris, France), CD209 (BD Pharmingen™ APC mouse anti-human, BD Biosciences, Le Pont-de-Claix, France), CD163 (anti-human, VioBlue^®^, REAfinity™, Miltenyi, Paris, France) and LAP (TGF-β1) (anti-human, APC, REAfinity™, Miltenyi, Paris, France) as well as the structural membrane integrity dye Viobility™ 405/520 Fixable Dye (Miltenyi, Paris, France) diluted and incubated according to the manufacturer’s instructions. Following a PBS wash to remove unbound surface antibodies, cells were fixed with 4% paraformaldehyde (PFA) and subsequently permeabilized using 0.1% Triton X-100 in PBS for 10 min at room temperature to allow intracellular access. To reduce non-specific background noise, cells were incubated with 1% bovine serum albumin (BSA) in PBS for 20 min. Finally, the intracellular target was resolved by incubating the permeabilized matrix with the anti-iNOS antibody (anti-human, PE, REAfinity™, Miltenyi, Paris, France). Following the final staining step, the multi-labeled populations were thoroughly washed with PBS, resuspended in flow cytometry buffer, and immediately analyzed. A minimum of 10,000 gating events were recorded per independent experimental replicate (*n* = 3, independent biological experiments performed on different days). Cellular debris and doublets were excluded based on forward and side scatter properties (FSC/SSC). Subsequently, cells with structural membrane damage were strictly excluded from the analysis based on the Viobility™ 405/520 Fixable Dye signal. Positivity thresholds for all surface and intracellular markers were rigorously established using Fluorescence Minus One (FMO) controls to account for spectral overlap and non-specific background fluorescence. Fluorescence compensation was established using single-stained controls to calculate the compensation matrix and correct for spectral spillover prior to data analysis. To correct for spectral overlap between adjacent fluorochromes, fluorescence compensation was established prior to data acquisition. Single-stained control samples (using untreated representative cells) were prepared for each specific fluorophore. These single-color controls were acquired on the MACSQuant^®^ Analyzer 16 Flow Cytometer (Miltenyi, Paris, France) and the spillover compensation matrix was automatically calculated and applied using the instrument’s built-in automated compensation tool within the MACSQuantify™ Software V3.0.2 (Miltenyi, Paris, France) to ensure accurate resolution of the fluorescent populations. Quantification is presented as the mean ± the standard deviation of independent experiments. The percentage of unstained cells for the Viobility™ 405/520 Fixable Dye across all experimental conditions is provided in Supplementary Figure S1.

#### 4.2.7. Statistical Analysis

All statistical analyses were performed using GraphPad Prism 10.4.0 software (GraphPad Software, San Jose, CA, USA). Data are presented as the mean ± standard deviation (SD) of at least three independent biological experiments (*n* ≥ 3). For general endpoint assays, statistical significance between experimental groups and their respective controls was determined using a One-Way Analysis of Variance (ANOVA) followed by Dunnett’s post-hoc multiple comparisons test. For the in vitro scratch-wound closure time-course, statistical significance was evaluated using a Two-Way Repeated Measures (RM) ANOVA followed by Tukey’s post-hoc multiple comparisons test to accurately account for the treatment-by-time interaction. A p-value of less than 0.05 was considered statistically significant (* *p* < 0.05, ** *p* < 0.01, *** *p* < 0.001, **** *p* < 0.0001).

## 5. Conclusions

This study demonstrates the successful synthesis and pharmacological evaluation of a novel series of *N*-chalconyl imidazolidones (**1**–**8**), establishing a promising dual-action chemical scaffold that combines direct antimitotic efficacy with macrophage-modulating properties. Comprehensive structure–activity relationship (SAR) analysis revealed that the biological potency of these derivatives is heavily dictated by precise geometric and electronic features, prioritizing *N*-CIMZ **1**, which is characterized by a favorable 1,3-disubstitution pattern on ring A and a 3,4,5-trimethoxy motif on ring B as the prototype candidate across a molecularly diverse panel of human breast cancer cell lines.

Mechanistically, *N*-CIMZ **1** operates as a high-efficiency cytostatic agent, replicating the antimitotic footprint of the reference standard CA-4. The compound drives a robust, targeted cell cycle accumulation at the G2/M phase in MCF-7 cells. This late-stage checkpoint delay translates functionally into a persistent, time-dependent restriction of collective cell motility, successfully arresting scratch-wound closure. Furthermore, confocal immunofluorescence validation suggests that this antimitotic efficacy is driven by the disruption of the cellular microtubule architecture, establishing *N*-CIMZ **1** as a promising antimitotic hybrid candidate for advanced oncological interventions.

The therapeutic value of *N*-CIMZ **1** extends beyond traditional direct cytotoxicity by exercising sophisticated regulatory control over immune cell plasticity. In human THP-1 macrophages, the prototype compound exhibits a reduced genotoxic profile, maintaining low baseline γH2AX levels. Under tumor-supportive alternative M2 pressure, *N*-CIMZ **1** successfully intercepts polarization pathways, forcing high membrane retention of the immunosuppressive cytokine carrier LAP (TGF-β1) and cleanly suppressing the key pro-tumorigenic scavenger receptor CD163. Simultaneously, it acts as a homeostatic buffer under hyper-reactive pro-inflammatory M1 stress, blunting volatile shifts and reducing the emergence of ambiguous, intermediate CD206^+^/CD209^+^ hybrid subpopulations.

Taken together, these multi-parametric findings validate the dual-front pharmacological paradigm of *N*-CIMZ **1**. By simultaneously inhibiting in vitro scratch-wound closure and shifting macrophage surface-marker distributions, this innovative *N*-chalconyl imidazolidone scaffold stands out as a highly compelling candidate for the development of multi-modal combinatory therapies designed to modulate selected macrophage surface markers and inhibit cell proliferation in aggressive breast malignancies.

## Figures and Tables

**Figure 1:**
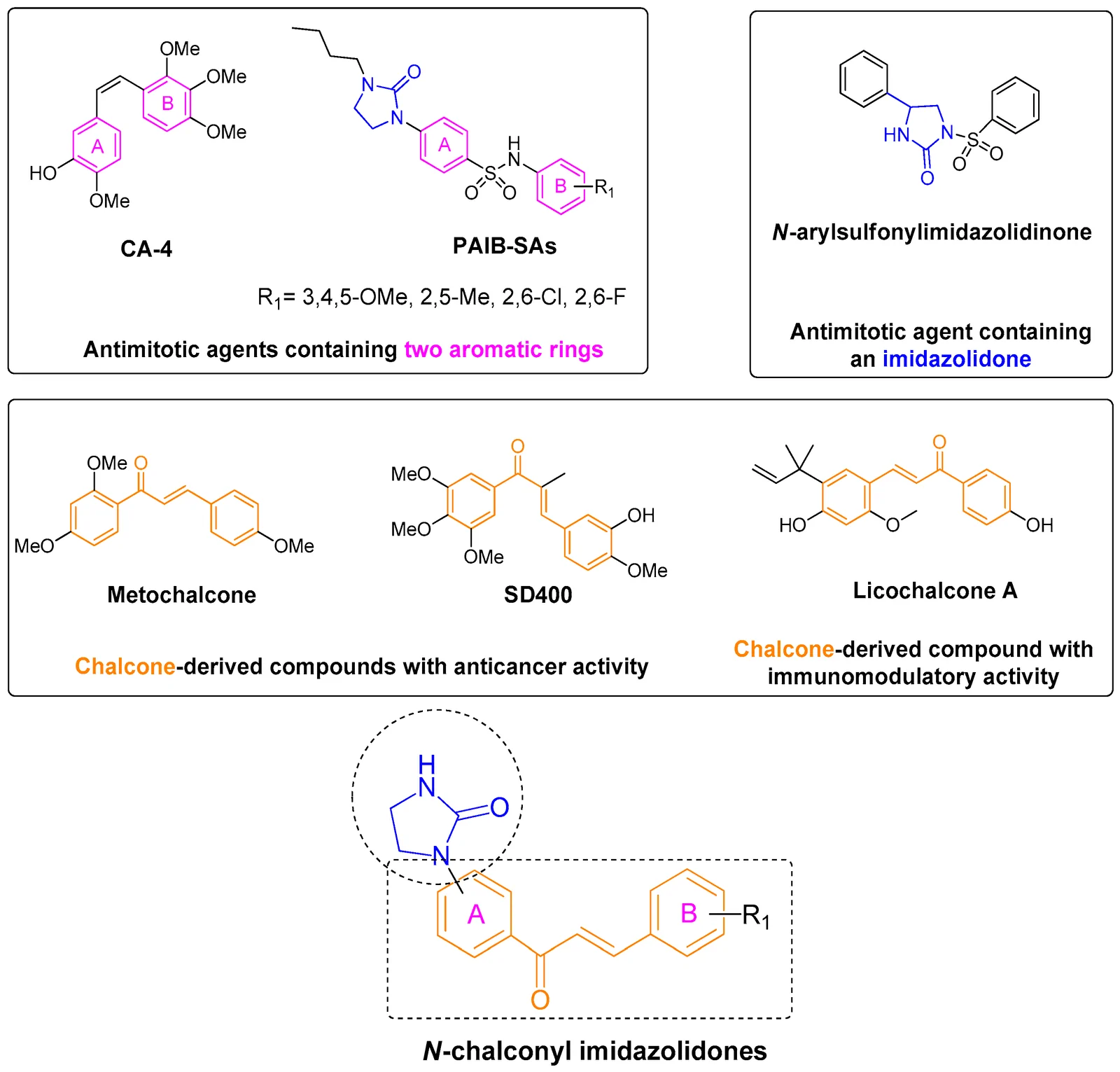
Molecular structure of *N*-chalconyl imidazolidones and related compounds CA-4, PAIB-SAs, *N*-arylsulfonylimidazolidinone, metochalcone, SD400 and licochalcone A.

**Figure 2:**
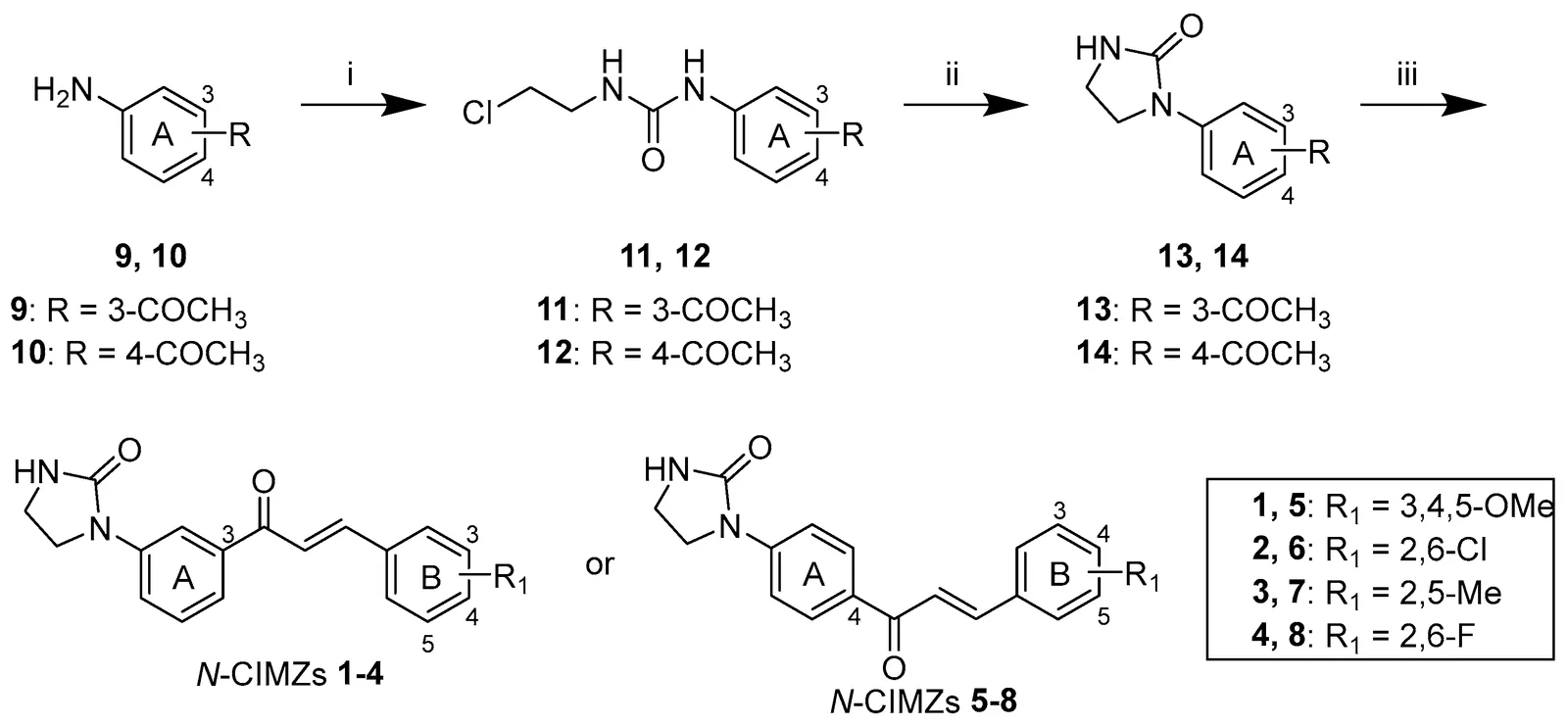
General synthesis of *N*-chalconyl imidazolidones (*N*-CIMZs, **1**–**8**). Reagents and conditions: (i) 2-chloroethyl isocyanate, DCM, room temperature, overnight; (ii) sodium methanolate, THF, reflux, overnight; (iii) NaOH, MeOH and the relevant aldehyde, reflux, overnight.

**Figure 3:**
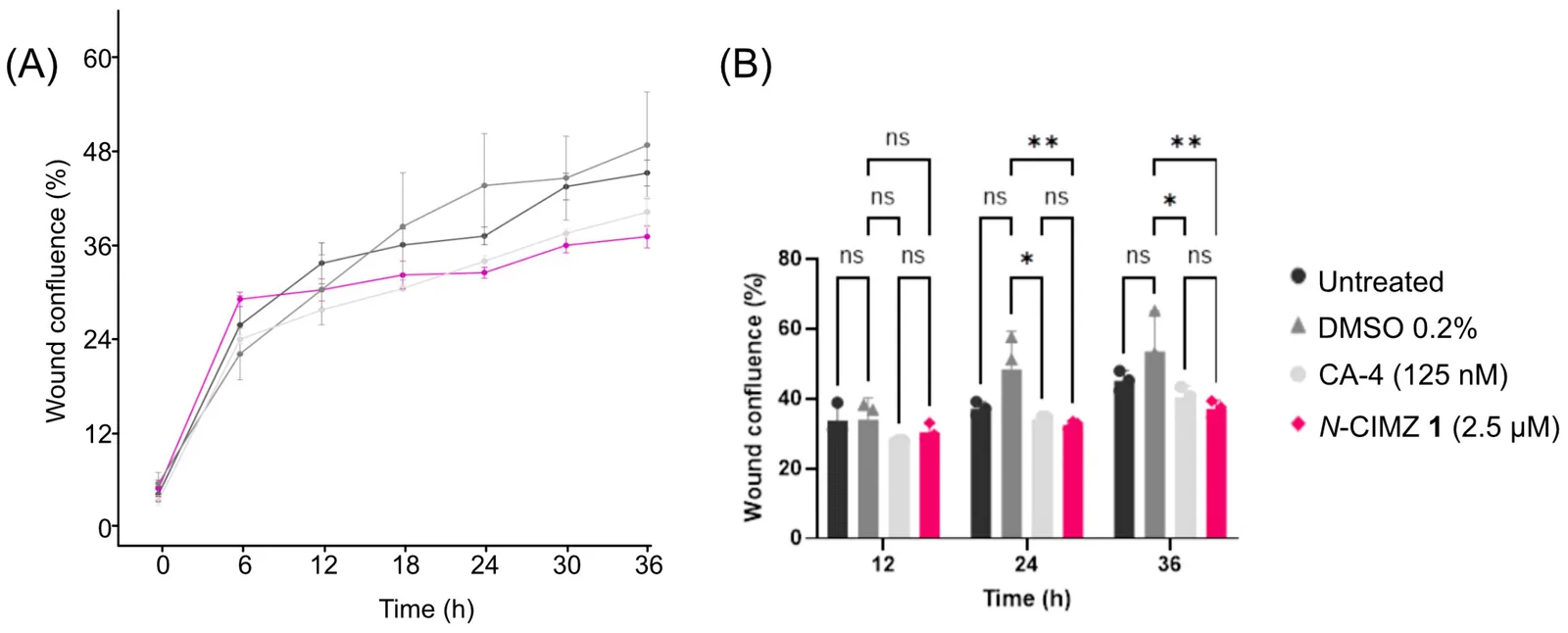
Effect of *N*-CIMZ **1** at 2.5 µM and CA-4 at 125 nM on MCF-7 cell wound closure assessed by a wound-healing assay. (**A**) Total wound confluence (expressed as percentage area confluence) over a 36-h period for untreated, DMSO 0.2% vehicle control, CA-4 (125 nM) and *N*-CIMZ **1** (2.5 µM) groups. Data represent the mean ± standard deviation (SD) of three independent biological replicates (*n* = 3), with error bars shown. (**B**) Quantitative grouped bar histogram of wound confluence (percentage) at selected time points (12 h, 24 h, and 36 h). Individual biological replicate data points are superimposed as colored icons corresponding to the legend. Statistical significance was determined compared to the DMSO 0.2% vehicle control group using a Two-Way Repeated Measures (RM) ANOVA followed by Tukey’s multiple comparisons test; p-values are denoted as * *p* < 0.05, ** *p* < 0.01, ns = non-significant. Experimental conditions containing *N*-CIMZ **1** or CA-4 contained a final concentration of 0.2% DMSO (vehicle control).

**Figure 4:**
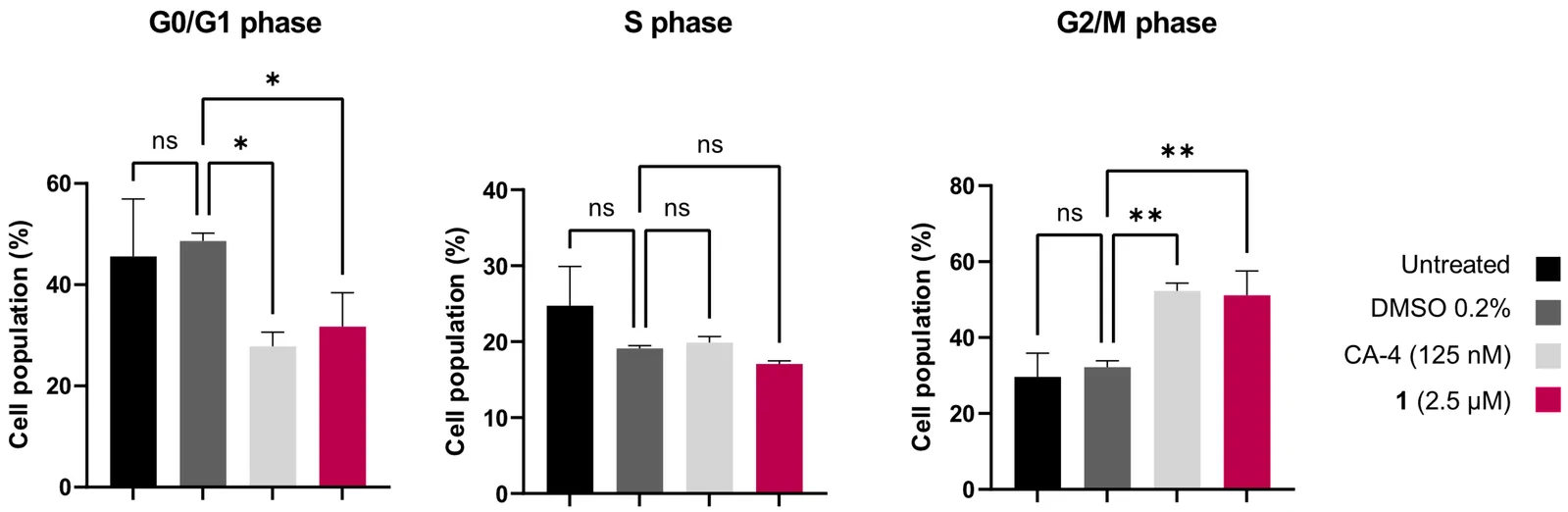
Cell cycle progression of MCF-7 cells treated with DMSO 0.2%, CA-4 at 125 nM and *N*-CIMZ **1** at 2.5 µM and untreated control cells at 24 h. *n* = 3 (independent biological experiments performed on different days), * *p* < 0.05, ** *p* < 0.01 and ns = non-significant. Error is expressed as the standard deviation. Experimental conditions containing *N*-CIMZ **1** or CA-4 contained a final concentration of DMSO (0.2%).

**Figure 5:**
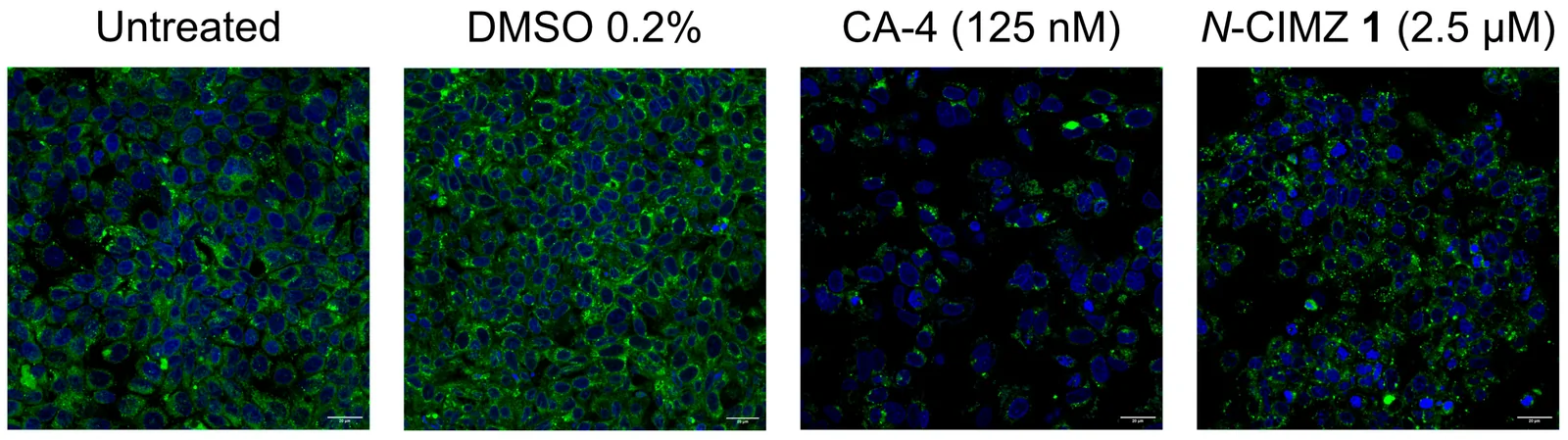
Effect of *N*-CIMZ **1** on the microtubule network of MCF-7 human breast cancer cells assessed by confocal immunofluorescence microscopy. Cells were left untreated, treated with vehicle (DMSO 0.2%), the reference antimitotic agent CA-4 (125 nM), or *N*-CIMZ **1** (2.5 µM) for 24 h. Microtubules are visualized in green (tubulin staining) and nuclei in blue (DAPI). Scale bars = 20 µm. Experimental conditions containing *N*-CIMZ **1** or CA-4 contained a final concentration of DMSO (0.2%).

**Figure 6:**
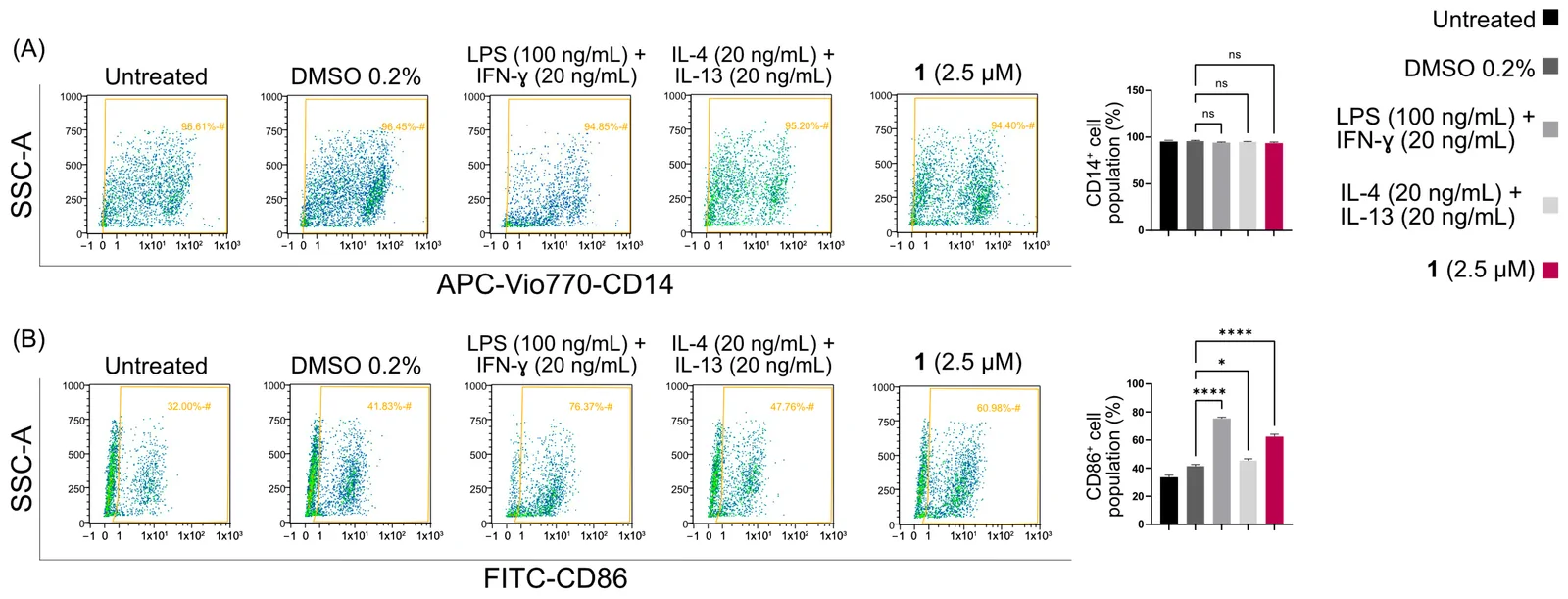
Representative scatter plots and corresponding summarizing histograms of THP-1-derived M0 macrophages after 24 h of treatment for (**A**) CD14 and (**B**) CD86 expression. Cells were treated with *N*-CIMZ **1** (2.5 µM). Untreated cells and DMSO 0.2% served as negative controls, while cells treated with LPS (100 ng/mL) + IFN-γ (20 ng/mL) or IL-13 and IL-4 (20 ng/mL each) were used as M1 and M2 positive controls respectively. *n* = 3 (independent biological experiments performed on different days), ns = non-significant, * *p* < 0.05 and **** *p* < 0.0001. Error is expressed as the standard deviation. All experimental conditions containing *N*-CIMZ **1** and/or the corresponding polarized groups contained a final concentration of DMSO (0.2%).

**Figure 7:**
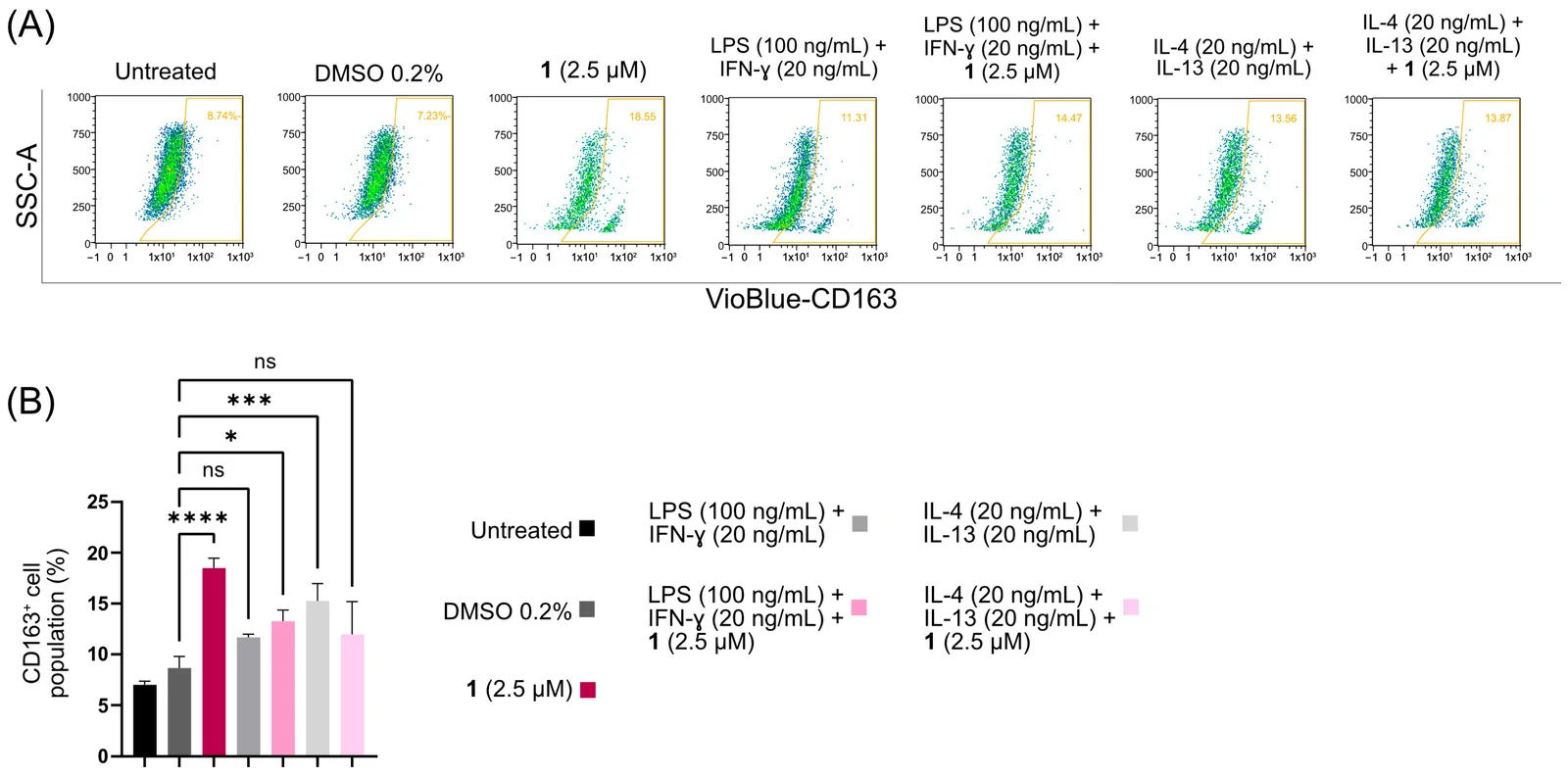
(**A**) Representative scatter plots and (**B**) corresponding summarizing histograms of CD163 expression on THP-1-derived M0 macrophages after 24 h of treatment with *N*-CIMZ **1** (2.5 µM) in monotherapy or combined with either M1-inducing cocktail (LPS 100 ng/mL + IFN-γ 20 ng/mL) or M2-inducing cocktail (IL-13 and IL-4 (20 ng/mL each)). Untreated cells and DMSO 0.2% served as negative controls, while cells treated with LPS (100 ng/mL) + IFN-γ (20 ng/mL) or IL-13 and IL-4 (20 ng/mL each) were used as M1 and M2 positive controls respectively. *n* = 3 (independent biological experiments performed on different days), ns = non-significant, * *p* < 0.05, *** *p* < 0.001 and **** *p* < 0.0001. Error is expressed as the standard deviation. All experimental conditions containing *N*-CIMZ **1** and/or the corresponding polarized groups contained a final concentration of DMSO (0.2%).

**Figure 8:**
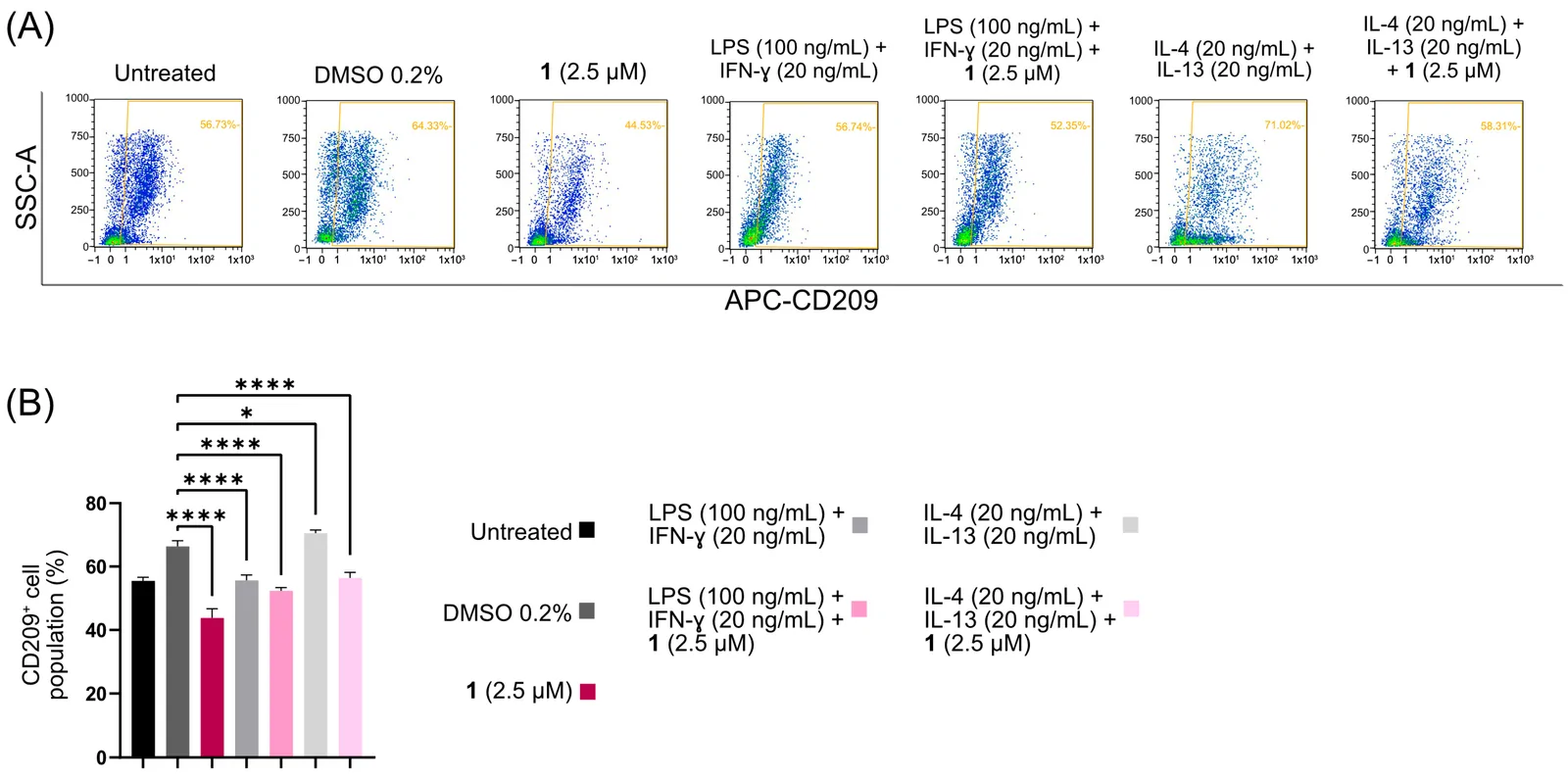
(**A**) Representative scatter plots and (**B**) corresponding summarizing histograms of CD209 expression on THP-1-derived M0 macrophages after 24 h of treatment with *N*-CIMZ **1** (2.5 µM) in monotherapy or combined with either M1-inducing cocktail (LPS 100 ng/mL + IFN-γ 20 ng/mL) or M2-inducing cocktail (IL-13 and IL-4 (20 ng/mL each)). Untreated cells and DMSO 0.2% served as negative controls, while cells treated with LPS (100 ng/mL) + IFN-γ (20 ng/mL) or IL-13 and IL-4 (20 ng/mL each) were used as M1 and M2 positive controls respectively. *n* = 3 (independent biological experiments performed on different days), * *p* < 0.05 and **** *p* < 0.0001. Error is expressed as the standard deviation. All experimental conditions containing *N*-CIMZ **1** and/or the corresponding polarized groups contained a final concentration of DMSO (0.2%).

**Figure 9:**
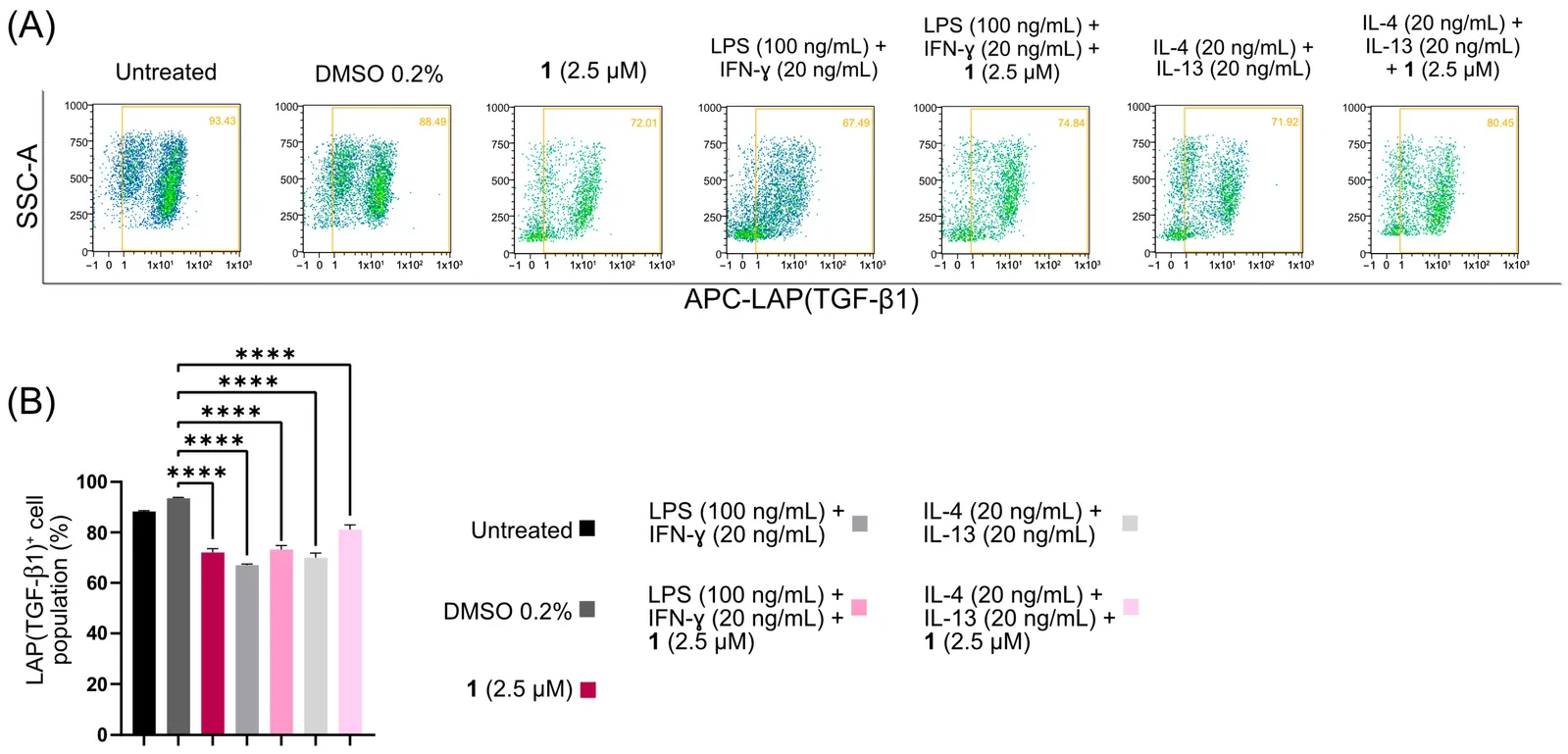
(**A**) Representative scatter plots and (**B**) corresponding summarizing histograms of LAP (TGF-β1) expression on THP-1-derived M0 macrophages after 24 h of treatment with *N*-CIMZ **1** (2.5 µM) in monotherapy or combined with either M1-inducing cocktail (LPS 100 ng/mL + IFN-γ 20 ng/mL) or M2-inducing cocktail (IL-13 and IL-4 (20 ng/mL each)). Untreated cells and DMSO 0.2% served as negative controls, while cells treated with LPS (100 ng/mL) + IFN-γ (20 ng/mL) or IL-13 and IL-4 (20 ng/mL each) were used as M1 and M2 positive controls respectively. *n* = 3 (independent biological experiments performed on different days), **** *p* < 0.0001. Error is expressed as the standard deviation. All experimental conditions containing *N*-CIMZ **1** and/or the corresponding polarized groups contained a final concentration of DMSO (0.2%).

**Figure 10:**
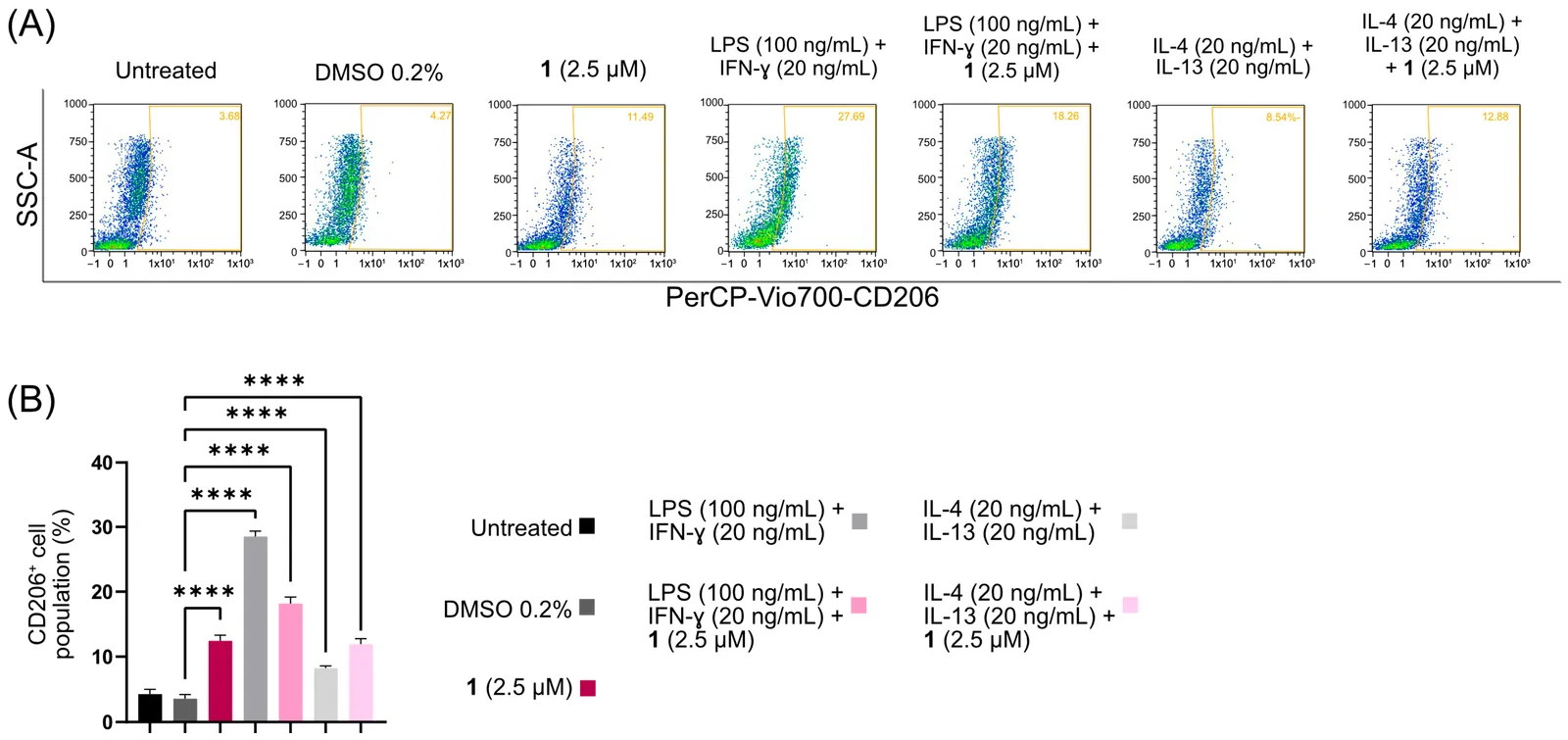
(**A**) Representative scatter plots and (**B**) corresponding summarizing histograms of CD206 expression on THP-1-derived M0 macrophages after 24 h of treatment with *N*-CIMZ **1** (2.5 µM) in monotherapy or combined with either M1-inducing cocktail (LPS 100 ng/mL + IFN-γ 20 ng/mL) or M2-inducing cocktail (IL-13 and IL-4 (20 ng/mL each)). Untreated cells and DMSO 0.2% served as negative controls, while cells treated with LPS (100 ng/mL) + IFN-γ (20 ng/mL) or IL-13 and IL-4 (20 ng/mL each) were used as M1 and M2 positive controls respectively. *n* = 3 (independent biological experiments performed on different days), **** *p* < 0.0001. Error is expressed as the standard deviation. All experimental conditions containing *N*-CIMZ **1** and/or the corresponding polarized groups contained a final concentration of DMSO (0.2%).

**Figure 11:**
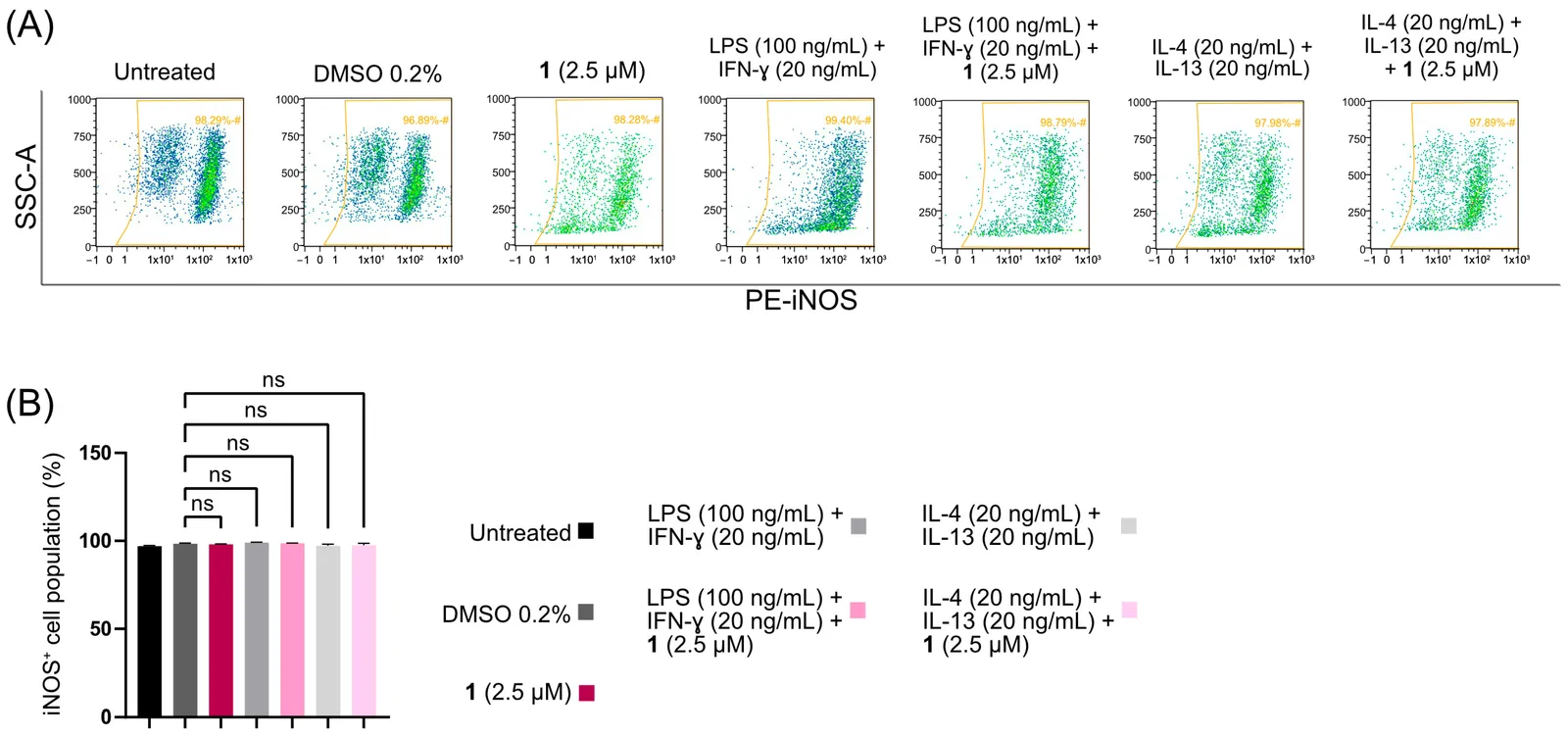
(**A**) Representative scatter plots and (**B**) corresponding summarizing histograms of iNOS expression on THP-1-derived M0 macrophages after 24 h of treatment with *N*-CIMZ **1** (2.5 µM) in monotherapy or combined with either M1-inducing cocktail (LPS 100 ng/mL + IFN-γ 20 ng/mL) or M2-inducing cocktail (IL-13 and IL-4 (20 ng/mL each)). Untreated cells and DMSO 0.2% served as negative controls, while cells treated with LPS (100 ng/mL) + IFN-γ (20 ng/mL) or IL-13 and IL-4 (20 ng/mL each) were used as M1 and M2 positive controls respectively. *n* = 3 (independent biological experiments performed on different days), ns = non-significant. Error is expressed as the standard deviation. All experimental conditions containing *N*-CIMZ **1** and/or the corresponding polarized groups contained a final concentration of DMSO (0.2%).

**Figure 12:**
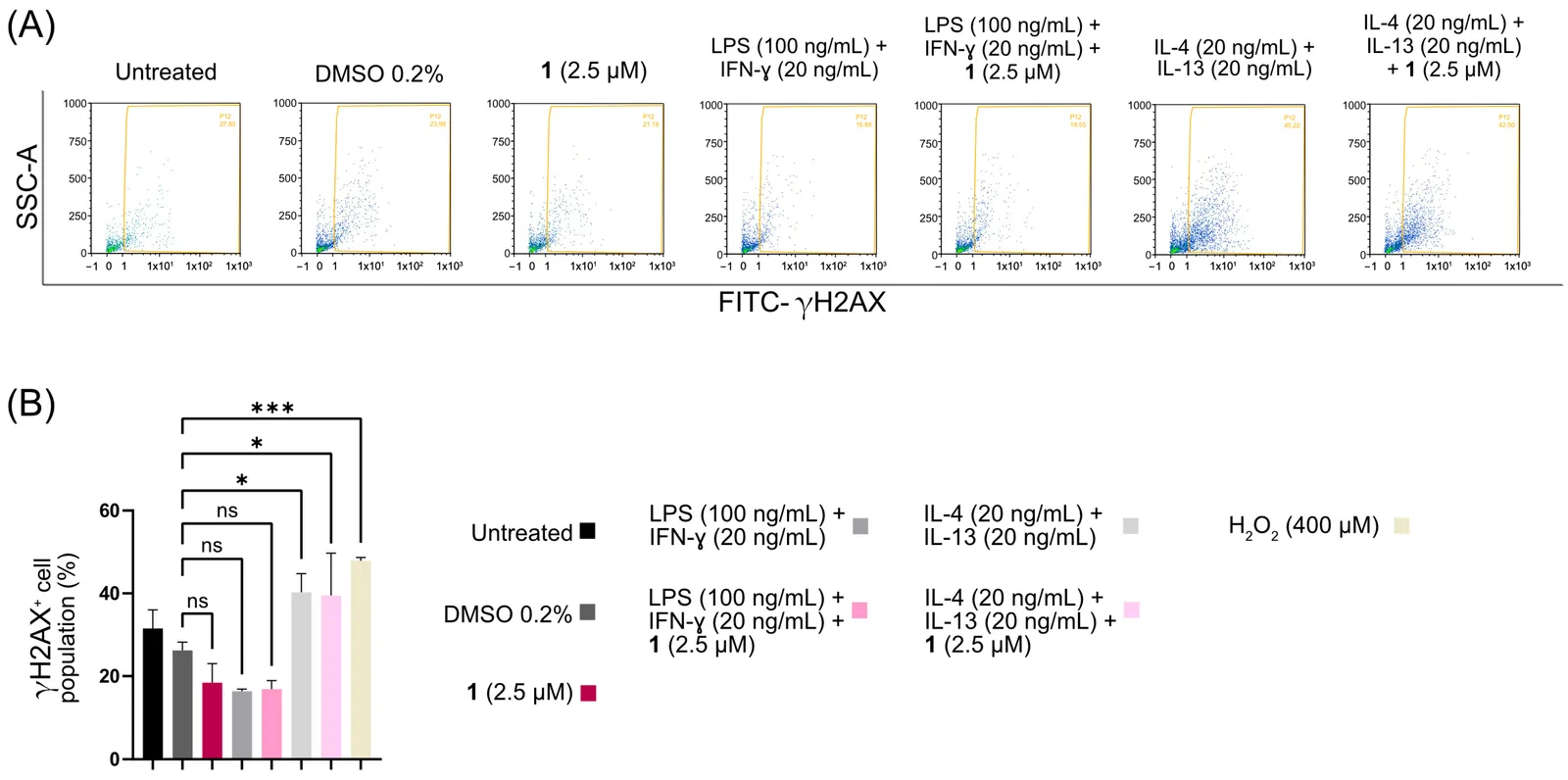
(**A**) Representative scatter plots and (**B**) corresponding summarizing histograms of γH2AX expression on THP-1-derived M0 macrophages after 24 h of treatment with *N*-CIMZ **1** (2.5 µM) in monotherapy or combined with either M1-inducing cocktail (LPS 100 ng/mL + IFN-γ 20 ng/mL) or M2-inducing cocktail (IL-13 and IL-4 (20 ng/mL each)). Untreated cells and DMSO 0.2% served as negative controls, while cells treated with LPS (100 ng/mL) + IFN-γ (20 ng/mL) or IL-13 and IL-4 (20 ng/mL each) were used as M1 and M2 positive controls respectively. *n* = 3 (independent biological experiments performed on different days), ns = non-significant, * *p* < 0.05 and *** *p* < 0.001. Error is expressed as the standard deviation. All experimental conditions containing *N*-CIMZ **1** and/or the corresponding polarized groups contained a final concentration of DMSO (0.2%).

**Figure 13:**
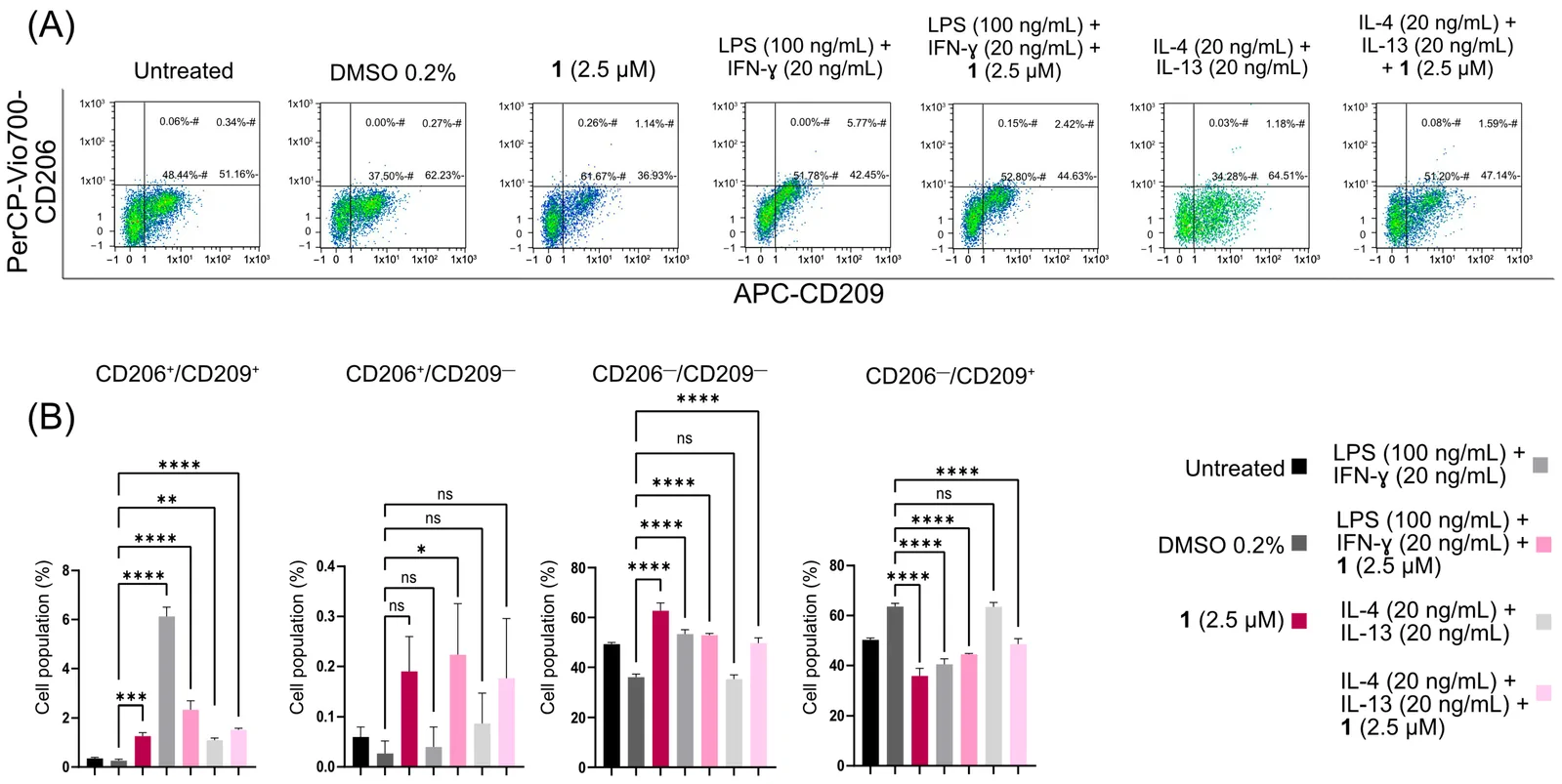
(**A**) Representative scatter plots and (**B**) corresponding summarizing histograms of CD206/CD209 simultaneous analysis on THP-1-derived M0 macrophages after 24 h of treatment with *N*-CIMZ **1** (2.5 µM) in monotherapy or combined with either M1-inducing cocktail (LPS 100 ng/mL + IFN-γ 20 ng/mL) or M2-inducing cocktail (IL-13 and IL-4 (20 ng/mL each)). Untreated cells and DMSO 0.2% served as negative controls, while cells treated with LPS (100 ng/mL) + IFN-γ (20 ng/mL) or IL-13 and IL-4 (20 ng/mL each) were used as M1 and M2 positive controls respectively. *n* = 3 (independent biological experiments performed on different days), ns = non-significant, * *p* < 0.05, ** *p* < 0.01, *** *p* < 0.001 and **** *p* < 0.0001. Error is expressed as the standard deviation. All experimental conditions containing *N*-CIMZ **1** and/or the corresponding polarized groups contained a final concentration of DMSO (0.2%).

**Figure 14:**
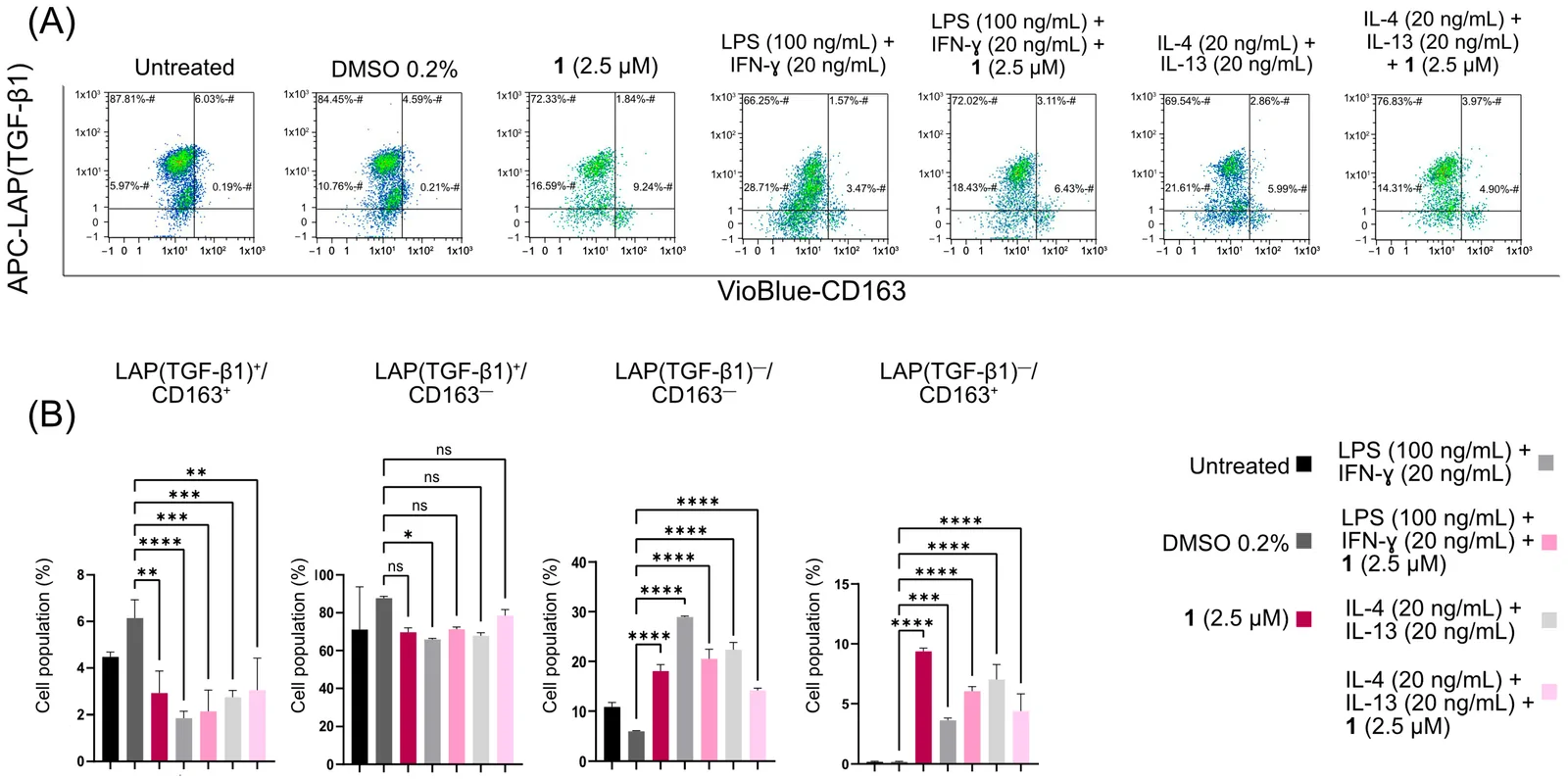
(**A**) Representative scatter plots and (**B**) corresponding summarizing histograms of LAP (TGF-β1)/CD163 simultaneous analysis on THP-1-derived M0 macrophages after 24 h of treatment with *N*-CIMZ **1** (2.5 µM) in monotherapy or combined with either M1-inducing cocktail (LPS 100 ng/mL + IFN-γ 20 ng/mL) or M2-inducing cocktail (IL-13 and IL-4 (20 ng/mL each)). Untreated cells and DMSO 0.2% served as negative controls, while cells treated with LPS (100 ng/mL) + IFN-γ (20 ng/mL) or IL-13 and IL-4 (20 ng/mL each) were used as M1 and M2 positive controls respectively. *n* = 3 (independent biological experiments performed on different days), ns = non-significant, * *p* < 0.05, ** *p* < 0.01, *** *p* < 0.001 and **** *p* < 0.0001. Error is expressed as the standard deviation. All experimental conditions containing *N*-CIMZ **1** and/or the corresponding polarized groups contained a final concentration of DMSO (0.2%).

**Table 1:** Antiproliferative activity (IC_50_) of *N*-CIMZs **1**–**8**, and CA-4 MCF-7, BT-20, MDA-MB-231, and MDA-MB-453 human cancer cells following 48 h exposure.

Comp.	Ring A	Ring B		IC_50_ (µM) *^a^*		
			MCF-7	BT-20	MDA-MB-231	MDA-MB-453
**1**	1,3	3,4,5-OMe	0.5 ± 0.1	2.5 ±0.2	3.0 ± 1.0	0.4 ± 0.1
**2**	1,3	2,6-Cl	1.8 ± 0.2	2.0 ± 1.0	4.0 ± 1.0	0.6 ± 0.3
**3**	1,3	2,5-Me	1.7 ± 0.3	0.4 ± 0.30	>2.0	0.3 ± 0.2
**4**	1,3	2,6-F	1.7 ± 0.1	3.0± 1.0	>5.0	4.0 ± 2.0
**5**	1,4	3,4,5-OMe	0.8 ± 0.2	3.0 ± 1.0	2.3 ± 0.3	1.4 ± 0.7
**6**	1,4	2,6-Cl	1.7 ± 0.4	4.0 ± 2.0	3.0 ± 2.0	0.5 ± 0.2
**7**	1,4	2,5-Me	0.6 ± 0.1	>2.0	>2.0	2.0 ± 0.5
**8**	1,4	2,6-F	>2.0	>2.0	>2.0	1.2 ± 0.2
CA-4	N.A.	N.A.	0.007 ± 0.002	0.0129 ± 0.0002	0.002 ± 0.001	0.0016 ± 0.0005

*^a^* IC_50_ is the concentration of the compound inhibiting the growth of cell lines by 50% after 48 h of treatment. The maximum evaluated concentration was 5.0 µM for *N*-CIMZs **1**, **2**, **4**, **5** and **6** and 2.0 µM for *N*-CIMZs **3**, **7** and **8**. Values reported as >2.0 µM or >5.0 µM indicate that the IC_50_ was not reached within the highest concentration tested for the corresponding compound. N.A.: not applicable.

## Data Availability

The original contributions presented in this study are included in the article/Supplementary Materials. Further inquiries can be directed to the corresponding authors.

## Supplementary Materials

The supporting information can be downloaded at: https://www.mdpi.com/article/10.3390/ph19101503/s1, Table S1. Detailed statistical analysis of MCF-7 scratch-wound closure dynamics. Figure S1. Membrane integrity assessment and dead cell exclusion strategy for flow cytometry.

## Abbreviations

The following abbreviations are used in this manuscript:

BC	Breast cancer
CA-4	Combretastatin A-4
DMSO	Dimethyl sulfoxide
HRMS	High-resolution mass spectrometry
IFN-γ	Interferon gamma
IL-4	Interleukin 4
IL-13	Interleukin 13
LPS	Lipopolysaccharide
MDR	Multidrug resistance
N-CIMZs	N-chalconyl imidazolidones
TAMs	Tumor-associated macrophage
TME	Tumor microenvironment
TNBC	Triple-negative breast cancer
PBS	Phosphate-buffered saline
PMA	Phorbol 12-myristate 13-acetate
ROS	Reactive oxygen species
