# Supplementary material for: Synthesis of Antiproliferative N-Chalconyl Imidazolidones and Mechanistic Evaluation of Prototype N-CIMZ 1 as a Macrophage-Modulating Antimitotic Agent
Source: Pharmaceuticals (Basel). 2026 Sep 22;19(10):1503. doi: 10.3390/ph19101503 (PMC13708499; doi:10.3390/ph19101503)
Supplement: Supplementary file 1 [file pharmaceuticals-19-01503-s001.zip › pharmaceuticals-4528576-supplementary.pdf]

Supporting information

Synthesis of antiproliferative *N*-chalconyl imidazolidones and mechanistic evaluation of prototype *N*-CIMZ 1 as a macrophage-modulating antimitotic agent

Atziri Corin Chavez Alvarez[1,*], Lisa Treboux[1], Céline Audrey Béchon-Diot[1], Rayan Chkair[1], Sophie Besse[1], Emmanuel Moreau[1,*]

[1]UMR-1240 Inserm-Université Clermont Auvergne, 58 rue Montalembert, 63005 Clermont-Ferrand, France. (A.C.C.A.) atziri_corin.chavez_alvarez@uca.fr, (L.T.) lisa.treboux@etu.uca.fr, (C. A. B.-D.) celine_audrey.bechon_diot@uca.fr, (R. C.) rayan.chkair@uca.fr, (S.B.) sophie.besse@inserm.fr, (E.M.) emmanuel.moreau@uca.fr

* Correspondence: (A.C.C.A.) atziri_corin.chavez_alvarez@uca.fr, (E.M.) emmanuel.moreau@uca.fr

Table S1. Detailed statistical analysis of MCF-7 scratch-wound closure dynamics **Page 2-3**

Figure S1. Membrane integrity assessment and dead cell exclusion strategy for flow cytometry **Page 4**

^1H NMR, ^{13}C NMR and HRMS spectra, and UHPLC chromatograms

Compound 1 **Page 5-6**
Compound 2 **Page 7-8**
Compound 3 **Page 9-10**
Compound 4 **Page 11-12**
Compound 5 **Page 13-14**
Compound 6 **Page 15-16**
Compound 7 **Page 17-18**
Compound 8 **Page 19-20**

Table S1. Detailed statistical analysis of MCF-7 scratch-wound closure dynamics. Complete computational output from the Two-Way Repeated Measures (RM) ANOVA and Tukey's multiple comparisons test utilized to evaluate the wound-healing assay. The upper section provides the comprehensive ANOVA summary of the time-dependent pharmacological effect of the treatments on monolayer expansion. The lower section details the post-hoc multiple comparisons across all evaluated time points (12, 24, and 36 h).

Two-way RM ANOVA	Matching: Stacked							
Assume sphericity?	Yes							
Alpha	0.05							
Source of Variation	% of total variation	P value	P value summary	Significant?				
Time x Treatment	6.919	0.0221	*	Yes				
Time	35.75	<0.0001	****	Yes				
Treatment	31.3	0.0509	ns	No				
Subject	20.69	0.0003	***	Yes				
ANOVA table	SS	DF	MS	F (DFn, DFd)	P value			
Time x Treatment	182	6	30.33	F (6, 16) = 3.451	P=0.0221			
Time	940.3	2	470.2	F (2, 16) = 53.49	P<0.0001			
Treatment	823.1	3	274.4	F (3, 8) = 4.034	P=0.0509			
Subject	544.1	8	68.01	F (8, 16) = 7.738	P=0.0003			
Residual	140.6	16	8.789					
Data summary								
Number of columns (Treatment)	4							
Number of rows (Time)	3							
Number of subjects (Subject)	12							
Number of missing values	0							
Tukey's multiple comparisons test	Mean diff.	95.00% CI of diff.	Below t hreshold?	Summary	Adjusted P Value			
12								
Untreated vs. DMSO 0.2%	-0.26	-12.29 to 11.77	No	ns	>0.9999			
DMSO 0.2% vs. *N*-CIMZ **1**	3.61	-8.420 to 15.64	No	ns	0.8408			
DMSO 0.2% vs. CA-4	6.157	-5.874 to 18.19	No	ns	0.5045			
N-CIMZ **1** vs. CA-4	2.547	-9.484 to 14.58	No	ns	0.9359			

24								
Untreated vs. DMSO 0.2%	-11.25	-23.28 to 0.7838	No	ns	0.0728			
DMSO 0.2% vs. *N*-CIMZ **1**	15.87	3.843 to 27.90	Yes	**	0.0067			
DMSO 0.2% vs. CA-4	14.42	2.390 to 26.45	Yes	*	0.0147			
N-CIMZ **1** vs. CA-4	-1.453	-13.48 to 10.58	No	ns	0.9869			
36								
Untreated vs. DMSO 0.2%	-8.393	-20.42 to 3.637	No	ns	0.2446			
DMSO 0.2% vs. *N*-CIMZ **1**	16.42	4.393 to 28.45	Yes	**	0.0049			
DMSO 0.2% vs. CA-4	13.34	1.310 to 25.37	Yes	*	0.0259			
N-CIMZ **1** vs. CA-4	-3.083	-15.11 to 8.947	No	ns	0.8933			
Test details	Mean 1	Mean 2	Mean diff.	SE of diff.	N1	N2	q	DF
12								
Untreated vs. DMSO 0.2%	33.77	34.03	-0.26	4.361	3	3	0.08431	24
DMSO 0.2% vs. *N*-CIMZ **1**	34.03	30.42	3.61	4.361	3	3	1.171	24
DMSO 0.2% vs. CA-4	34.03	27.88	6.157	4.361	3	3	1.996	24
N-CIMZ **1** vs. CA-4	30.42	27.88	2.547	4.361	3	3	0.8258	24
24								
Untreated vs. DMSO 0.2%	37.22	48.46	-11.25	4.361	3	3	3.647	24
DMSO 0.2% vs. *N*-CIMZ **1**	48.46	32.59	15.87	4.361	3	3	5.147	24
DMSO 0.2% vs. CA-4	48.46	34.04	14.42	4.361	3	3	4.676	24
N-CIMZ **1** vs. CA-4	32.59	34.04	-1.453	4.361	3	3	0.4713	24
36								
Untreated vs. DMSO 0.2%	45.19	53.58	-8.393	4.361	3	3	2.722	24
DMSO 0.2% vs. *N*-CIMZ **1**	53.58	37.16	16.42	4.361	3	3	5.326	24
DMSO 0.2% vs. CA-4	53.58	40.24	13.34	4.361	3	3	4.326	24
N-CIMZ **1** vs. CA-4	37.16	40.24	-3.083	4.361	3	3	0.9999	24

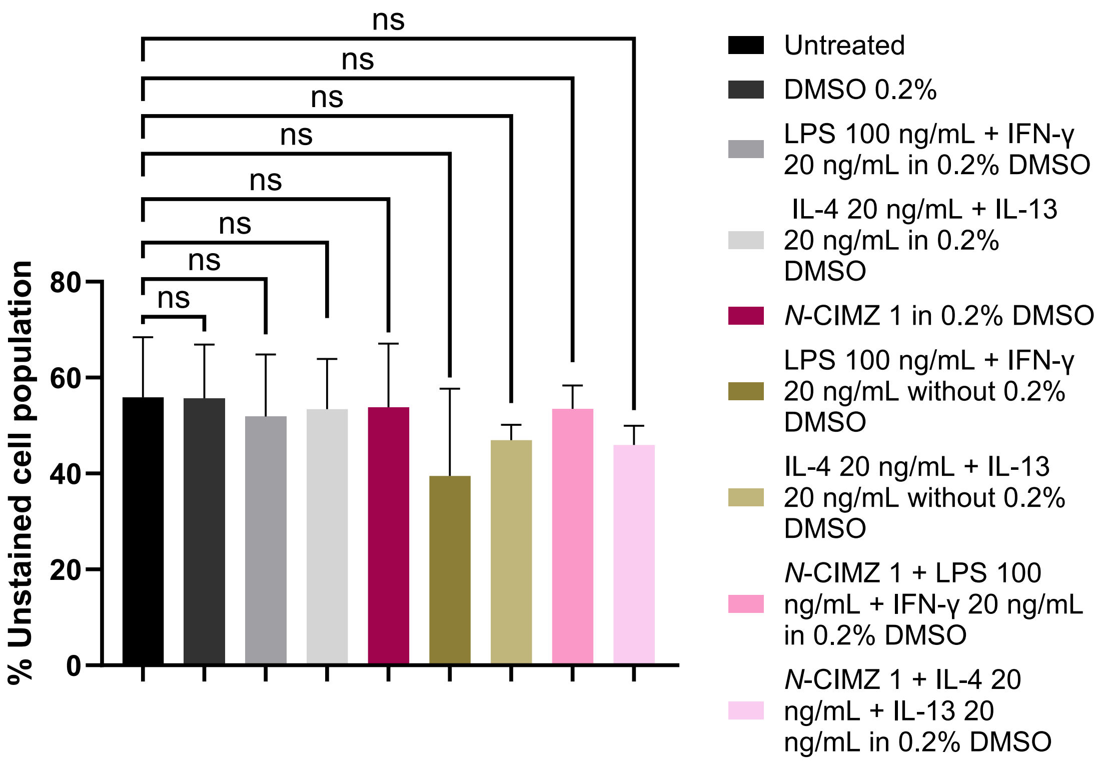

Figure S1. Membrane integrity assessment and dead cell exclusion strategy for flow cytometry. The percentage of the unstained cell population (representing structurally intact cells negative for the Viobility™ dye) was quantified to validate the dead cell exclusion gate prior to downstream flow cytometry analysis. Macrophages were exposed to vehicle (0.2% DMSO), polarization cocktails (LPS/IFN-γ or IL-4/IL-13), the prototype candidate N-CIMZ 1, or their respective combinations. Data represent the mean ± standard deviation (SD) of independent biological replicates. Statistical significance was determined using a One-Way ANOVA followed by Dunnett's multiple comparisons test against the untreated control group. ns = not significant ($p > 0.05$), confirming that none of the experimental treatments induced significant loss of plasma membrane integrity compared to the baseline control.

Compound 1

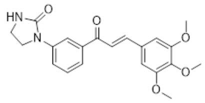
HN
O
N
O
O
O
O

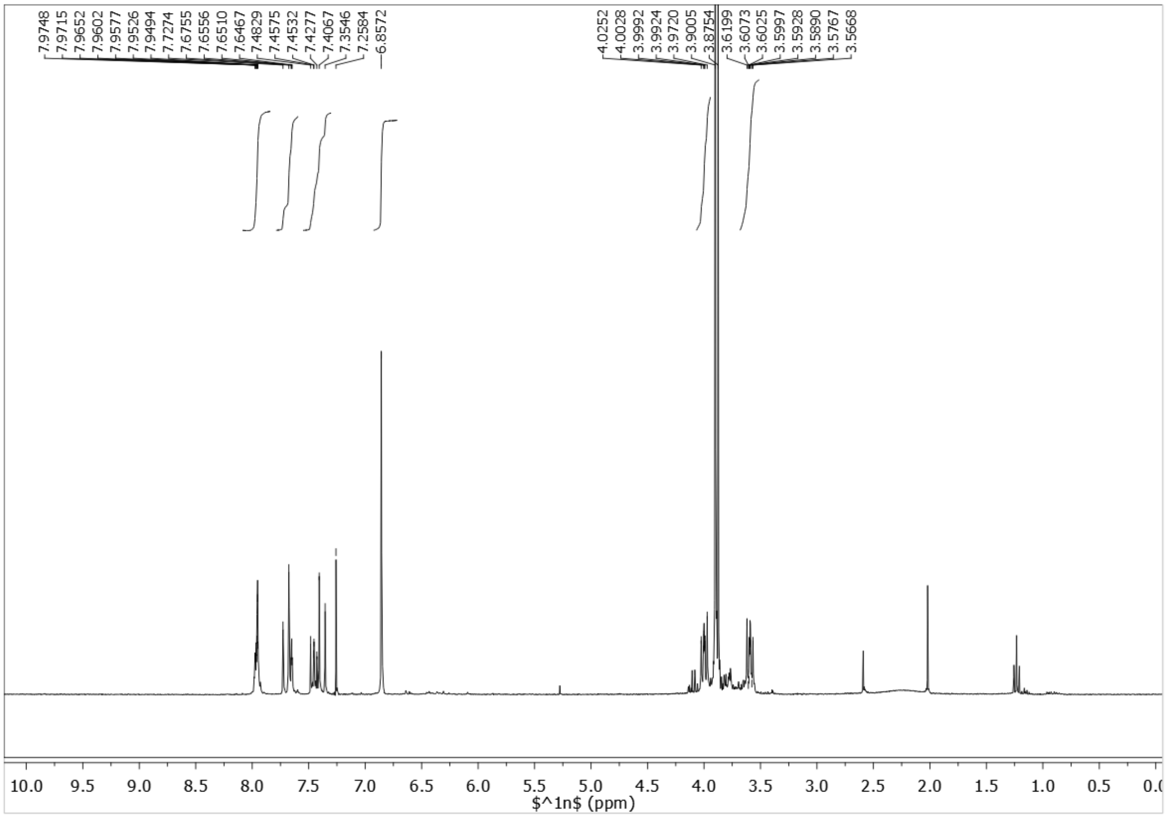
7.9748
7.9715
7.9652
7.9602
7.9577
7.9526
7.9494
7.7274
7.6755
7.6556
7.6510
7.6467
7.4829
7.4575
7.4532
7.4277
7.4067
7.3546
7.2584
6.8572
4.0252
4.0028
3.9992
3.9924
3.9720
3.9005
3.8754
3.6199
3.6073
3.6025
3.5997
3.5928
3.5890
3.5767
3.5668
10.0 9.5 9.0 8.5 8.0 7.5 7.0 6.5 6.0 5.5 5.0 4.5 4.0 3.5 3.0 2.5 2.0 1.5 1.0 0.5
^1n (ppm)

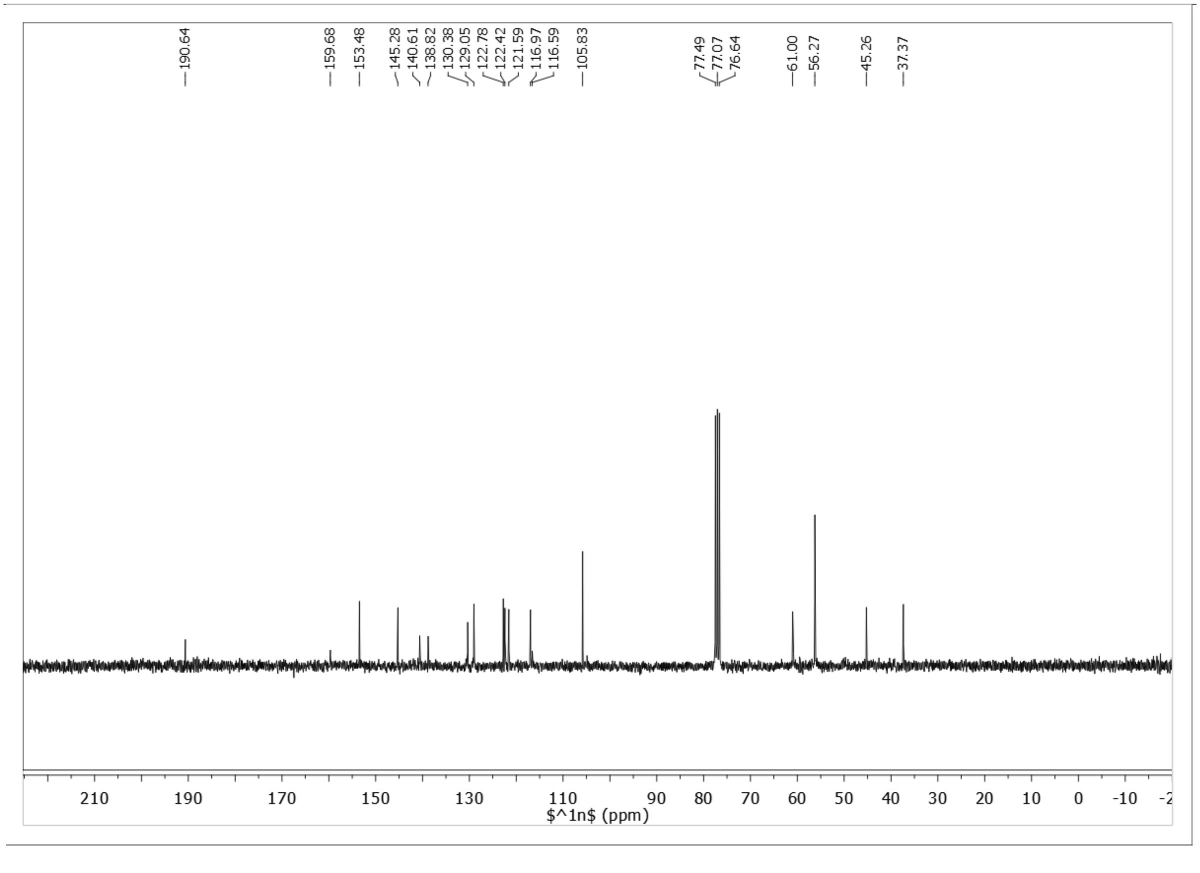
190.64
159.68
153.48
145.28
140.61
138.82
130.38
129.05
122.78
122.42
121.59
116.97
116.59
105.83
77.49
77.07
76.64
61.00
56.27
45.26
37.37
210 190 170 150 130 110 90 80 70 60 50 40 30 20 10 0 -10
^1n (ppm)

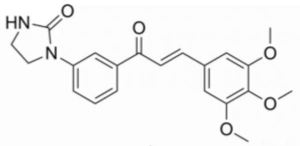

1

MS spectrum peak list

m/z	Calc m/z	Diff(ppm)	z	Abund	Formula	Ion
383.1596	383.1601	-1.48		657658	C21 H23 N2 O5	(M+H)+
383.4021				48593		
383.5158				3886		
383.6466				4998		
383.8884				1764		
384.1624	384.1633	-2.44		151617	C21 H23 N2 O5	(M+H)+
384.4043				7050		
385.1657	385.166	-0.65		20586	C21 H23 N2 O5	(M+H)+
386.1691	386.1685	1.54		2533	C21 H23 N2 O5	(M+H)+
400.1673	400.1867	-48.54		349	C21 H26 N3 O5	(M+NH4)+

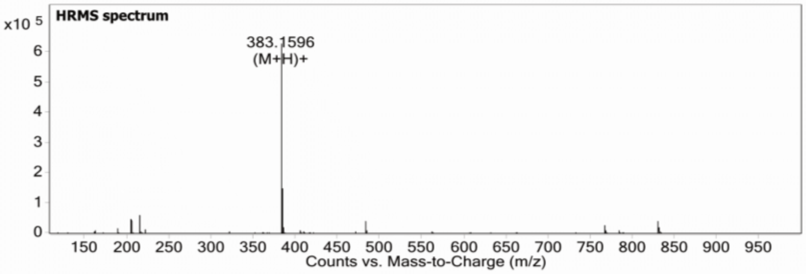

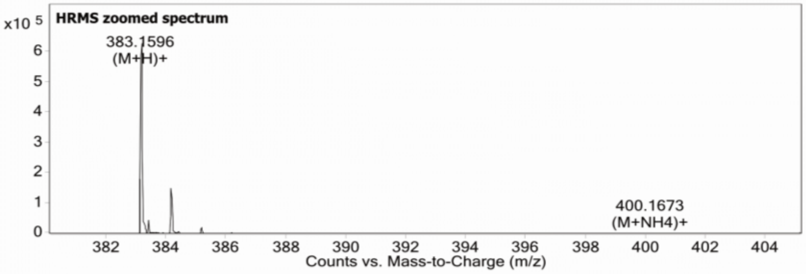

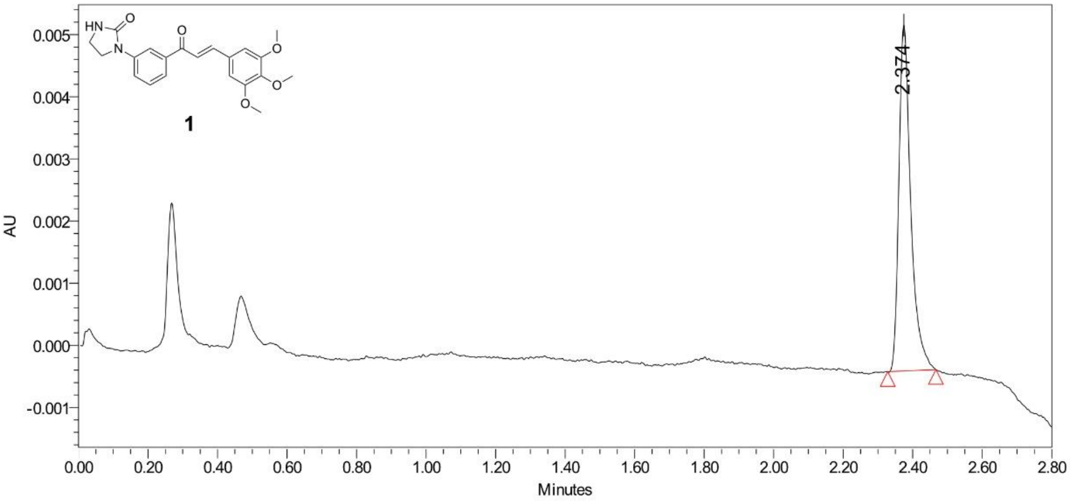

Peak Results

	RT	Area	Height	% Area	% Height
1	2.374	13223	5570	100.00	100.00

Compound 2

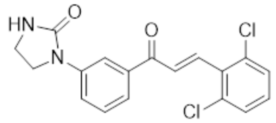

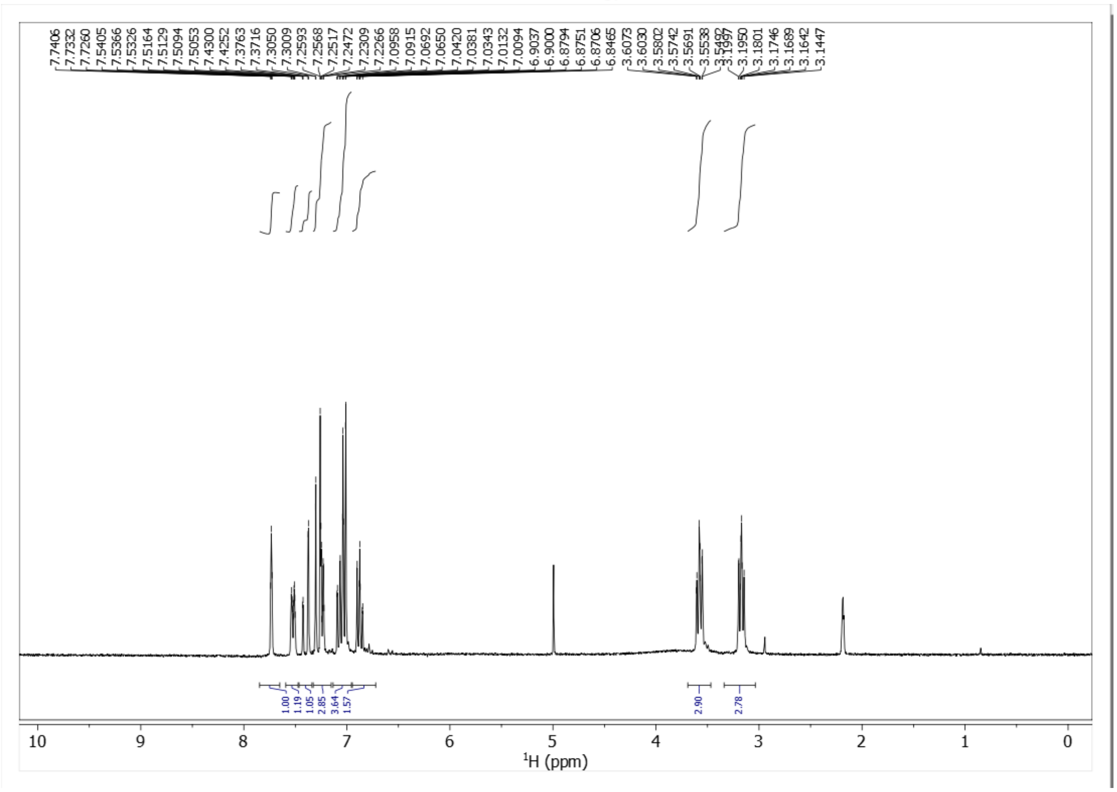

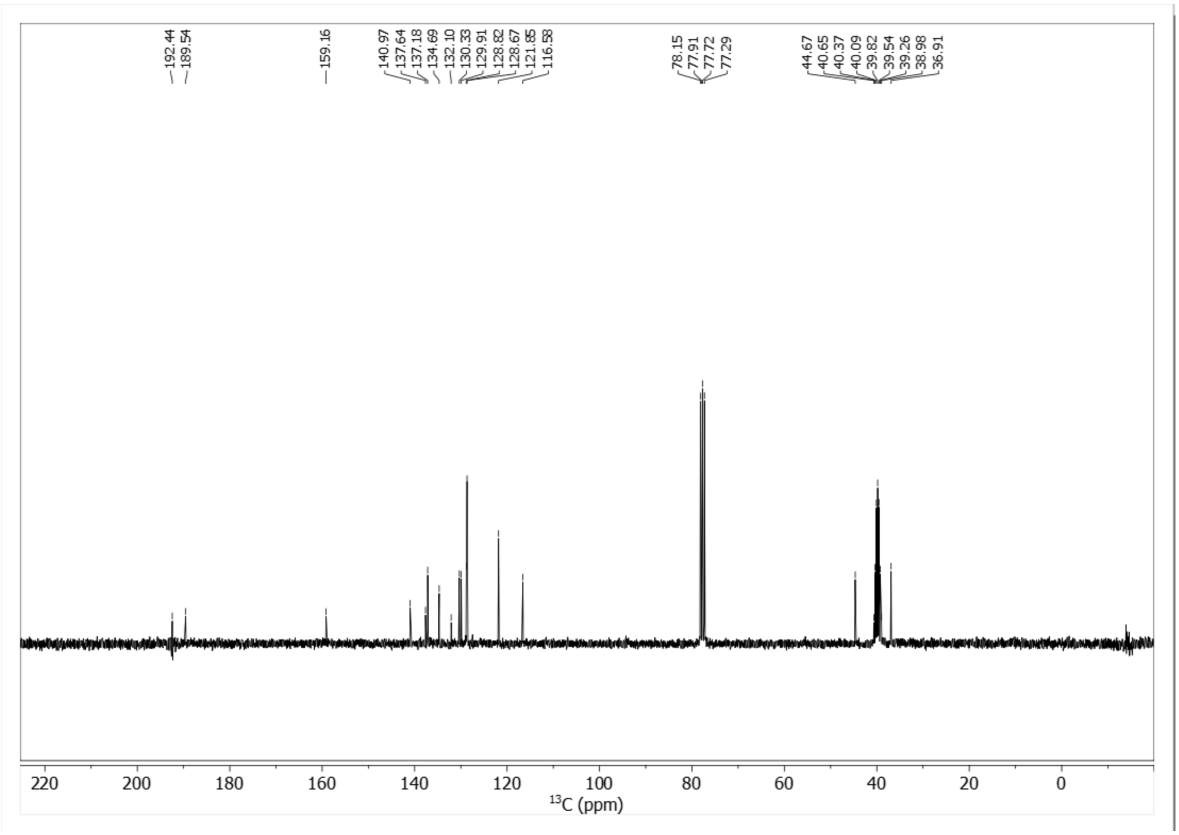

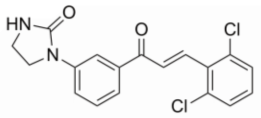

2

MS spectrum peak list

m/z	*Calc m/z*	Diff(ppm)	z	Abund	Formula	Ion
361.0497	361.0505	-2.13		675438	C18 H15 Cl2 N2 O2	(M+H)+
361.2844				51886		
362.0524	362.0537	-3.47		121004	C18 H15 Cl2 N2 O2	(M+H)+
363.047	363.0479	-2.53		456881	C18 H15 Cl2 N2 O2	(M+H)+
363.2833				32077		
364.0497	364.0508	-3.08		76681	C18 H15 Cl2 N2 O2	(M+H)+
365.0446	365.0458	-3.37		63381	C18 H15 Cl2 N2 O2	(M+H)+
366.047	366.0483	-3.56		10460	C18 H15 Cl2 N2 O2	(M+H)+
378.0758	378.0771	-3.29		22698	C18 H18 Cl2 N3 O2	(M+NH4)+
380.0734	380.0744	-2.68		13934	C18 H18 Cl2 N3 O2	(M+NH4)+

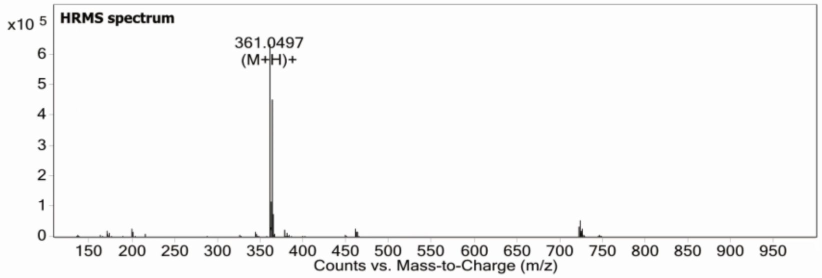

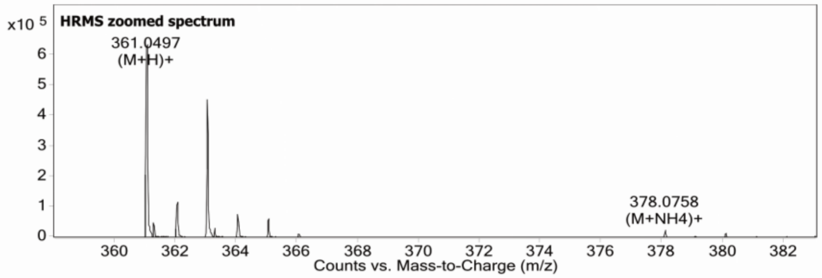

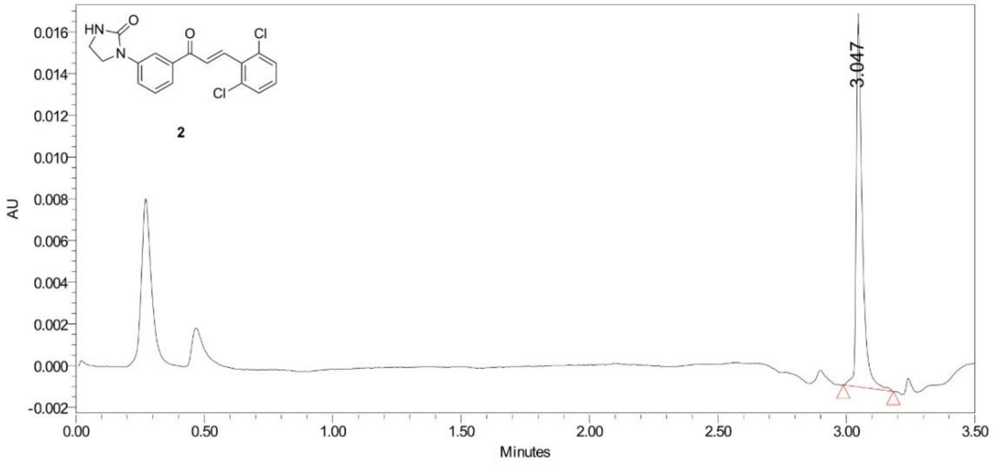

Peak Results

	RT	Area	Height	% Area	% Height
1	3.047	28819	17419	100.00	100.00

Compound 3

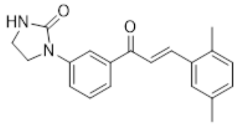
HN
O
N
O

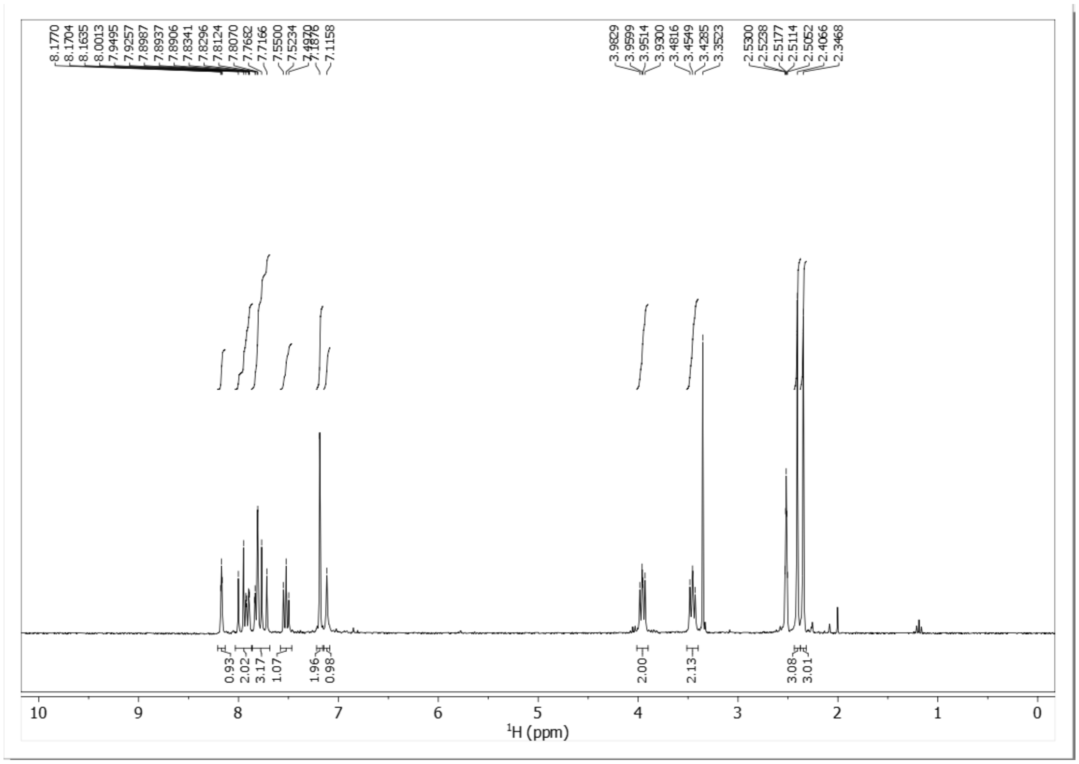
8.1770
8.1704
8.1635
8.0013
7.9495
7.9257
7.8987
7.8937
7.8906
7.8341
7.8296
7.8124
7.8070
7.7682
7.7166
7.5500
7.5234
7.1158
3.9829
3.9599
3.9514
3.9300
3.4816
3.4549
3.4285
3.3523
2.5300
2.5238
2.5177
2.5114
2.5052
2.4066
2.3468
0.93
2.02
3.17
1.07
1.96
0.98
2.00
2.13
3.08
3.01
10
9
8
7
6
5
4
3
2
1
0
¹H (ppm)

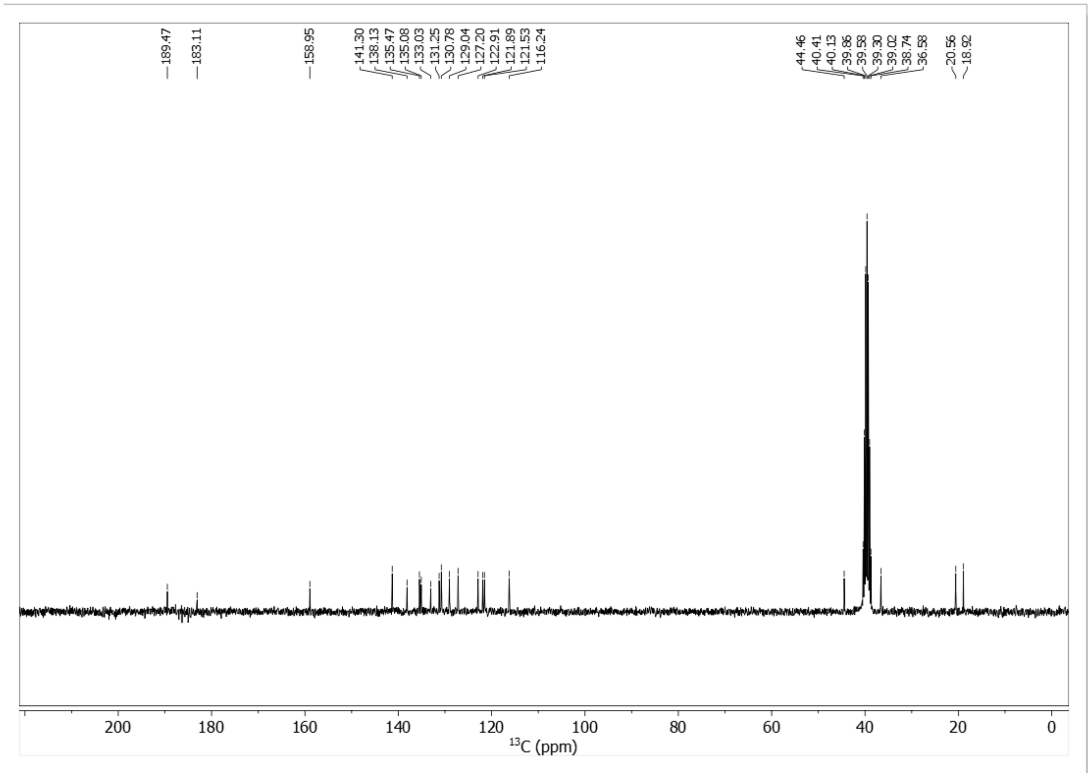
189.47
183.11
158.95
141.30
138.13
135.47
135.08
133.03
131.25
130.78
129.04
127.20
122.91
121.89
121.53
116.24
44.46
40.41
40.13
39.86
39.58
39.30
39.02
38.74
36.58
20.56
18.92
200
180
160
140
120
100
80
60
40
20
0
¹³C (ppm)

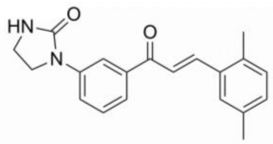

3

MS spectrum peak list

m/z	*Calc m/z*	Diff(ppm)	Abund	Formula	Ion
321.1592	321.1598	-1.77	930315	C20 H21 N2 O2	(M+H)+
321.3799			90881		
321.4973			6327		
321.6023			10132		
321.7088			3514		
321.8264			3131		
322.1611	322.1629	-5.76	212818	C20 H21 N2 O2	(M+H)+
322.3824			13565		
322.4837			2380		
323.1646	323.1658	-3.83	23223	C20 H21 N2 O2	

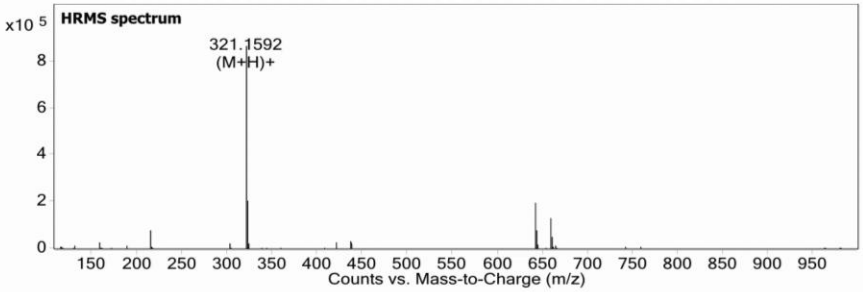

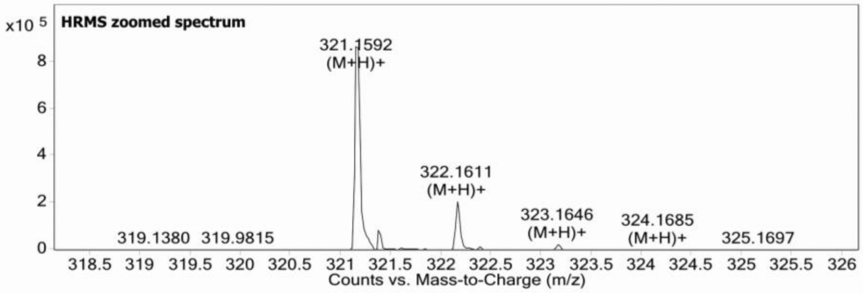

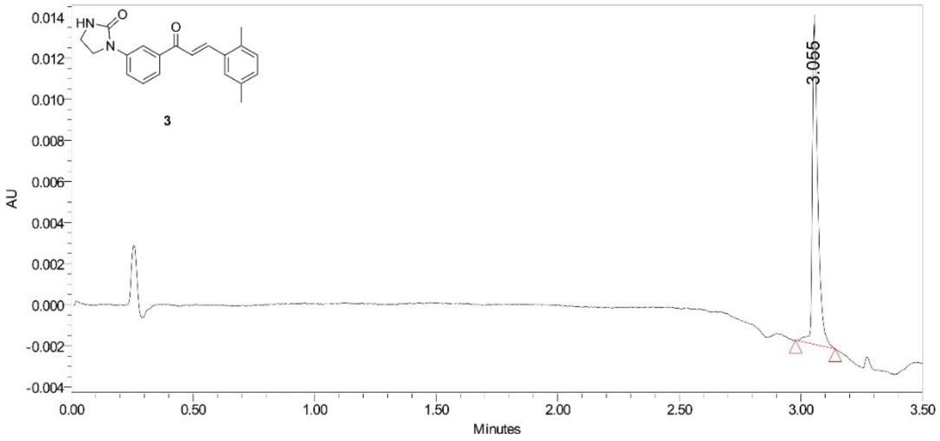

Peak Results

	RT	Area	Height	% Area	% Height
1	3.055	24821	15647	100.00	100.00

Compound 4

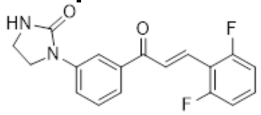

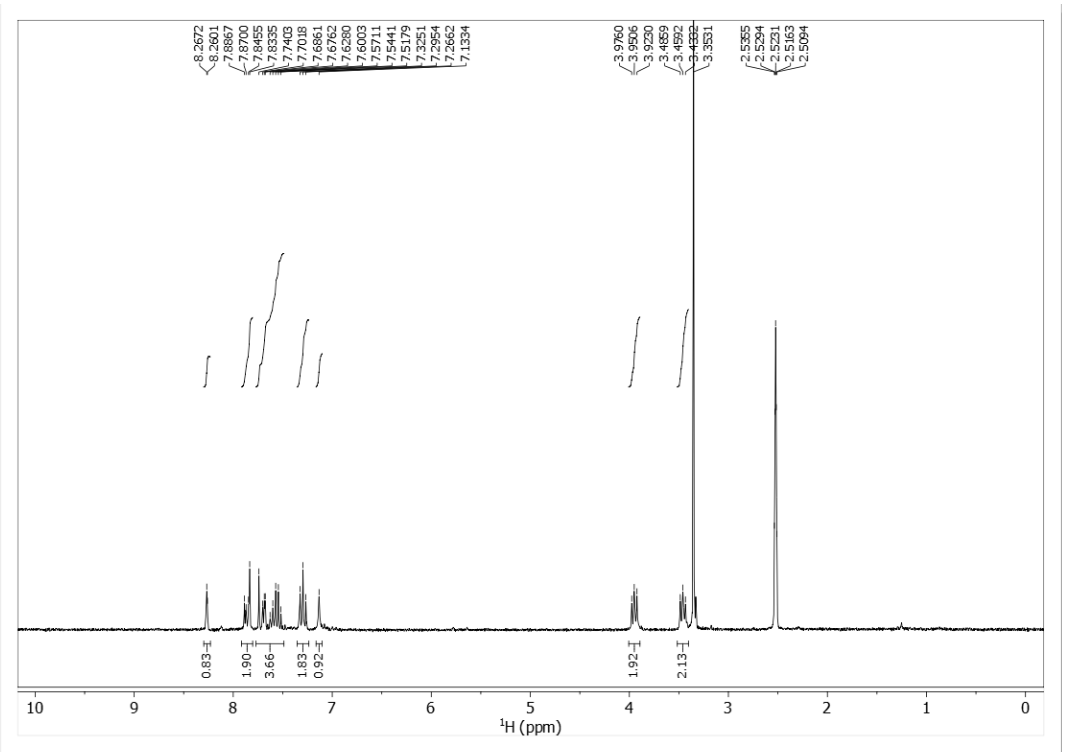

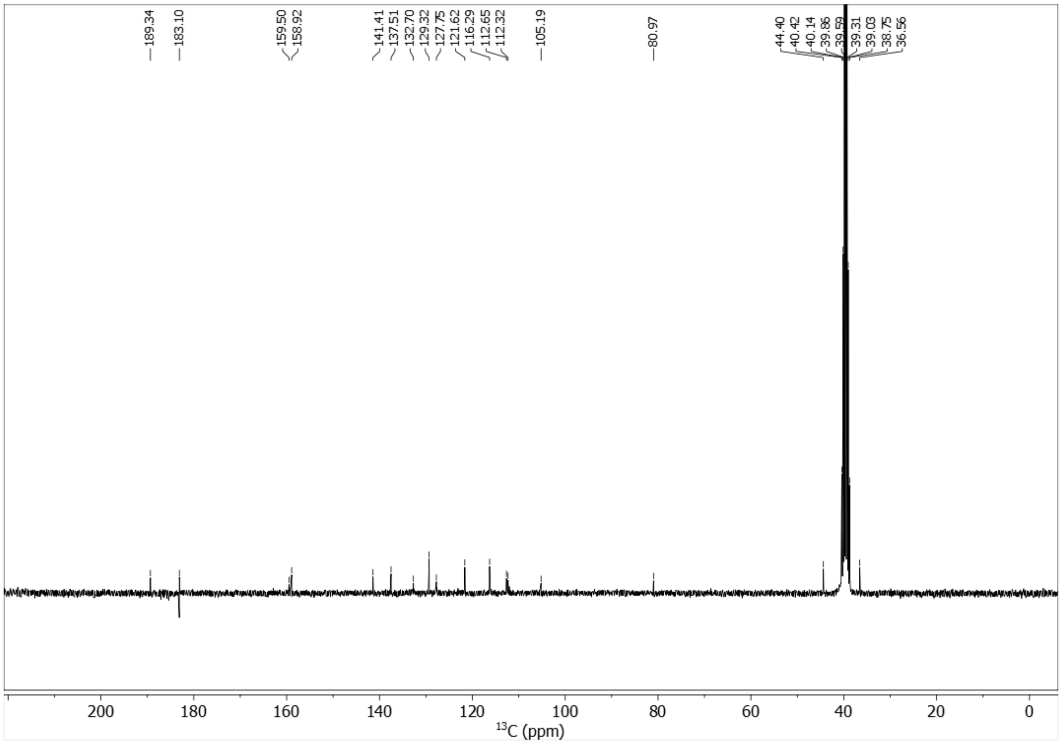

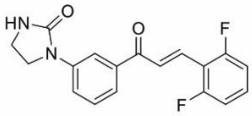

4

MS spectrum peak list

m/z	Calc m/z	Diff(ppm)	Abund	Formula	Ion
325.096			18460		
327.1037			16160		
329.1104	329.1096	2.31	1407933	C18 H15 F2 N2 O2	(M+H)+
329.3326			163726		
329.4589			13698		
329.5577			22022		
329.7847			6291		
330.1102	330.1128	-7.63	332669	C18 H15 F2 N2 O2	(M+H)+
330.338			23504		
331.113	331.1156	-7.68	32241	C18 H15 F2 N2 O2	

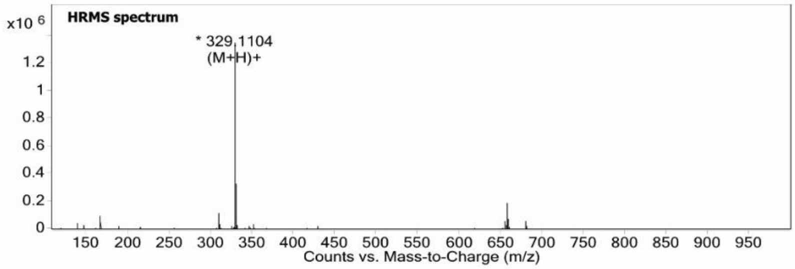

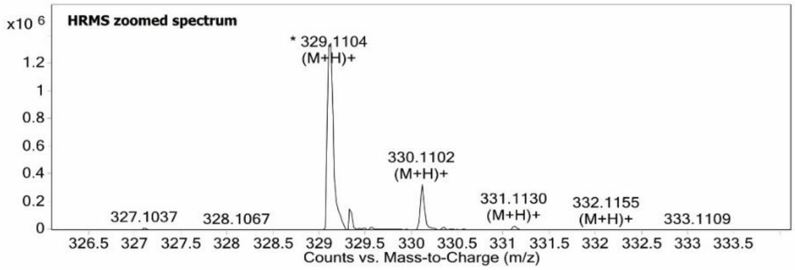

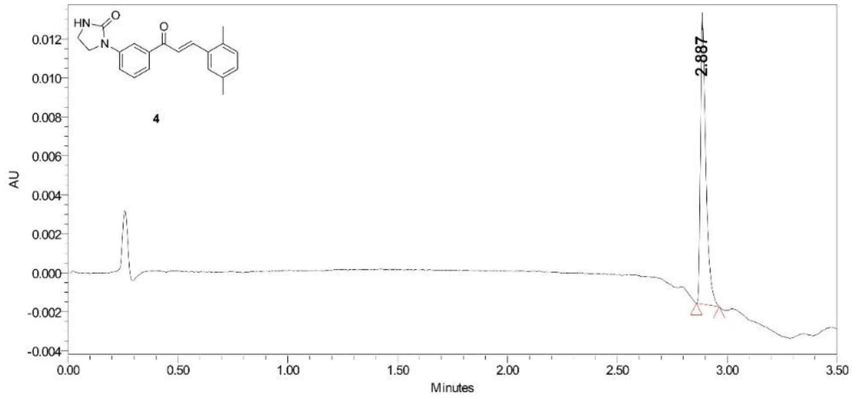

Peak Results

	RT	Area	Height	% Area	% Height
1	2.887	25291	14578	100.00	100.00

Compound 5

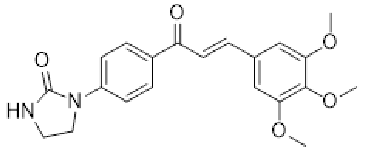

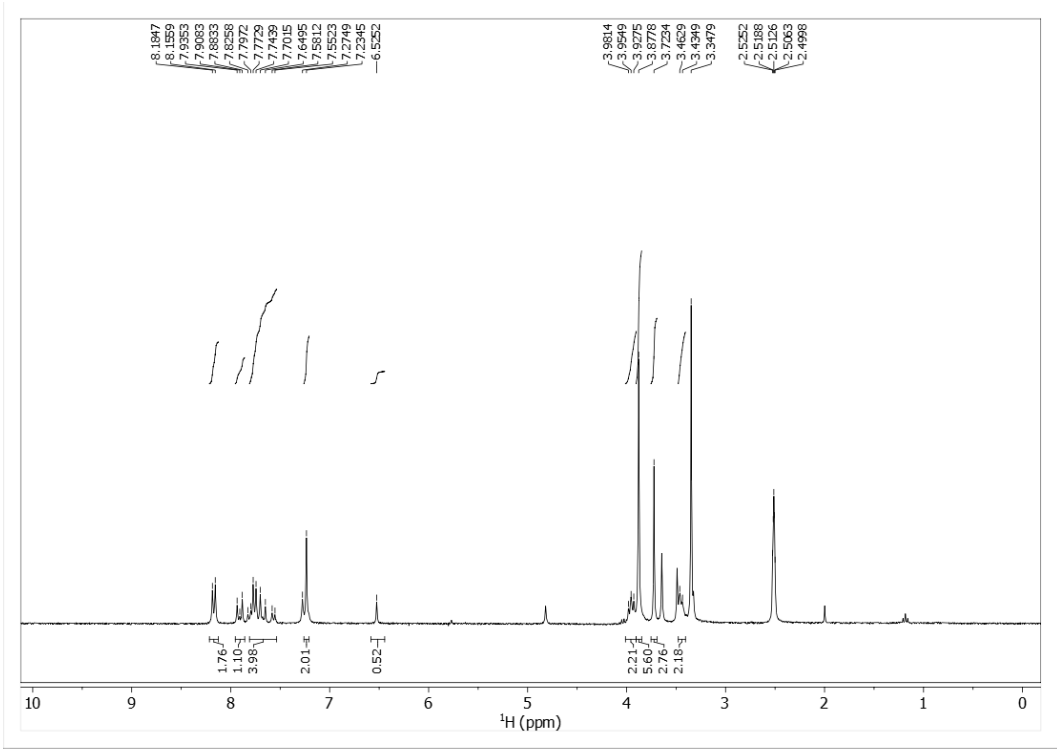

8.1847
8.1559
7.9353
7.9083
7.8833
7.8258
7.7972
7.7729
7.7439
7.7015
7.6495
7.5812
7.5523
7.2749
7.2345
6.5252
3.9814
3.9549
3.9275
3.8778
3.7234
3.4629
3.4349
3.3479
2.5252
2.5188
2.5126
2.5063
2.4998
1.76
1.10
3.98
2.01
0.52
2.21
5.60
2.76
2.18
10
9
8
7
6
5
4
3
2
1
0
1H (ppm)

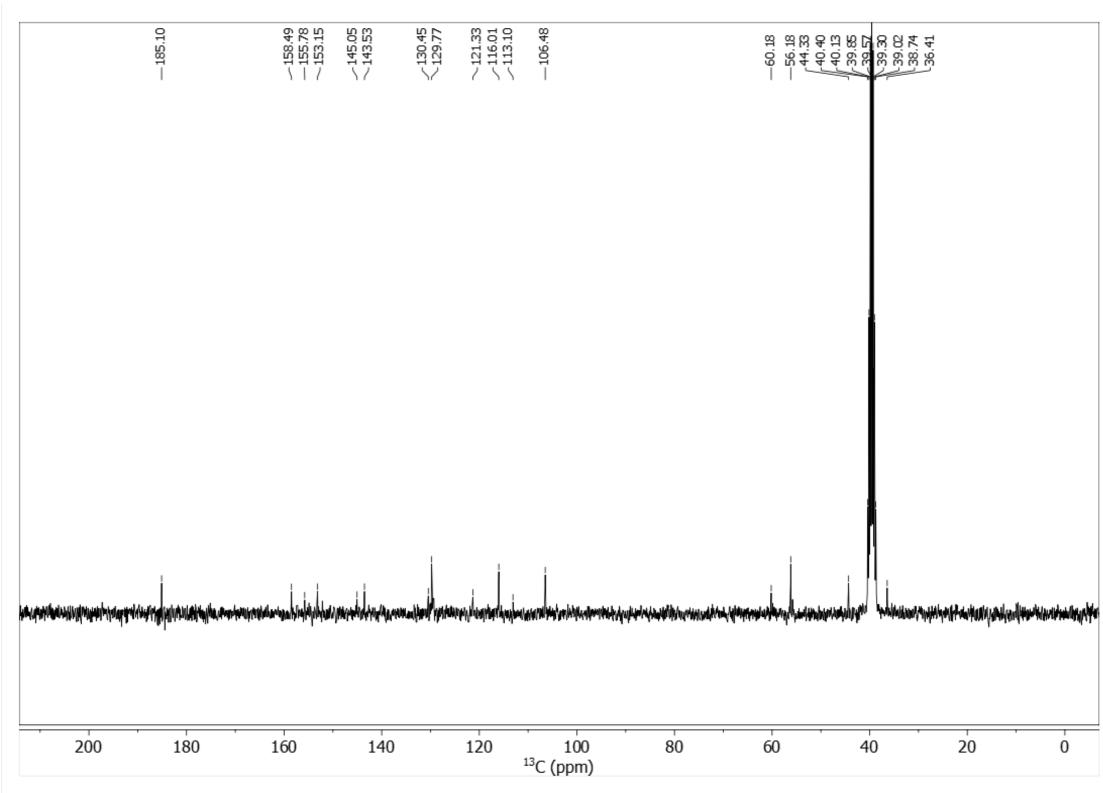

185.10
158.49
155.78
153.15
145.05
143.53
130.45
129.77
121.33
116.01
113.10
106.48
60.18
56.18
44.33
40.40
40.13
39.85
39.57
39.30
39.02
38.74
36.41
200
180
160
140
120
100
80
60
40
20
0
13C (ppm)

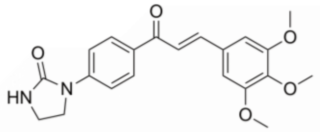

5

MS spectrum peak list

m/z	*Calc m/z*	Diff(ppm)	Abund	Formula	Ion
383.1597	383.1601	-1.14	605667	C21 H23 N2 O5	(M+H)+
383.4024			44515		
383.5154			3645		
383.6589			64035		
383.9031			3697		
383.9837			1561		
384.1622	384.1633	-3.08	119510	C21 H23 N2 O5	(M+H)+
384.4041			5785		
384.6618			4020		
385.1655	385.166	-1.23	13392	C21 H23 N2 O5	

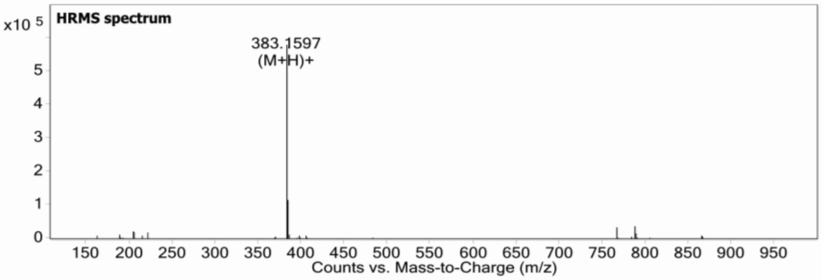

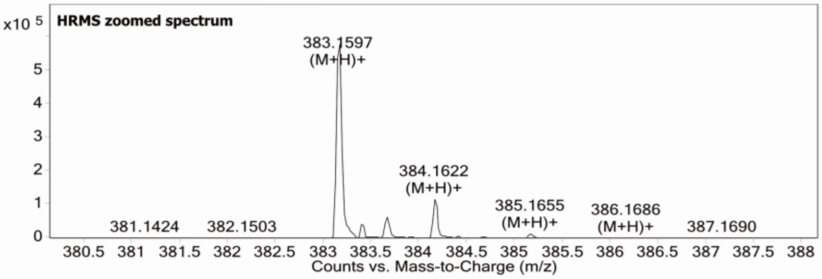

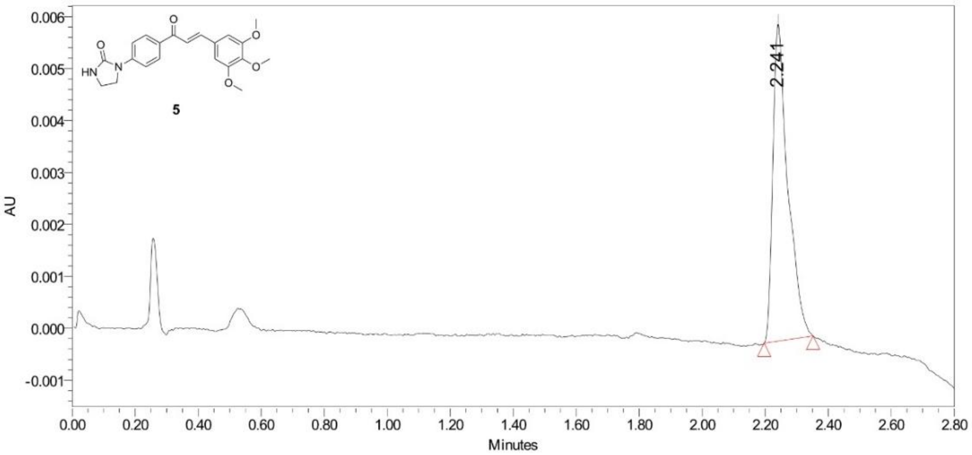

Peak Results

	RT	Area	Height	% Area	% Height
1	2.241	20219	6107	100.00	100.00

Compound 6

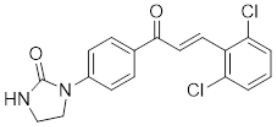
O
Cl
O
HN
N
Cl

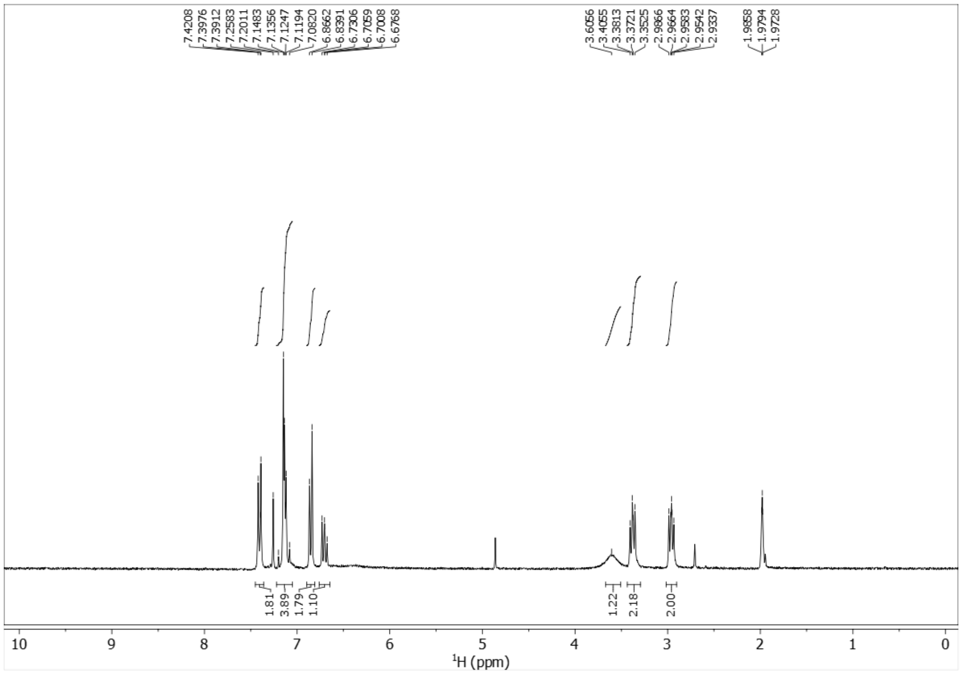
7.4208
7.3976
7.3912
7.2583
7.2011
7.1483
7.1356
7.1247
7.1194
7.0820
6.8662
6.8391
6.7306
6.7059
6.7008
6.6768
3.6056
3.4055
3.3813
3.3721
3.3525
2.9866
2.9664
2.9583
2.9542
2.9337
1.9858
1.9794
1.9728
1.81
3.89
1.79
1.10
1.22
2.18
2.00
10
9
8
7
6
5
4
3
2
1
0
^{1}H (ppm)

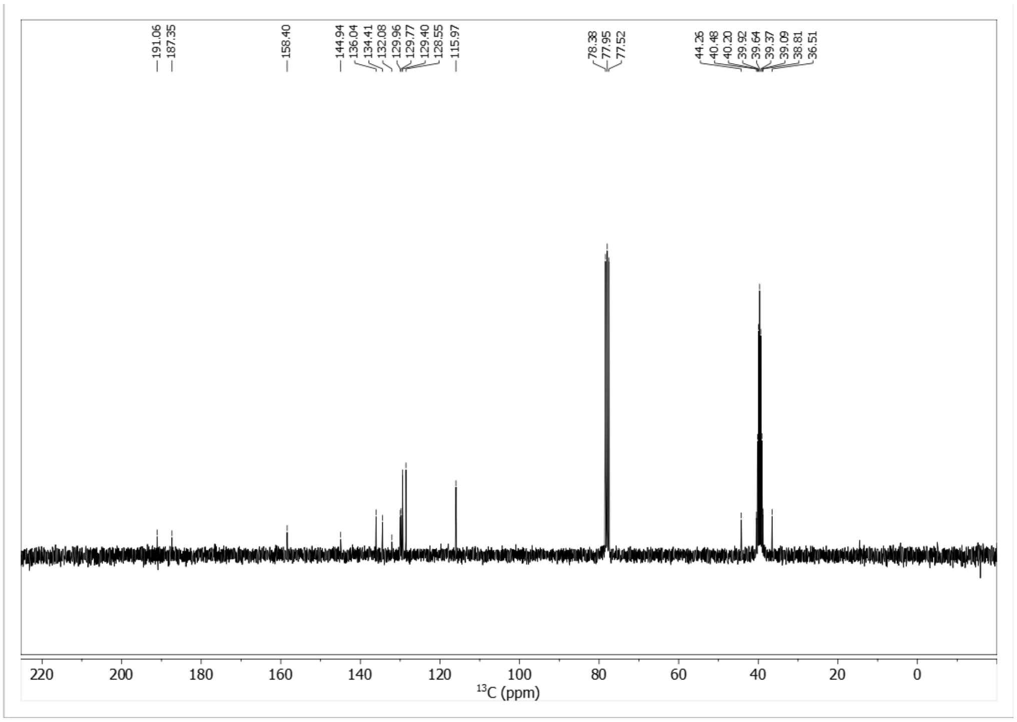
191.06
187.35
158.40
144.94
136.04
134.41
132.08
129.96
129.77
129.40
128.55
115.97
78.38
77.95
77.52
44.26
40.48
40.20
39.92
39.64
39.37
39.09
38.81
36.51
220
200
180
160
140
120
100
80
60
40
20
0
^{13}C (ppm)

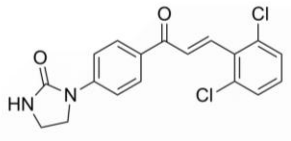

6

MS spectrum peak list

m/z	Calc m/z	Diff(ppm)	Abund	Formula	Ion
361.0493	361.0505	-3.22	515398	C18 H15 Cl2 N2 O2	(M+H)+
361.2838			37047		
361.519			3299		
362.0522	362.0537	-4.07	89795	C18 H15 Cl2 N2 O2	(M+H)+
362.2878			3475		
363.0466	363.0479	-3.54	346363	C18 H15 Cl2 N2 O2	(M+H)+
363.2831			22397		
364.0494	364.0508	-3.97	57941	C18 H15 Cl2 N2 O2	(M+H)+
365.0443	365.0458	-3.98	47465	C18 H15 Cl2 N2 O2	
366.0465	366.0483	-4.82	7898	C18 H15 Cl2 N2 O2	

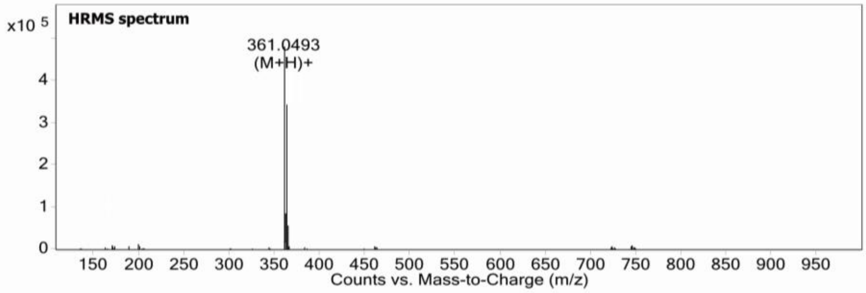

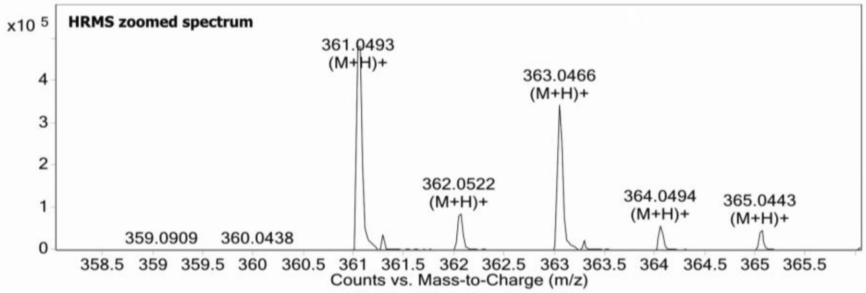

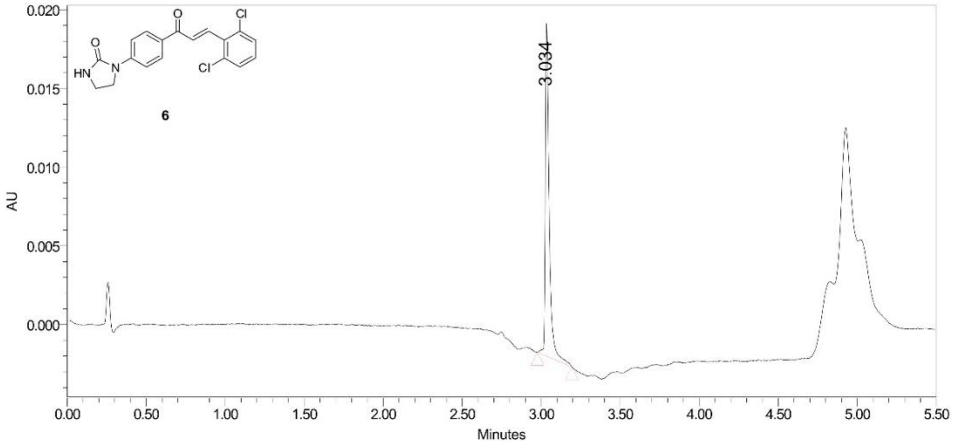

Peak Results

	RT	Area	Height	% Area	% Height
1	3.034	35759	21200	100.00	100.00

Compound 7

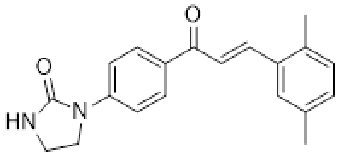

O
O
HN
N

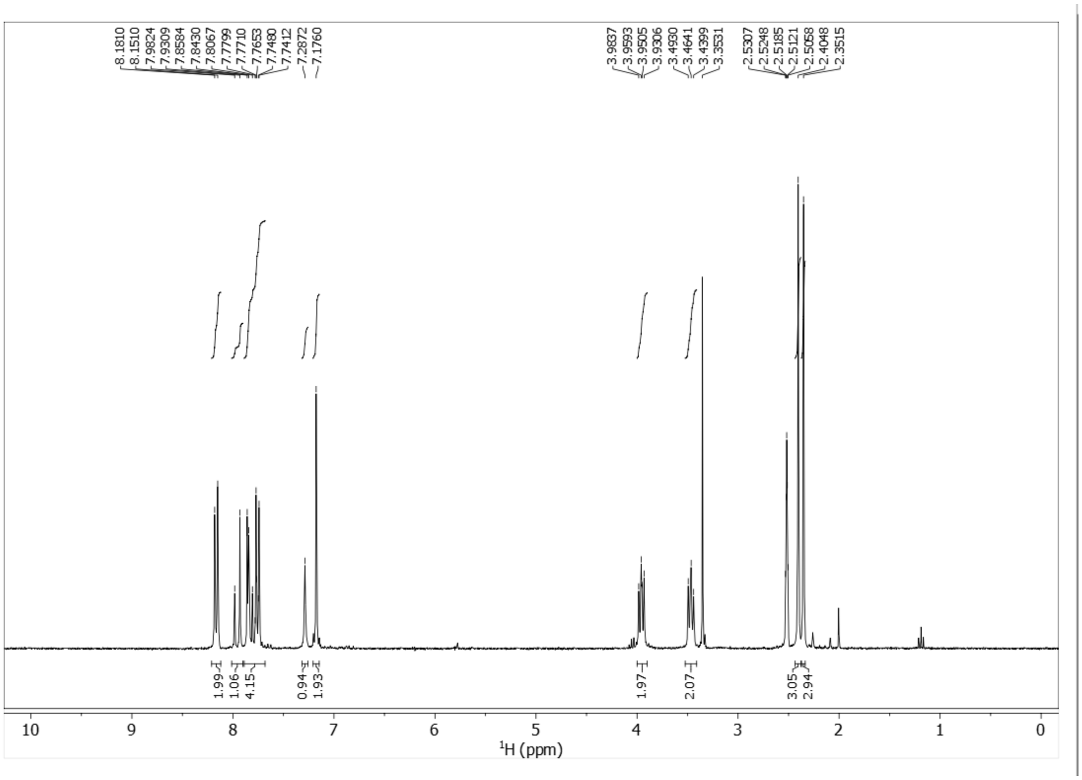

8.1810
8.1510
7.9824
7.9309
7.8584
7.8430
7.8067
7.7799
7.7710
7.7653
7.7480
7.7412
7.2872
7.1760
3.9837
3.9593
3.9505
3.9306
3.4930
3.4641
3.4399
3.3531
2.5307
2.5248
2.5185
2.5121
2.5058
2.4048
2.3515
1.99
1.06
4.15
0.94
1.93
1.97
2.07
3.05
2.94
10 9 8 7 6 5 4 3 2 1 0
^{1}H (ppm)

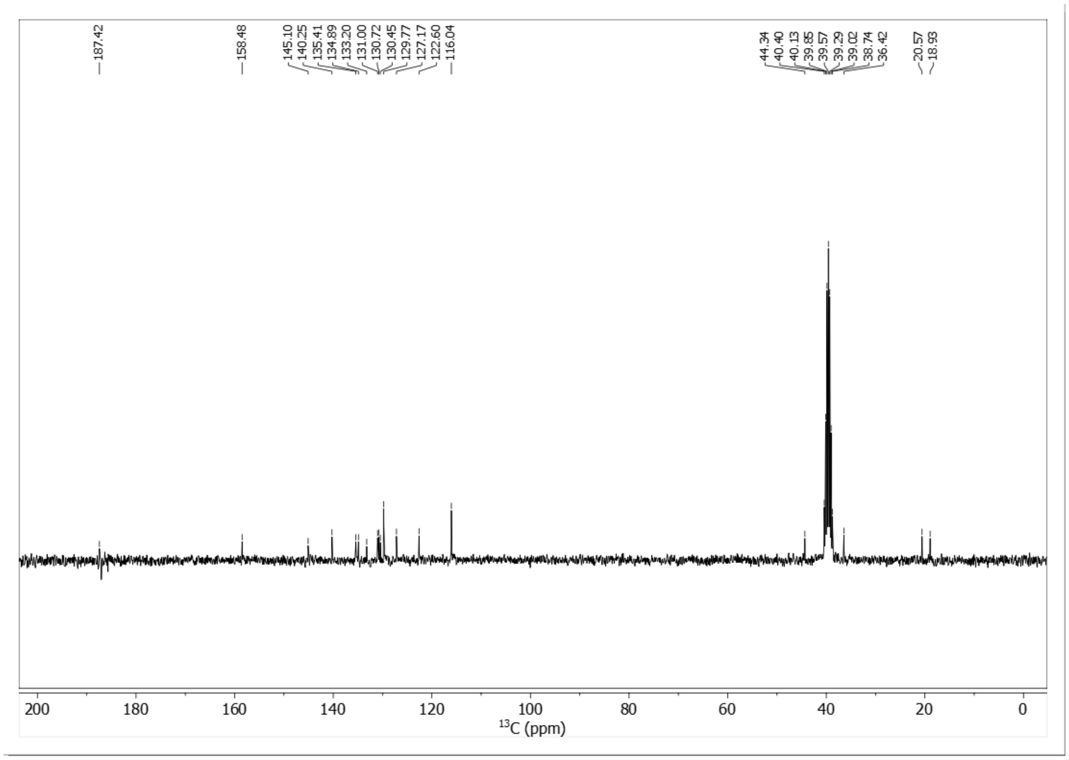

187.42
158.48
145.10
140.25
135.41
134.89
133.20
131.00
130.72
130.45
129.77
127.17
122.60
116.04
44.34
40.40
40.13
39.85
39.57
39.29
39.02
38.74
36.42
20.57
18.93
200 180 160 140 120 100 80 60 40 20 0
^{13}C (ppm)

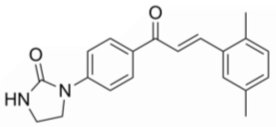

7

MS spectrum peak list

m/z	*Calc m/z*	Diff(ppm)	Abund	Formula	Ion
321.1594	321.1598	-1.09	1091952	C20 H21 N2 O2	(M+H)+
321.3796			115121		
321.4997			8603		
321.6024			14016		
321.6878			4589		
321.8261			4315		
322.1605	322.1629	-7.46	264477	C20 H21 N2 O2	(M+H)+
322.3821			19085		
322.4832			3139		
323.1635	323.1658	-7.17	27397	C20 H21 N2 O2	

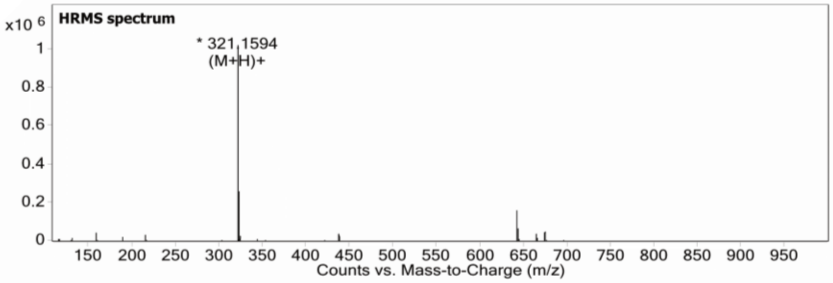

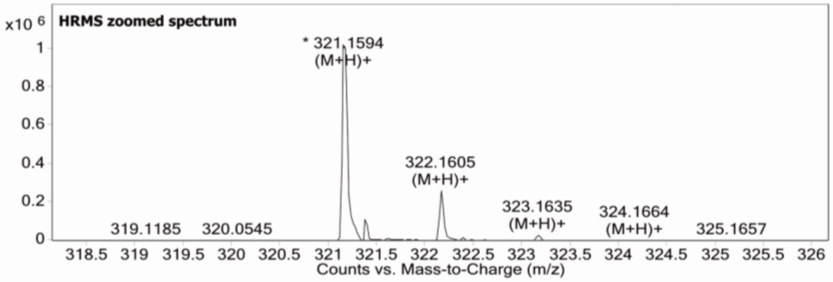

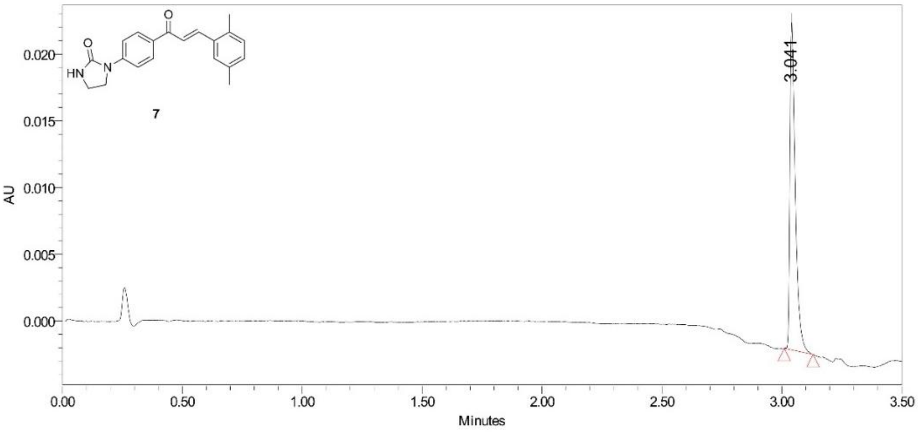

Peak Results

	RT	Area	Height	% Area	% Height
1	3.041	38275	24559	100.00	100.00

Compound 8

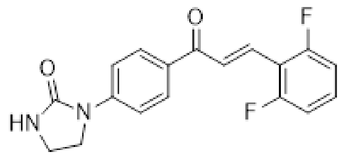

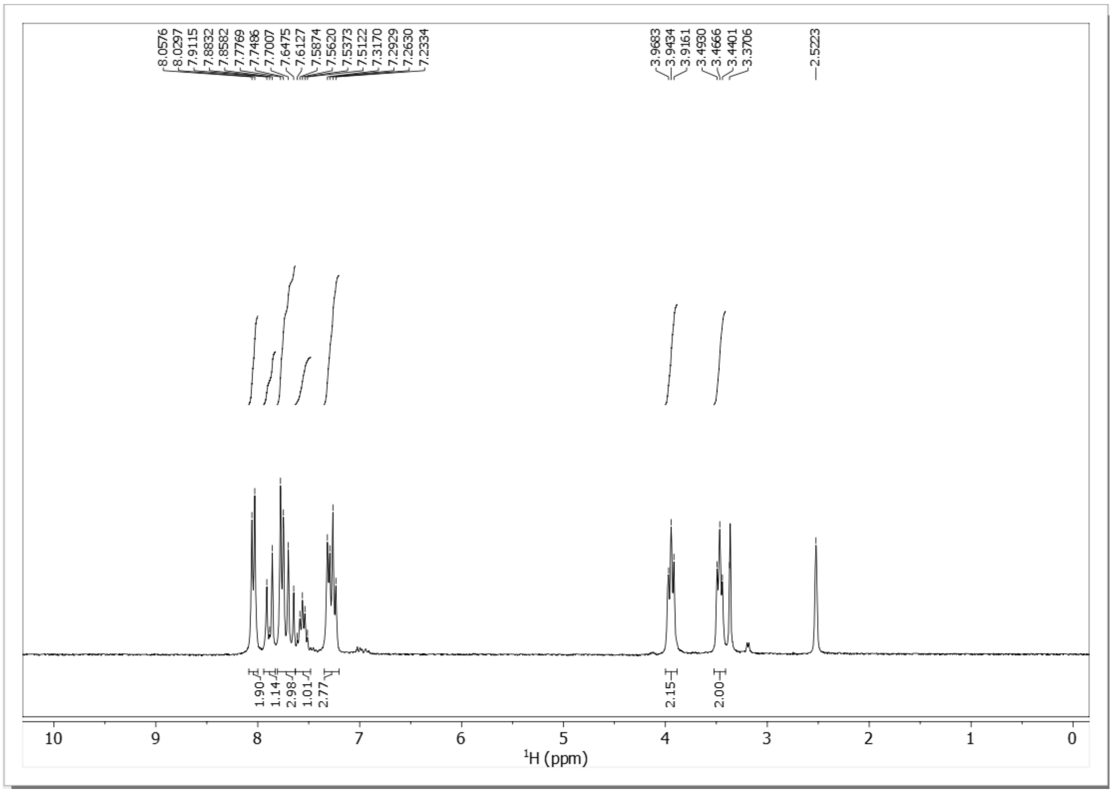

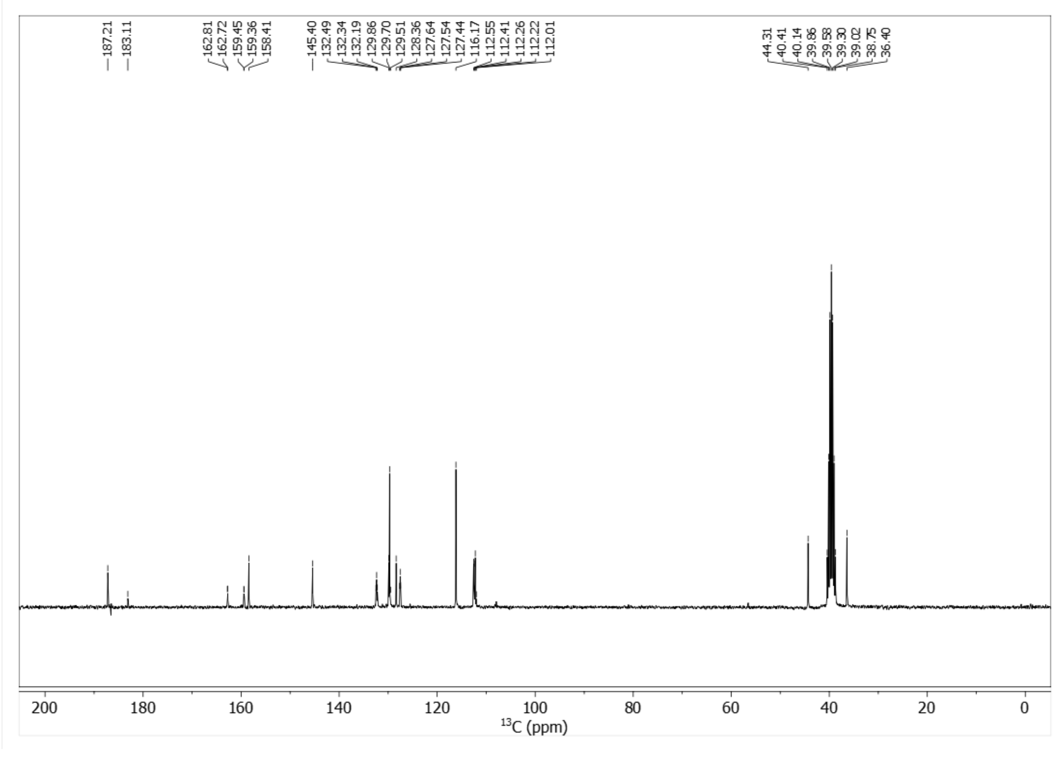

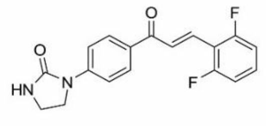

8

MS spectrum peak list

m/z	*Calc m/z*	Diff(ppm)	Abund	Formula	Ion
329.1093	329.1096	-1.09	1182689	C18 H15 F2 N2 O2	(M+H)+
329.3322			126351		
329.4552			9848		
329.5572			15469		
329.6661			5037		
329.7844			4485		
330.1103	330.1128	-7.56	257318	C18 H15 F2 N2 O2	(M+H)+
330.3382			16193		
330.4369			3215		
331.1131	331.1156	-7.28	24990	C18 H15 F2 N2 O2	

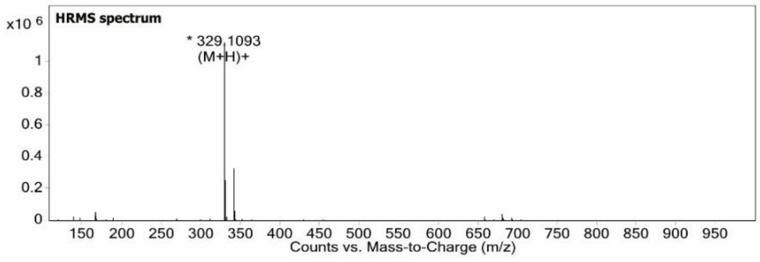

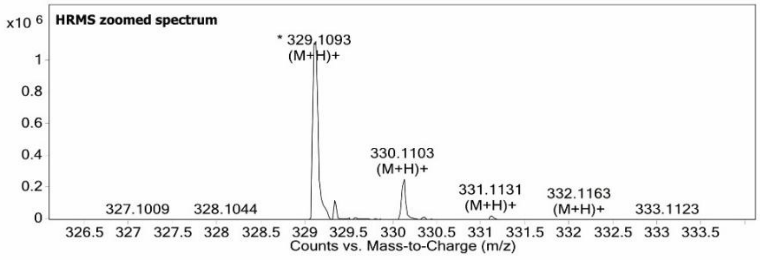

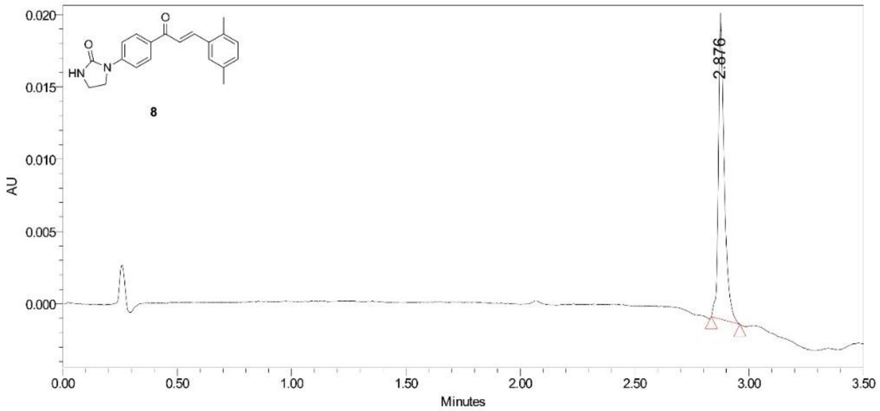

Peak Results

	RT	Area	Height	% Area	% Height
1	2.876	37891	20571	100.00	100.00
